# Supplementary figures and images for: Global, regional, and national burden of cardiomyopathy (including alcoholic cardiomyopathy and others) from 1990 to 2021: An analysis of data from the global burden of disease study 2021 and forecast to 2040
Source: PLoS One. 2026 Jan 30;21(1):e0341687. doi: 10.1371/journal.pone.0341687 (PMC12858021; doi:10.1371/journal.pone.0341687)

**
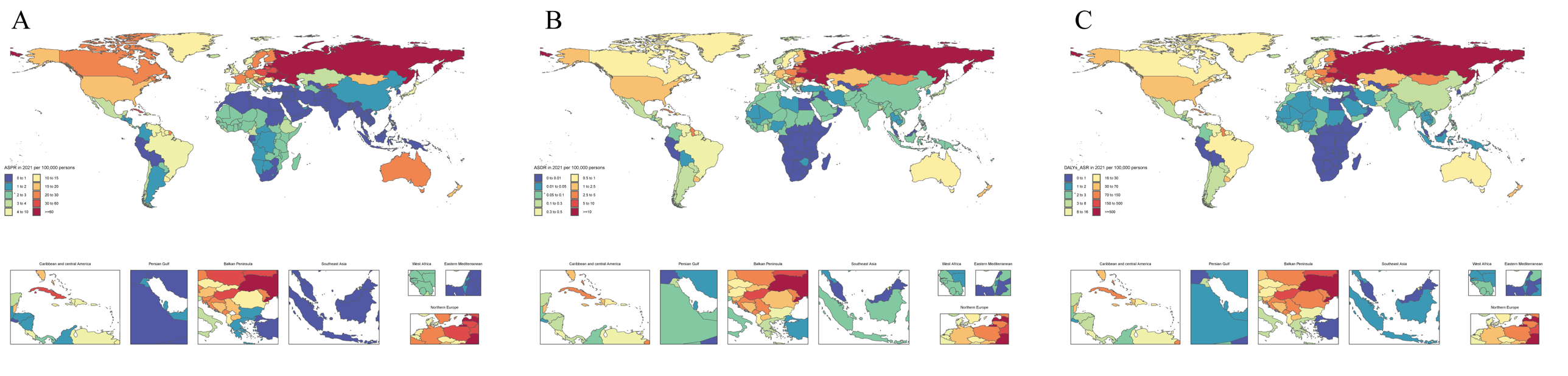
S6 Fig.** **ASRs of other cardiomyopathy in 204 countries and territories in 2021.** (A) ASPRs. (B) ASDRs. (C) ASR_DALYs.

Supplement: S6 Fig — (A) ASPRs. (B) ASDRs. (C) ASR_DALYs. (DOCX) [file pone.0341687.s006.docx]

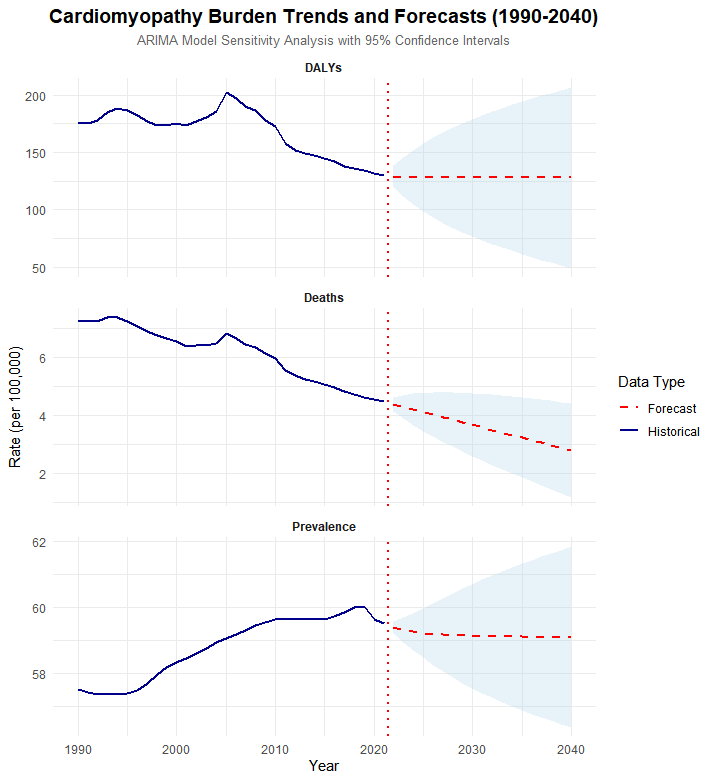


**S11 Fig.** **Global disease trends predictions of total cardiomyopathy from 2021 to 2040 using ARIMA.**

Supplement: S11 Fig — (DOCX) [file pone.0341687.s011.docx]
