# Supplementary material for: Global, regional, and national burden of cardiomyopathy (including alcoholic cardiomyopathy and others) from 1990 to 2021: An analysis of data from the global burden of disease study 2021 and forecast to 2040
Source: PLoS One. 2026 Jan 30;21(1):e0341687. doi: 10.1371/journal.pone.0341687 (PMC12858021; doi:10.1371/journal.pone.0341687)
Supplement: S8 Fig — (A) ASPRs. (B) ASDRs. (C) ASR_DALYs. (DOCX) [file pone.0341687.s008.docx]

**
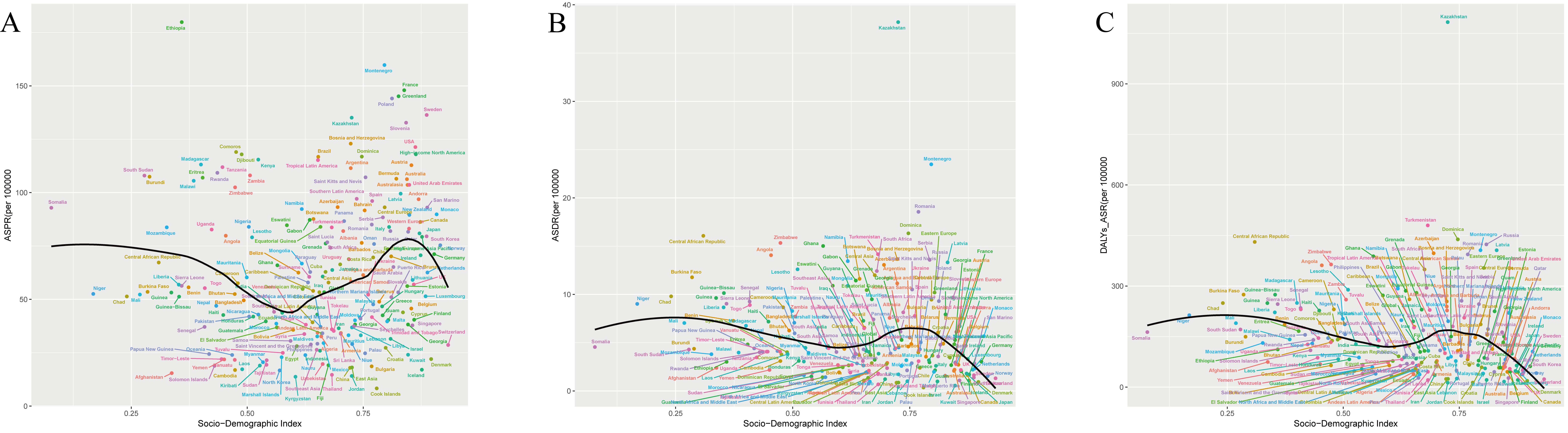
S8 Fig.** **Socio-demographic index trends with ASRs of other cardiomyopathy in 204 countries and territories in 2021.** (A) ASPRs. (B) ASDRs. (C) ASR_DALYs.
