# Supplementary material for: Global, regional, and national burden of cardiomyopathy (including alcoholic cardiomyopathy and others) from 1990 to 2021: An analysis of data from the global burden of disease study 2021 and forecast to 2040
Source: PLoS One. 2026 Jan 30;21(1):e0341687. doi: 10.1371/journal.pone.0341687 (PMC12858021; doi:10.1371/journal.pone.0341687)
Supplement: S1 Table — (DOCX) [file pone.0341687.s012.docx]

**S1 Table.** **1990–2021 Global and regional prevalence trends in alcoholic cardiomyopathy burden.**

| location | Alcoholic Cardiomyopathy Prevalence (95% UI) | | | | |
| --- | --- | --- | --- | --- | --- |
|  | Number_1990 | ASR per 100,000_1990 | Number_2021 | ASR per 100,000_2021 | EAPC_95% CI |
| Global | 356476.1 (301779.1–421491.9) | 7.9 (6.7–9.4) | 528429 (439582.3–639167.4) | 6.2 (5.1–7.4) | −0.59 (−0.83 to −0.35) |
| High SDI | 154582 (130952.7–182647.5) | 15.3 (12.9–18.1) | 224723.8 (186506.9–270838) | 14.2 (11.9–17) | 0.07 (−0.16 to 0.31) |
| High-middle SDI | 161878.5 (136339.8–191751.6) | 15.3 (13–18.1) | 212126.1 (174519.6–259347.4) | 12.2 (10.2–14.7) | −0.58 (−0.83 to −0.33) |
| Middle SDI | 21386.3 (17684.5–25384.8) | 1.5 (1.3–1.7) | 50120.7 (41233.3–60704.1) | 1.8 (1.5–2.2) | 0.83 (0.67–0.99) |
| Low-middle SDI | 12158.8 (10174.7–14469) | 1.5 (1.2–1.7) | 24910.5 (20384.1–30411.5) | 1.4 (1.2–1.7) | −0.21 (−0.29 to −0.14) |
| Low SDI | 6018.9 (4576.4–7890.1) | 1.8 (1.4–2.3) | 15517.5 (11698.4–20425.7) | 1.9 (1.5–2.4) | 0.22 (0.17–0.27) |
| Andean Latin America | 8.5 (6.3–11.3) | 0 (0–0.1) | 27.7 (20.2–37.7) | 0 (0–0.1) | 1.41 (0.92–1.9) |
| Australasia | 3031.4 (2482.6–3674.5) | 13.4 (11–16.3) | 11895.7 (9653–14308.5) | 26.6 (21.8–31.8) | 2.2 (1.73–2.67) |
| Caribbean | 1385 (1141.4–1684) | 4.6 (3.8–5.6) | 9445.3 (7741.6–11510.4) | 18 (14.8–21.9) | 5.65 (5.07–6.23) |
| Central Asia | 2201.8 (1778.1–2697.8) | 3.9 (3.2–4.8) | 5182 (4163.5–6386.5) | 5.4 (4.4–6.6) | 1.21 (1–1.42) |
| Central Europe | 21465.3 (17902.5–25570.7) | 15.6 (13.1–18.5) | 42372.2 (35271.7–50685.5) | 25 (21.1–29.5) | 2.15 (1.95–2.34) |
| Central Latin America | 3426.7 (2844.5–4077) | 2.7 (2.3–3.1) | 7975.7 (6594.5–9617.4) | 3 (2.5–3.6) | 0.16 (0.06–0.27) |
| Central Sub-Saharan Africa | 657.5 (477.3–887.7) | 1.9 (1.4–2.5) | 1484.7 (1092.9–2035) | 1.6 (1.2–2.2) | −0.4 (−0.49 to −0.31) |
| East Asia | 7047.3 (5870.5–8428.5) | 0.6 (0.5–0.7) | 30311.5 (24088.8–37517.3) | 1.6 (1.3–2) | 4.08 (3.68–4.48) |
| Eastern Europe | 142046.8 (119220.8–168869.6) | 56.5 (47.8–66.8) | 173042.7 (142599.1–211308.7) | 62.5 (51.9–75) | 0.5 (0.22–0.79) |
| Eastern Sub-Saharan Africa | 3348.7 (2436–4504.3) | 2.6 (2–3.5) | 8012.5 (5774.5–10964) | 2.5 (1.9–3.4) | −0.21 (−0.26 to −0.17) |
| High-income Asia Pacific | 13834.8 (11204.3–17351.5) | 6.7 (5.5–8.4) | 13148.4 (10795.1–16170.6) | 5 (4–6.1) | −1.19 (−1.28 to −1.1) |
| High-income North America | 62597.6 (51831.3–75371.3) | 19.7 (16.4–23.8) | 89214.3 (74203.6–108043.5) | 17.4 (14.6–20.9) | −0.49 (−0.6 to −0.38) |
| North Africa and Middle East | 897.2 (751.4–1087.4) | 0.4 (0.3–0.5) | 2524.4 (2072.5–3153.2) | 0.4 (0.4–0.5) | 0.24 (0.14–0.34) |
| Oceania | 10.4 (8.4–12.7) | 0.2 (0.2–0.3) | 21.1 (17–25.8) | 0.2 (0.2–0.3) | −0.62 (−0.69 to −0.56) |
| South Asia | 4589.7 (3835–5525.5) | 0.6 (0.5–0.8) | 11199 (9043.9–13681.5) | 0.7 (0.5–0.8) | 0.22 (0.18–0.25) |
| Southeast Asia | 1209.6 (994.7–1466.5) | 0.4 (0.3–0.4) | 3219.2 (2644.3–3943) | 0.4 (0.4–0.5) | 0.54 (0.46–0.62) |
| Southern Latin America | 3478.7 (2843.6–4230.3) | 7.4 (6–9) | 2105.5 (1704.6–2586.9) | 2.7 (2.2–3.3) | −3.88 (−4.16 to −3.59) |
| Southern Sub-Saharan Africa | 381 (246.8–556.8) | 0.7 (0.5–1) | 451.5 (292.9–643.1) | 0.6 (0.4–0.8) | −1.02 (−1.12 to −0.92) |
| Tropical Latin America | 15372.3 (12580.3–18309.1) | 11.9 (9.8–14.1) | 19237.2 (15573.6–23399) | 7.5 (6–9.1) | −2.13 (−2.35 to −1.92) |
| Western Europe | 66147.4 (54655.7–78957.6) | 13.7 (11.5–16.3) | 88260.6 (71202.7–108293.2) | 13.4 (11–16.1) | 0.53 (0.12–0.95) |
| Western Sub-Saharan Africa | 3338.5 (2496.4–4462) | 2.6 (2–3.4) | 9298 (6788.4–12252.1) | 2.8 (2.1–3.6) | 0.18 (0.05–0.31) |
