# Supplementary material for: Global, regional, and national burden of cardiomyopathy (including alcoholic cardiomyopathy and others) from 1990 to 2021: An analysis of data from the global burden of disease study 2021 and forecast to 2040
Source: PLoS One. 2026 Jan 30;21(1):e0341687. doi: 10.1371/journal.pone.0341687 (PMC12858021; doi:10.1371/journal.pone.0341687)
Supplement: S2 Table — (DOCX) [file pone.0341687.s013.docx]

**S2 Table.** 1**990–2021 Global and regional mortality trends in alcoholic cardiomyopathy burden.**

| location | Alcoholic Cardiomyopathy Deaths (95% UI) | | | | |
| --- | --- | --- | --- | --- | --- |
|  | Number_1990 | ASR per 100,000_1990 | Number_2021 | ASR per 100,000_2021 | EAPC_95% CI |
| Global | 47072.9 (44168.2–49765.4) | 1.2 (1.1–1.2) | 64011.4 (56292.6–69518.7) | 0.7 (0.7–0.8) | −1.72 (−2.74 to −0.7) |
| High SDI | 15436.9 (14326–16389.3) | 1.4 (1.3–1.5) | 14211.1 (13109.3–15062.2) | 0.8 (0.7–0.8) | −2.08 (−2.27 to −1.89) |
| High-middle SDI | 27695.4 (25732.4–29522.5) | 2.8 (2.6–3) | 43096.2 (38338.7–47326.1) | 2.4 (2.1–2.6) | −0.98 (−2.33 to 0.38) |
| Middle SDI | 2300.1 (1777.8–3032.1) | 0.2 (0.2–0.3) | 3820.7 (2166.6–5138.1) | 0.1 (0.1–0.2) | −1.39 (−1.55 to −1.24) |
| Low-middle SDI | 1308.2 (751–2120.1) | 0.2 (0.1–0.3) | 2313.6 (1251.9–3975.7) | 0.1 (0.1–0.3) | −1.04 (−1.17 to −0.9) |
| Low SDI | 255.5 (64–535.3) | 0.1 (0–0.2) | 452.8 (105–1047.4) | 0.1 (0–0.2) | −0.84 (−0.93 to −0.76) |
| Andean Latin America | 2 (0.8–3.5) | 0 (0–0) | 2.8 (0.8–4.5) | 0 (0–0) | −2.04 (−2.6 to −1.48) |
| Australasia | 276.9 (252.9–303) | 1.2 (1.1–1.3) | 433.4 (401.7–468.7) | 0.9 (0.8–1) | −0.96 (−1.5 to −0.42) |
| Caribbean | 155.4 (108.4–234.6) | 0.6 (0.4–0.9) | 998.2 (799.8–1207.9) | 1.9 (1.5–2.3) | 5.28 (4.69–5.87) |
| Central Asia | 383.8 (325.8–461.5) | 0.8 (0.6–0.9) | 957.7 (788.7–1186.7) | 1 (0.9–1.3) | 1.19 (0.51–1.88) |
| Central Europe | 4004.9 (3622.2–4385.6) | 2.9 (2.6–3.1) | 5346.3 (4287.7–6113.3) | 2.7 (2.1–3.1) | 0.07 (−0.09 to 0.23) |
| Central Latin America | 207.1 (191–227.7) | 0.2 (0.2–0.2) | 478.4 (422–540.9) | 0.2 (0.2–0.2) | −1.31 (−1.65 to −0.98) |
| Central Sub-Saharan Africa | 1 (0.2–5) | 0 (0–0) | 2 (0.3–12) | 0 (0–0) | −0.99 (−1.03 to −0.95) |
| East Asia | 573 (253.3–1216.5) | 0.1 (0–0.1) | 1999.6 (426.3–3149.7) | 0.1 (0–0.2) | 2.07 (1.79–2.35) |
| Eastern Europe | 24150.1 (22428.6–25915.4) | 9.3 (8.6–9.9) | 38834.7 (34484.1–42737) | 13.1 (11.6–14.4) | 0.65 (−0.9 to 2.22) |
| Eastern Sub-Saharan Africa | 1.7 (0.3–8.6) | 0 (0–0) | 2.9 (0.5–23.3) | 0 (0–0) | −1.23 (−1.34 to −1.12) |
| High-income Asia Pacific | 672.8 (625–734.6) | 0.3 (0.3–0.4) | 489.1 (442.6–532.4) | 0.1 (0.1–0.1) | −3.29 (−3.39 to −3.19) |
| High-income North America | 4778 (4403.8–5116.5) | 1.4 (1.3–1.5) | 6014.8 (5590.1–6391.3) | 1 (0.9–1.1) | −1.22 (−1.43 to −1.02) |
| North Africa and Middle East | 102.4 (23.9–201.1) | 0.1 (0–0.1) | 179.3 (51–350.6) | 0 (0–0.1) | −1.42 (−1.51 to −1.33) |
| Oceania | 2.8 (0.3–5.5) | 0.1 (0–0.1) | 5 (0.8–10.5) | 0.1 (0–0.1) | −1.22 (−1.31 to −1.12) |
| South Asia | 712.7 (152.2–1736.8) | 0.1 (0–0.3) | 1450.7 (268.9–3395) | 0.1 (0–0.2) | −0.5 (−0.56 to −0.44) |
| Southeast Asia | 169.7 (39.6–336) | 0.1 (0–0.1) | 365.1 (67.2–605.5) | 0.1 (0–0.1) | −0.6 (−0.74 to −0.47) |
| Southern Latin America | 582 (504–668) | 1.2 (1.1–1.4) | 175 (155.5–194.6) | 0.2 (0.2–0.2) | −6.69 (−7.12 to −6.26) |
| Southern Sub-Saharan Africa | 2.6 (0.5–6.3) | 0 (0–0) | 3.4 (0.7–9.2) | 0 (0–0) | −0.72 (−1.2 to −0.25) |
| Tropical Latin America | 1740.2 (1608.9–1924.9) | 1.6 (1.5–1.8) | 1185.8 (1102.7–1269.7) | 0.4 (0.4–0.5) | −5.23 (−5.67 to −4.78) |
| Western Europe | 8401.2 (7572.1–9156.5) | 1.5 (1.4–1.7) | 4939.3 (4446.4–5379.1) | 0.6 (0.5–0.6) | −3.3 (−3.54 to −3.07) |
| Western Sub-Saharan Africa | 152.7 (20.5–282.1) | 0.2 (0–0.3) | 147.9 (22.5–382.2) | 0.1 (0–0.2) | −3.64 (−3.93 to −3.35) |
