# Supplementary material for: Global, regional, and national burden of cardiomyopathy (including alcoholic cardiomyopathy and others) from 1990 to 2021: An analysis of data from the global burden of disease study 2021 and forecast to 2040
Source: PLoS One. 2026 Jan 30;21(1):e0341687. doi: 10.1371/journal.pone.0341687 (PMC12858021; doi:10.1371/journal.pone.0341687)
Supplement: S3 Table — (DOCX) [file pone.0341687.s014.docx]

**S3 Table.** **1990–2021 Global and regional DALYs trends in alcoholic cardiomyopathy burden.**

| location | Alcoholic Cardiomyopathy DALYs (95% UI) | | | | |
| --- | --- | --- | --- | --- | --- |
|  | Number_1990 | ASR per 100,000_1990 | Number_2021 | ASR per 100,000_2021 | EAPC_95% CI |
| Global | 1561763.3 (1456364.6–1653052.7) | 35.9 (33.5–38) | 2185527.6 (1928589.3–2368735.7) | 25.3 (22.4–27.5) | −1.44 (−2.61 to −0.27) |
| High SDI | 432923.8 (405370.8–455451.7) | 41.6 (39–43.7) | 404815.2 (377172.9–427496.9) | 24.3 (22.7–25.6) | −1.74 (−1.95 to −1.53) |
| High-middle SDI | 981366.5 (909231.6–1049080.8) | 93.7 (86.7–100.2) | 1546321.6 (1382322–1696613.6) | 88.3 (79–96.7) | −0.72 (−2.18 to 0.76) |
| Middle SDI | 87801.5 (68248.9–114763.8) | 6.6 (5.2–8.7) | 130953.1 (74830.6–173094.1) | 4.6 (2.7–6.1) | −1.49 (−1.67 to −1.32) |
| Low-middle SDI | 48262.1 (28738.7–76454.3) | 6.3 (3.7–10.1) | 83302.8 (48120.9–137494.7) | 4.9 (2.8–8.2) | −0.97 (−1.11 to −0.82) |
| Low SDI | 9245.8 (2705.3–18790.6) | 3.3 (1–6.8) | 16771.9 (5225.2–37948) | 2.6 (0.8–5.8) | −0.88 (−0.94 to −0.82) |
| Andean Latin America | 36.5 (15.1–61.2) | 0.2 (0.1–0.3) | 49.7 (15.7–80.4) | 0.1 (0–0.1) | −1.87 (−2.45 to −1.29) |
| Australasia | 7966.9 (7348–8654.3) | 35.5 (32.8–38.5) | 12459.2 (11570.5–13526.5) | 28.2 (26.2–30.6) | −0.64 (−1.16 to −0.12) |
| Caribbean | 5456.2 (3825.5–8086) | 19.3 (13.5–28.7) | 30772.6 (24177.5–37784.7) | 57.8 (45.2–71.3) | 4.9 (4.35–5.46) |
| Central Asia | 13858.1 (11872.4–16559.1) | 26 (22.2–31.2) | 35979.9 (29711.3–44305.1) | 36.7 (30.4–45.2) | 1.41 (0.74–2.09) |
| Central Europe | 111046.2 (99952.8–122127.2) | 77.1 (69.4–84.9) | 149199 (119377.9–170872.8) | 81.7 (65.1–93.8) | 0.46 (0.24–0.67) |
| Central Latin America | 7771 (7159.8–8524.6) | 7.2 (6.6–7.9) | 17576.4 (15511.6–19757.7) | 6.6 (5.8–7.4) | −0.9 (−1.2 to −0.6) |
| Central Sub-Saharan Africa | 97.2 (54.5–235.7) | 0.3 (0.2–0.8) | 209.7 (115.2–595.2) | 0.2 (0.1–0.7) | −0.63 (−0.69 to −0.57) |
| East Asia | 22432.4 (9942.4–47615) | 2.1 (1–4.4) | 69749.7 (16481.7–109076.5) | 3.6 (0.8–5.6) | 2.47 (2.18–2.76) |
| Eastern Europe | 883100.4 (816271.9–951221.1) | 339.3 (313.7–364.9) | 1426959.2 (1272059.1–1567982.1) | 510.5 (456.7–560.3) | 0.78 (−0.85 to 2.43) |
| Eastern Sub-Saharan Africa | 376.6 (224.2–671) | 0.3 (0.2–0.6) | 860.7 (501.4–1620.2) | 0.3 (0.2–0.6) | −0.41 (−0.45 to −0.36) |
| High-income Asia Pacific | 23591.2 (21976.6–25712.4) | 11.5 (10.7–12.5) | 13652.5 (12385.9–14806.5) | 4.5 (4.1–4.8) | −3.33 (−3.43 to −3.23) |
| High-income North America | 140939 (131196.9–149687.8) | 43.6 (40.6–46.2) | 177117.9 (166935.4–187218) | 32.9 (31–34.7) | −0.98 (−1.14 to −0.81) |
| North Africa and Middle East | 3328.6 (825.3–5912.2) | 1.7 (0.4–3.2) | 5724.4 (1684.9–10424) | 1.1 (0.3–2) | −1.52 (−1.61 to −1.42) |
| Oceania | 115.6 (14.1–236.7) | 2.5 (0.3–5) | 206 (34.3–419.9) | 1.9 (0.3–3.9) | −1.17 (−1.29 to −1.06) |
| South Asia | 25173.5 (5778.3–61381.6) | 3.5 (0.8–8.5) | 47908 (9929.7–112144.6) | 2.9 (0.6–6.7) | −0.62 (−0.66 to −0.58) |
| Southeast Asia | 6602.1 (1540.3–11985.3) | 2 (0.5–3.8) | 13674.5 (2550.3–21947.4) | 1.8 (0.3–2.9) | −0.44 (−0.55 to −0.33) |
| Southern Latin America | 17883.3 (15617.7–20507) | 38 (33.2–43.6) | 5024.7 (4484.6–5557.2) | 6.1 (5.5–6.8) | −6.72 (−7.14 to −6.3) |
| Southern Sub-Saharan Africa | 132.7 (50.9–278.3) | 0.3 (0.1–0.6) | 172.1 (72.5–468.6) | 0.3 (0.1–0.6) | −0.65 (−0.95 to −0.34) |
| Tropical Latin America | 67688.2 (62670.5–74420.4) | 57.5 (53.3–63.4) | 44412 (41533.2–47434.8) | 16.8 (15.7–18) | −5.09 (−5.55 to −4.63) |
| Western Europe | 218704.6 (197713.8–237175) | 42.9 (38.9–46.5) | 127835.8 (116945.2–137643.6) | 17.6 (16.2–18.9) | −3.07 (−3.34 to −2.81) |
| Western Sub-Saharan Africa | 5462.8 (881–9931.8) | 5.4 (0.9–9.7) | 5983.8 (1532.2–14382.9) | 2.3 (0.6–5.5) | −3.38 (−3.66 to −3.1) |
