# Supplementary material for: Global, regional, and national burden of cardiomyopathy (including alcoholic cardiomyopathy and others) from 1990 to 2021: An analysis of data from the global burden of disease study 2021 and forecast to 2040
Source: PLoS One. 2026 Jan 30;21(1):e0341687. doi: 10.1371/journal.pone.0341687 (PMC12858021; doi:10.1371/journal.pone.0341687)
Supplement: S4 Table — (DOCX) [file pone.0341687.s015.docx]

**S4 Table.** **1990–2021 Global and regional prevalence trends in other cardiomyopathy burden.**

| location | Other Cardiomyopathy Prevalence (95% UI) | | | | |
| --- | --- | --- | --- | --- | --- |
|  | Number_1990 | ASR per 100,000_1990 | Number_2021 | ASR per 100,000_2021 | EAPC_95% CI |
| Global | 2285258.6 (1897989.8–2686423.6) | 49.6 (41.1–57.6) | 4223932.2 (3417357.4–5034257.6) | 53.3 (43.6–63.8) | 0.27 (0.25–0.29) |
| High SDI | 910452.8 (759247.6–1059346.1) | 101.3 (85.3–116.9) | 1223330.4 (1010577.8–1475220.5) | 93.3 (77.5–109.6) | −0.29 (−0.31 to −0.26) |
| High-middle SDI | 379719.7 (313316–446399.1) | 41.2 (33.9–49.3) | 647483.2 (525468.6–775670.6) | 46.6 (38.5–55.3) | 0.55 (0.49–0.61) |
| Middle SDI | 403498.6 (332435–486294.8) | 28.3 (23.4–33.2) | 901916.6 (725618.3–1091026.5) | 38.3 (31.2–45.9) | 1.05 (0.96–1.13) |
| Low-middle SDI | 345270.8 (284086.4–416983.4) | 38.2 (31.5–44.9) | 800306.7 (631222.4–978528) | 47.6 (38.1–57.2) | 0.69 (0.67–0.72) |
| Low SDI | 243542.6 (189876–311342.4) | 62.4 (48.8–78.1) | 646398.5 (497854.2–819271.3) | 73 (57.2–91.7) | 0.52 (0.51–0.54) |
| Andean Latin America | 10656.7 (8667–13165.8) | 30.2 (24.9–36.2) | 21869.9 (17302.2–26470.7) | 34.4 (27.4–41.6) | 0.58 (0.43–0.73) |
| Australasia | 21298.6 (17761.9–24819.1) | 106.6 (89.5–124.7) | 36371 (28886.8–43447.2) | 103.6 (83.1–125.5) | 0.21 (−0.02 to 0.43) |
| Caribbean | 15263.7 (12490.1–18262.8) | 45.6 (37.3–54.1) | 25974.2 (21013.5–31003.6) | 54 (43.6–64.4) | 0.49 (0.42–0.55) |
| Central Asia | 18607.1 (15302.4–22601.3) | 28 (23.2–33.1) | 51460.7 (42101–60821.7) | 57.9 (47.6–68.7) | 3.15 (2.89–3.41) |
| Central Europe | 98465 (80347.2–118175) | 79.5 (65.3–95) | 155384.3 (122245.5–194150.2) | 93.1 (76.5–112.4) | 0.67 (0.52–0.82) |
| Central Latin America | 57005.2 (46482.3–70062.5) | 34.9 (28.9–41.3) | 95571 (77116.5–116363) | 39.2 (31.9–47.7) | 0.39 (0.32–0.46) |
| Central Sub-Saharan Africa | 30396.9 (22663.2–40866.7) | 70.6 (51.7–92.4) | 86228.8 (63636.7–116837.3) | 78.6 (58.6–102.7) | 0.32 (0.29–0.35) |
| East Asia | 99645.1 (78589.9–123303) | 9.4 (7.6–11.4) | 269773.6 (210535.2–337384.2) | 18.1 (14.3–22.7) | 2.63 (2.34–2.92) |
| Eastern Europe | 77759.9 (62840.7–95781.7) | 38.3 (30.6–47.1) | 150141.8 (121248.4–181364.4) | 73.5 (59.8–90) | 2.59 (2.41–2.78) |
| Eastern Sub-Saharan Africa | 154905.5 (119600.9–197261.7) | 109.3 (84.5–140.3) | 394350.3 (306830.8–496748.2) | 124.1 (95.6–159.2) | 0.32 (0.29–0.35) |
| High-income Asia Pacific | 131383.6 (108949–155309.6) | 76.9 (63.7–92.3) | 194342.5 (157026–238660.3) | 79.3 (65.8–94.5) | 0.34 (0.25–0.42) |
| High-income North America | 412781.5 (337957.4–485477.6) | 143.1 (118.6–168.4) | 490329.9 (405043.5–594052.8) | 118 (97.8–141.5) | −0.84 (−0.9 to −0.77) |
| North Africa and Middle East | 107782.8 (87935.2–134254.1) | 30.1 (25.2–35.8) | 222481.3 (177916.3–275039.1) | 37.1 (30.2–45.4) | 0.7 (0.65–0.75) |
| Oceania | 1040.1 (820.5–1307.8) | 20.7 (16.5–25.2) | 2685.4 (2081–3399.5) | 23.9 (18.5–30.1) | 0.54 (0.5–0.57) |
| South Asia | 275130.4 (224850–327857.6) | 33.3 (27.4–39.1) | 707324.1 (561389.2–851612.7) | 44.1 (35.2–52.3) | 1.06 (1–1.12) |
| Southeast Asia | 71881.9 (58934.2–87137.7) | 23.2 (19.2–27.8) | 161148.2 (129923–196702.7) | 26.4 (21.5–32.2) | 0.28 (0.23–0.33) |
| Southern Latin America | 45571.6 (37399.1–54031.3) | 95.5 (78.6–114.8) | 71294 (58138.3–87063.4) | 97.1 (79.7–116.6) | 0.04 (−0.07 to 0.14) |
| Southern Sub-Saharan Africa | 32779 (25101.8–42456.2) | 74.7 (57.8–95.4) | 59173.4 (45574.3–75807.1) | 79.3 (60.6–101.2) | 0.12 (0.03–0.21) |
| Tropical Latin America | 129067.7 (107179.5–153316.9) | 109.2 (90.8–130.5) | 265449 (215717–318397.3) | 115.2 (94–139.5) | 0.02 (−0.06 to 0.09) |
| Western Europe | 398930.4 (331475.7–479952.8) | 92.2 (77.7–107.2) | 505907.5 (402197.3–616812.1) | 83.1 (68.8–97.4) | −0.36 (−0.49 to −0.23) |
| Western Sub-Saharan Africa | 94905.8 (72416.3–123751.5) | 64.9 (49–83.7) | 256671.2 (191888.8–340858.8) | 68.8 (51.3–89.8) | 0.13 (0.11–0.16) |
