# Supplementary material for: Global, regional, and national burden of cardiomyopathy (including alcoholic cardiomyopathy and others) from 1990 to 2021: An analysis of data from the global burden of disease study 2021 and forecast to 2040
Source: PLoS One. 2026 Jan 30;21(1):e0341687. doi: 10.1371/journal.pone.0341687 (PMC12858021; doi:10.1371/journal.pone.0341687)
Supplement: S5 Table — (DOCX) [file pone.0341687.s016.docx]

**S5 Table.** **1990–2021 Global and regional mortality trends in other cardiomyopathy burden.**

| location | Other Cardiomyopathy Deaths (95% UI) | | | | |
| --- | --- | --- | --- | --- | --- |
|  | Number_1990 | ASR per 100,000_1990 | Number_2021 | ASR per 100,000_2021 | EAPC_95% CI |
| Global | 207149.3 (180555.2–227935.3) | 6.1 (5.3–6.7) | 306262.2 (276191.5–333295.2) | 3.7 (3.4–4.1) | −1.62 (−1.72 to −1.52) |
| High SDI | 69003.8 (63648.1–72064.3) | 6.4 (5.9–6.7) | 66498.4 (58058.8–71903) | 2.9 (2.6–3.1) | −2.82 (−2.97 to −2.67) |
| High-middle SDI | 54267.3 (49356.3–57758.5) | 7.7 (6.8–8.2) | 76588.3 (70042.9–83125.1) | 4.2 (3.8–4.6) | −1.74 (−2 to −1.47) |
| Middle SDI | 32858.9 (27277.4–37671.8) | 3.6 (3–4.2) | 63626.6 (57920.4–69286.3) | 2.6 (2.4–2.9) | −1.31 (−1.5 to −1.11) |
| Low-middle SDI | 32288.7 (21757.4–42174.1) | 5.3 (3.7–7.2) | 65420.5 (55326.2–77964.5) | 4.9 (4.1–5.9) | −0.24 (−0.3 to −0.19) |
| Low SDI | 18329.1 (13456.9–25028.6) | 7.5 (5.5–11.1) | 33660.1 (25814.7–44564.2) | 6.4 (5–8.6) | −0.46 (−0.59 to −0.34) |
| Andean Latin America | 516.8 (440.3–592.3) | 2 (1.7–2.3) | 642.2 (532.4–777.4) | 1.1 (0.9–1.3) | −1.79 (−2.15 to −1.42) |
| Australasia | 1308.9 (1218.5–1397.5) | 6 (5.6–6.4) | 1223.4 (1066.6–1358) | 2.2 (1.9–2.4) | −2.81 (−3.57 to −2.05) |
| Caribbean | 1076.3 (851.7–1275.4) | 3.9 (3.2–4.6) | 1953.2 (1567.6–2336.9) | 3.7 (3–4.5) | −0.07 (−0.36 to 0.22) |
| Central Asia | 1810.7 (1542.9–2090.8) | 3.7 (3.2–4.3) | 9929.7 (8275–11681) | 12 (10.2–13.9) | 5.09 (3.94–6.25) |
| Central Europe | 18380.9 (17065.4–19871.4) | 14.7 (13.6–15.9) | 21842.4 (19646.4–23968.5) | 9.5 (8.5–10.4) | −1.59 (−1.89 to −1.28) |
| Central Latin America | 1967.9 (1863.4–2063.9) | 2.2 (2–2.3) | 3784.4 (3320.5–4307.4) | 1.6 (1.4–1.8) | −1.5 (−1.67 to −1.33) |
| Central Sub-Saharan Africa | 3626.1 (2297.1–5293.8) | 15.7 (9.6–24.9) | 7527.2 (4265.7–12001.1) | 14.2 (7.8–23.6) | −0.32 (−0.35 to −0.28) |
| East Asia | 6504.1 (3854–10604.6) | 0.8 (0.5–1.4) | 15440.1 (11998.6–20469.3) | 0.9 (0.7–1.1) | 0.25 (−0.12 to 0.61) |
| Eastern Europe | 6305.4 (5827.7–6849.3) | 2.9 (2.7–3.1) | 30863 (28266.2–34286.7) | 10.2 (9.4–11.3) | 4.79 (4.27–5.32) |
| Eastern Sub-Saharan Africa | 5022.3 (3043.8–5836) | 4.7 (3.3–5.6) | 8815.7 (5516–10938) | 4 (2.6–4.8) | −0.59 (−0.68 to −0.5) |
| High-income Asia Pacific | 9859.5 (8864.1–10400.9) | 5.7 (5.1–6.1) | 9501.1 (7713.2–10611) | 1.7 (1.5–1.9) | −3.28 (−3.92 to −2.64) |
| High-income North America | 25376.2 (23601.5–26588) | 7.3 (6.8–7.6) | 24247.2 (21349.1–26026.7) | 3.7 (3.3–4) | −2.84 (−3.06 to −2.61) |
| North Africa and Middle East | 6642.8 (4775.4–8970.1) | 2.9 (2.1–4.8) | 8609.5 (6979.8–12268.5) | 2 (1.6–2.9) | −1.27 (−1.33 to −1.22) |
| Oceania | 148.1 (93.3–210.5) | 4.1 (2.7–6) | 374.9 (246.5–543.2) | 4.1 (2.8–6.1) | 0.08 (0.04–0.12) |
| South Asia | 26628.3 (13854.7–37657.6) | 4.7 (2.4–6.8) | 66307.5 (52241.6–83817.3) | 4.9 (3.9–6.2) | 0.38 (0.26–0.5) |
| Southeast Asia | 8080.2 (6180.3–9669.3) | 3.9 (3–4.7) | 16778.2 (14362.5–19543.3) | 3.1 (2.7–3.6) | −0.99 (−1.18 to −0.8) |
| Southern Latin America | 6098.4 (5528.3–6686.1) | 14.3 (12.8–15.7) | 7254.7 (6537.2–7886.5) | 8.1 (7.4–8.8) | −1.83 (−2 to −1.65) |
| Southern Sub-Saharan Africa | 3751.7 (2947.9–4482.4) | 15.1 (11.4–18.4) | 6521.4 (5815.9–7299.2) | 12.7 (11.4–14.4) | −0.64 (−0.93 to −0.35) |
| Tropical Latin America | 11649.7 (11055.7–12137.2) | 14 (13.1–14.7) | 16167.2 (14667.6–17227.3) | 6.5 (5.9–6.9) | −3.05 (−3.34 to −2.76) |
| Western Europe | 50510 (44985.8–53731.8) | 8.8 (7.8–9.4) | 31015.5 (26582.1–33808.1) | 2.8 (2.4–3) | −4.05 (−4.46 to −3.64) |
| Western Sub-Saharan Africa | 11884.9 (8832.7–15893.1) | 13.7 (10.4–19.2) | 17463.6 (13283–20888) | 8.6 (6.9–10.2) | −1.77 (−1.91 to −1.64) |
