# Supplementary material for: Global, regional, and national burden of cardiomyopathy (including alcoholic cardiomyopathy and others) from 1990 to 2021: An analysis of data from the global burden of disease study 2021 and forecast to 2040
Source: PLoS One. 2026 Jan 30;21(1):e0341687. doi: 10.1371/journal.pone.0341687 (PMC12858021; doi:10.1371/journal.pone.0341687)
Supplement: S6 Table — (DOCX) [file pone.0341687.s017.docx]

**S6 Table.** **1990–2021 Global and regional DALYs trends in other cardiomyopathy burden.**

| location | Other Cardiomyopathy DALYs (95% UI) | | | | |
| --- | --- | --- | --- | --- | --- |
|  | Number_1990 | ASR per 100,000_1990 | Number_2021 | ASR per 100,000_2021 | EAPC_95% CI |
| Global | 5979925.1 (5132403.6–6835246) | 139.2 (120.6–154.8) | 8505291.2 (7759408–9352722.1) | 104.5 (95.2–114.9) | −0.97 (−1.04 to −0.89) |
| High SDI | 1557157.9 (1484755.3–1610413.5) | 154.7 (147.8–160.1) | 1264353.2 (1158896.4–1352845.7) | 72.7 (67.8–77.9) | −2.73 (−2.89 to −2.57) |
| High-middle SDI | 1128135.3 (1043535.2–1207022) | 132.2 (120.7–141.6) | 1965570.2 (1816162–2143007.3) | 116.2 (107.4–126) | −0.16 (−0.38 to 0.06) |
| Middle SDI | 1199142.5 (982382.1–1392222.1) | 93.5 (77.3–106.5) | 1829972.5 (1675057.3–1996857.8) | 72.8 (66.7–79.1) | −1.04 (−1.19 to −0.89) |
| Low-middle SDI | 1274134.5 (870758.9–1668305) | 145.9 (99.5–188.9) | 2075933.1 (1779506.3–2448279.3) | 130.7 (111.5–153.9) | −0.31 (−0.35 to −0.27) |
| Low SDI | 812383.2 (584506.6–1037707.2) | 213.3 (158.7–291.7) | 1359158.5 (1024614.7–1739474.7) | 178.2 (138–233.4) | −0.58 (−0.67 to −0.49) |
| Andean Latin America | 26363.2 (21482.6–32414.8) | 71.5 (60.1–83.2) | 23645.6 (19684–28433.2) | 38 (31.6–45.7) | −1.81 (−2.17 to −1.45) |
| Australasia | 33568.7 (31707.8–35553.9) | 155.2 (146.7–164.1) | 26332.7 (23728.7–28876.9) | 60 (54.2–66.2) | −2.63 (−3.38 to −1.87) |
| Caribbean | 42347.1 (32100.8–57146.6) | 132.4 (102.2–168.3) | 60269.2 (44498.3–77543.6) | 123.1 (88.9–162.4) | −0.11 (−0.39 to 0.16) |
| Central Asia | 65508.8 (56137.4–74755.6) | 115.6 (98.8–133.1) | 323986.2 (267626.4–383640.8) | 349.3 (290.9–410.9) | 4.76 (3.55–5.99) |
| Central Europe | 348746.9 (327995.5–374647.5) | 262.9 (247.1–281.8) | 386950.8 (349217.7–436336.9) | 186.3 (166.8–211.8) | −1.24 (−1.43 to −1.05) |
| Central Latin America | 84698.2 (80010.7–89577.8) | 63.7 (60.7–66.9) | 121353.8 (106346.9–139366.2) | 49.9 (43.6–57.6) | −1.05 (−1.17 to −0.93) |
| Central Sub-Saharan Africa | 157153.7 (99951.2–209219.4) | 417.1 (265.1–618.7) | 284819 (166003.8–434154.3) | 361.2 (205.1–573.9) | −0.44 (−0.48 to −0.4) |
| East Asia | 321515.9 (190060.1–474105.9) | 30.5 (18.2–45.4) | 405260.1 (327492.8–518641) | 24.6 (20–30.8) | −0.94 (−1.22 to −0.65) |
| Eastern Europe | 175189.1 (162085.8–192849.7) | 76.7 (71.2–84) | 950535.4 (876269.1–1060851.2) | 353.8 (326.8–392.1) | 5.39 (4.96–5.83) |
| Eastern Sub-Saharan Africa | 279920.5 (158945.6–335502.6) | 169.5 (109.1–196.2) | 442334.6 (277154.6–559257.5) | 142.4 (93.7–176) | −0.58 (−0.65 to −0.5) |
| High-income Asia Pacific | 232379.5 (217591.6–244483.7) | 129.9 (120.8–137.5) | 163453.9 (143427.7–178633) | 44.8 (41.1–49) | −3.03 (−3.47 to −2.59) |
| High-income North America | 658902.7 (628934.4–683585.7) | 205.8 (197.2–213.6) | 519971.1 (483929.7–549709.2) | 99.2 (93–105.2) | −2.96 (−3.18 to −2.73) |
| North Africa and Middle East | 386716.3 (259385.4–584768.7) | 110.3 (79.7–150.2) | 343604.2 (284191.1–442537.7) | 63.6 (52.3–84.6) | −1.71 (−1.77 to −1.64) |
| Oceania | 7333.1 (4428.1–10485.1) | 136.7 (86.5–193.4) | 18082.6 (11589.7–25234.3) | 145.6 (96–206.9) | 0.28 (0.24–0.32) |
| South Asia | 1021074.6 (522717.5–1385636.6) | 129.6 (68.3–180.7) | 1990422.7 (1554426.6–2495409) | 128 (100.3–160.3) | 0.1 (0.03–0.18) |
| Southeast Asia | 246594.6 (190148.5–295986.7) | 83.5 (64.2–99.6) | 461038.3 (382575.6–547568.4) | 71.8 (60.8–84.4) | −0.72 (−0.85 to −0.59) |
| Southern Latin America | 148076.7 (137616.8–161750.5) | 321.5 (297.4–351.1) | 149422.1 (139288.7–160280.6) | 180.1 (167.8–193) | −1.93 (−2.13 to −1.73) |
| Southern Sub-Saharan Africa | 112014.4 (97200.9–128793.7) | 341 (276–403.6) | 188247.4 (167875–210074.8) | 301.7 (269.4–336.9) | −0.44 (−0.64 to −0.23) |
| Tropical Latin America | 350637.5 (337521.4–365564.4) | 336.6 (322.4–350.5) | 439920.7 (414579–463834.8) | 177 (166.9–186.7) | −2.56 (−2.82 to −2.3) |
| Western Europe | 836842.5 (773770.2–878529.2) | 158.6 (147.5–166.5) | 507512.1 (455338.1–550912) | 60.2 (55.3–65.3) | −3.45 (−3.83 to −3.06) |
| Western Sub-Saharan Africa | 444341.2 (309817.5–556107.8) | 342.2 (253.2–459.1) | 698128.5 (500872.3–841876.9) | 223 (170.6–266) | −1.62 (−1.75 to −1.48) |
