# Supplementary material for: Global, regional, and national burden of cardiomyopathy (including alcoholic cardiomyopathy and others) from 1990 to 2021: An analysis of data from the global burden of disease study 2021 and forecast to 2040
Source: PLoS One. 2026 Jan 30;21(1):e0341687. doi: 10.1371/journal.pone.0341687 (PMC12858021; doi:10.1371/journal.pone.0341687)
Supplement: S7 Table — (DOCX) [file pone.0341687.s018.docx]

**S7 Table.** **1990–2021 Global and national prevalence trends in total cardiomyopathy burden.**

| location_name | Number_1990 | ASR per 100,000_1990 | Number_2021 | ASR per 100,000_2021 | Percentage change in the ASRs per 100,000 |
| --- | --- | --- | --- | --- | --- |
| Global | 2641735 (2242999–3040470) | 57.5 (49.1–65.9) | 4752361 (3937775–5566947) | 59.5 (49.4–69.6) | −14.9 (−22.9 to −6.9) |
| Andean Latin America | 10665 (8416–12915) | 30.2 (24.6–35.8) | 21898 (17313–26482) | 34.4 (27.4–41.5) | 25.1 (3.9–46.4) |
| Bolivia (Plurinational State of) | 1611 (1265–1958) | 26.6 (21.8–31.4) | 3982 (3064–4901) | 35.8 (28.1–43.4) | 65.4 (38.4–92.3) |
| Ecuador | 2820 (2221–3419) | 29 (23.7–34.3) | 5966 (4638–7294) | 34.1 (26.7–41.4) | 13.9 (−11.7 to 39.4) |
| Peru | 6234 (4872–7596) | 31.8 (25.9–37.6) | 11949 (9345–14554) | 34.2 (26.7–41.6) | 19.7 (−5.4 to 44.9) |
| Australasia | 24330 (20751–27908) | 120 (102.2–137.8) | 48267 (40623–55910) | 130.2 (108.4–152) | 95.6 (70.6–120.7) |
| Australia | 20941 (17770–24111) | 124.1 (105.6–142.5) | 42171 (35433–48909) | 134.8 (112–157.7) | 109.7 (80.2–139.2) |
| New Zealand | 3389 (2845–3933) | 100.3 (83.9–116.7) | 6096 (4952–7239) | 106.3 (86.5–126) | 25.5 (7.5–43.6) |
| Caribbean | 16649 (13750–19548) | 50.2 (41.7–58.6) | 35419 (30081–40758) | 72 (61–83) | 310.3 (277–343.5) |
| Antigua and Barbuda | 29 (23–34) | 47.9 (39.3–56.6) | 63 (52–74) | 69.3 (57.5–81.1) | 605.9 (508.9–702.8) |
| Bahamas | 162 (130–193) | 67.9 (56.7–79) | 289 (242–337) | 79.3 (66.3–92.4) | 171.8 (139.3–204.4) |
| Barbados | 239 (203–276) | 88.5 (75.6–101.4) | 425 (356–495) | 108.4 (92.2–124.6) | 97.4 (74.1–120.8) |
| Belize | 112 (89–136) | 63 (52.5–73.6) | 304 (252–357) | 79.5 (66.7–92.4) | 421.5 (354.2–488.7) |
| Bermuda | 46 (38–54) | 78.7 (65.2–92.2) | 128 (104–153) | 135.2 (113.7–156.6) | 680 (593.1–767) |
| Cuba | 4455 (3700–5209) | 42.4 (35.3–49.5) | 13911 (11799–16024) | 101.2 (86.4–115.9) | 780.8 (689.1–872.5) |
| Dominica | 73 (61–85) | 104.4 (88.4–120.4) | 85 (70–101) | 122.2 (102.2–142.2) | 46.3 (26.3–66.2) |
| Dominican Republic | 3258 (2578–3939) | 46.7 (38.4–55.1) | 6564 (5334–7793) | 61.6 (50.2–73) | 138.2 (110.3–166.1) |
| Grenada | 61 (50–72) | 71.5 (59.9–83.1) | 102 (86–118) | 98.2 (82.7–113.7) | 127.1 (101–153.2) |
| Guyana | 363 (287–439) | 48.6 (39.7–57.4) | 381 (318–444) | 54.5 (45.8–63.2) | 231.6 (192.3–270.9) |
| Haiti | 1819 (1500–2138) | 38.2 (32.2–44.1) | 5381 (4410–6353) | 51.2 (43.4–59.1) | 67.5 (47.6–87.3) |
| Jamaica | 1475 (1222–1729) | 64.3 (54.1–74.6) | 2123 (1776–2470) | 74.9 (62.9–86.9) | 74.6 (51.2–98) |
| Puerto Rico | 3102 (2549–3656) | 86 (71.1–100.9) | 2890 (2255–3526) | 61.7 (50–73.4) | 124.3 (94.9–153.8) |
| Saint Kitts and Nevis | 40 (33–48) | 96.2 (79.9–112.5) | 69 (58–80) | 122.2 (102.6–141.8) | 473.6 (410–537.2) |
| Saint Lucia | 103 (86–121) | 85.6 (72.5–98.6) | 229 (196–262) | 113.6 (97.7–129.5) | 345.2 (294.9–395.6) |
| Saint Vincent and the Grenadines | 31 (25–37) | 29.5 (24.6–34.3) | 53 (45–61) | 44.2 (37.7–50.7) | 216.4 (177.9–254.9) |
| Suriname | 201 (163–240) | 55.4 (45.1–65.7) | 374 (308–441) | 65.9 (54.1–77.7) | 138.7 (110.2–167.1) |
| Trinidad and Tobago | 432 (350–514) | 37.8 (31.3–44.3) | 747 (615–878) | 48.5 (40.5–56.6) | 218 (180.4–255.6) |
| United States Virgin Islands | 82 (69–96) | 83.7 (70.3–97.1) | 100 (81–119) | 91 (76.7–105.2) | 81.7 (62.9–100.5) |
| Central Asia | 20809 (17131–24487) | 31.9 (26.9–36.9) | 56643 (47217–66069) | 63.3 (52.7–73.9) | 145.9 (121.1–170.8) |
| Armenia | 1765 (1509–2021) | 55.4 (47.8–63.1) | 1679 (1423–1935) | 51.6 (43.2–60) | −15.1 (−35.2 to 5) |
| Azerbaijan | 5132 (4147–6117) | 74.6 (61.7–87.5) | 9713 (7854–11573) | 95 (78.3–111.7) | 21.7 (5.3–38.2) |
| Georgia | 1734 (1434–2034) | 31.3 (26–36.6) | 1627 (1248–2007) | 40.4 (32.5–48.2) | 31.6 (9.6–53.5) |
| Kazakhstan | 4298 (3268–5327) | 26.9 (20.6–33.2) | 24724 (19654–29794) | 138.6 (110.2–166.9) | 1022.1 (844.3–1200) |
| Kyrgyzstan | 918 (756–1080) | 24.6 (20.1–29.1) | 3698 (3060–4336) | 58.4 (48.7–68.2) | 345.6 (295.6–395.7) |
| Mongolia | 992 (831–1154) | 58.8 (49.9–67.8) | 2519 (2122–2915) | 86.6 (71.9–101.4) | 82.3 (57.2–107.4) |
| Tajikistan | 781 (589–974) | 14.1 (11.2–17) | 1971 (1460–2483) | 19.3 (14.8–23.9) | 65.2 (43.8–86.6) |
| Turkmenistan | 2893 (2349–3438) | 87.4 (72.5–102.3) | 4207 (3440–4974) | 86.2 (70.8–101.6) | −21.1 (−34.1 to −8) |
| Uzbekistan | 2295 (1757–2833) | 10.5 (8.4–12.7) | 6503 (5127–7879) | 19.7 (15.8–23.7) | 79 (53.6–104.3) |
| Central Europe | 119930 (100632–139229) | 95.1 (80–110.2) | 197757 (160987–234526) | 118.1 (99.7–136.6) | 77.5 (60.6–94.4) |
| Albania | 1898 (1553–2243) | 75.3 (60.2–90.5) | 2968 (2304–3631) | 92.7 (73.8–111.5) | 57.1 (34.5–79.7) |
| Bosnia and Herzegovina | 4424 (3698–5151) | 114.2 (94.3–134) | 5848 (4558–7137) | 141 (114.9–167.1) | 45.8 (26–65.5) |
| Bulgaria | 1257 (992–1523) | 14.5 (11.5–17.5) | 1637 (1206–2068) | 19.9 (15.8–24.1) | 60.8 (38.8–82.7) |
| Croatia | 1848 (1530–2167) | 37.9 (31.4–44.3) | 3265 (2552–3979) | 50.1 (41.8–58.5) | 90.4 (57–123.8) |
| Czechia | 3570 (2860–4281) | 33.8 (27.5–40.1) | 8750 (6702–10799) | 61.3 (49.8–72.9) | 386 (326.6–445.5) |
| Hungary | 8713 (7214–10212) | 75.3 (63.4–87.2) | 12031 (9709–14353) | 93.3 (77.8–108.8) | 49.6 (27.8–71.3) |
| Montenegro | 1108 (940–1277) | 187 (157.3–216.7) | 1334 (1084–1585) | 188.6 (156.4–220.8) | −5.4 (−19.9 to 9.1) |
| North Macedonia | 1996 (1673–2319) | 109.8 (91.5–128.1) | 3120 (2522–3718) | 136.5 (112.8–160.1) | 33.8 (15.3–52.3) |
| Poland | 57467 (47581–67352) | 147.9 (123.6–172.2) | 107646 (86815–128476) | 182.5 (151.3–213.7) | 91 (65–116.9) |
| Romania | 21622 (16647–26598) | 92.6 (71.6–113.6) | 27402 (19602–35202) | 98.3 (77.3–119.3) | 18.1 (−1.8–38) |
| Serbia | 7592 (6262–8921) | 83.4 (67.4–99.4) | 11568 (9000–14135) | 107.3 (89.1–125.6) | 62.4 (41.5–83.3) |
| Slovakia | 1989 (1654–2324) | 38 (31.4–44.6) | 4225 (3437–5012) | 65.7 (54.1–77.4) | 220.9 (181.6–260.3) |
| Slovenia | 4527 (3371–5682) | 207 (159.5–254.4) | 5084 (3776–6392) | 159.4 (130.1–188.6) | −6.5 (−23.9 to 11) |
| Central Latin America | 60432 (48626–72238) | 37.6 (31.4–43.7) | 103547 (83865–123228) | 42.2 (34.3–50.2) | 25.1 (13.6–36.7) |
| Colombia | 14279 (11101–17457) | 45.9 (37.2–54.7) | 26789 (20985–32593) | 55.1 (43.4–66.9) | 199.1 (164.6–233.6) |
| Costa Rica | 2088 (1702–2474) | 75.9 (63.5–88.3) | 3617 (3029–4206) | 73.4 (61.9–84.9) | 21.2 (5.6–36.9) |
| El Salvador | 1849 (1431–2267) | 35.8 (28.9–42.7) | 2426 (1926–2927) | 37.4 (29.8–45) | −6.5 (−22.4 to 9.4) |
| Guatemala | 3766 (2860–4672) | 42 (34.2–49.8) | 6237 (4768–7705) | 41.3 (32.4–50.3) | −12.6 (−30.8 to 5.7) |
| Honduras | 1576 (1241–1912) | 36 (29.7–42.2) | 4282 (3225–5338) | 43.5 (34.2–52.8) | 6.8 (−9.2 to 22.7) |
| Mexico | 22248 (17082–27414) | 25.2 (20.1–30.3) | 41117 (31806–50429) | 33 (25.5–40.5) | 55.8 (40.8–70.8) |
| Nicaragua | 1598 (1234–1963) | 42.5 (34.9–50.1) | 2817 (2264–3371) | 44.9 (36.6–53.1) | −4.5 (−18.7 to 9.7) |
| Panama | 1676 (1389–1964) | 79.5 (66.8–92.2) | 3897 (3245–4549) | 89.6 (74.7–104.5) | 14.4 (−1.6 to 30.4) |
| Venezuela (Bolivarian Republic of) | 11351 (9035–13668) | 63.4 (52.6–74.2) | 12365 (10277–14452) | 48.1 (39.7–56.4) | −56.6 (−68.3 to −44.9) |
| Central Sub-Saharan Africa | 31054 (21950–40159) | 72.5 (52.2–92.8) | 87714 (61109–114318) | 80.2 (58.2–102.3) | −1.5 (−12.9 to 10) |
| Angola | 5690 (3959–7422) | 71.7 (51–92.4) | 20625 (14327–26924) | 81.4 (58.9–103.9) | −6.1 (−20.3 to 8.1) |
| Central African Republic | 1372 (972–1773) | 63.5 (45.5–81.5) | 3037 (2095–3978) | 68.7 (49.1–88.4) | −5.2 (−19.4 to 8.9) |
| Congo | 1418 (992–1843) | 73.3 (52.7–93.9) | 3847 (2669–5026) | 82.7 (60.4–105.1) | −8.6 (−21.8 to 4.6) |
| Democratic Republic of the Congo | 21669 (15220–28119) | 73.2 (52.9–93.4) | 57799 (40155–75442) | 80.2 (57.9–102.5) | 1.7 (−12.6 to 15.9) |
| Equatorial Guinea | 239 (166–312) | 70.3 (51–89.5) | 999 (700–1299) | 84.8 (60.8–108.8) | −27.6 (−40.8 to −14.4) |
| Gabon | 666 (474–858) | 79.6 (57–102.3) | 1406 (982–1831) | 88.1 (63.3–112.9) | −35.9 (−47.8 to −24.1) |
| East Asia | 106692 (84299–129085) | 10 (8.1–11.9) | 300085 (236306–363864) | 19.7 (15.5–24) | 251 (221–281) |
| China | 96607 (75533–117680) | 9.4 (7.5–11.2) | 286201 (224994–347409) | 19.5 (15.3–23.7) | 303.4 (269.1–337.6) |
| Democratic People's Republic of Korea | 2693 (2143–3242) | 14.5 (11.6–17.3) | 4473 (3559–5388) | 18.1 (14.3–21.9) | 61 (42.1–80) |
| Taiwan (Province of China) | 7393 (6099–8687) | 41 (34–48) | 9410 (7498–11322) | 35.4 (28.8–42) | −23 (−36.9 to −9.1) |
| Eastern Europe | 219807 (190015–249598) | 94.8 (82.2–107.4) | 323184 (277537–368832) | 136 (116.9–155) | 102.3 (83.9–120.7) |
| Belarus | 8748 (7392–10104) | 80 (67.9–92.2) | 9288 (7733–10843) | 95 (79.5–110.6) | 44.9 (20.9–68.9) |
| Estonia | 1307 (1115–1499) | 79.1 (67.7–90.6) | 1340 (1116–1564) | 89.6 (74.4–104.9) | 22.8 (0–45.7) |
| Latvia | 2724 (2341–3107) | 96.8 (83.2–110.5) | 3991 (3322–4659) | 161.7 (139–184.4) | 132.1 (103.5–160.6) |
| Lithuania | 2406 (2047–2765) | 63.1 (53.8–72.4) | 3174 (2611–3738) | 96.3 (79.1–113.4) | 101.3 (70.4–132.1) |
| Republic of Moldova | 1522 (1291–1754) | 34.3 (29–39.6) | 2744 (2276–3212) | 70.2 (58.4–82) | 215.2 (176–254.4) |
| Russian Federation | 145303 (125560–165045) | 95.3 (82.6–108.1) | 247120 (211398–282842) | 147.7 (126.7–168.7) | 130.6 (105.9–155.2) |
| Ukraine | 57796 (49698–65894) | 104.2 (89.6–118.7) | 55528 (47525–63530) | 113.1 (96.2–130.1) | 23.6 (7.8–39.4) |
| Eastern Sub-Saharan Africa | 158254 (119410–197098) | 111.9 (84–139.8) | 402363 (307369–497357) | 126.7 (94.9–158.5) | 10.4 (3.5–17.4) |
| Burundi | 4038 (2912–5165) | 97 (70.4–123.6) | 10700 (7745–13656) | 110.2 (79.7–140.6) | 18.8 (2.1–35.5) |
| Comoros | 392 (279–506) | 111.1 (80.4–141.8) | 798 (593–1002) | 121.4 (88.7–154) | 0.6 (−12.6 to 13.8) |
| Djibouti | 337 (240–434) | 110.3 (79.9–140.7) | 1278 (947–1608) | 120.5 (88–153) | 4.1 (−11.4 to 19.6) |
| Eritrea | 2477 (1765–3188) | 97.4 (70.2–124.6) | 5708 (4106–7310) | 109.1 (79.7–138.6) | 10.4 (−5 to 25.8) |
| Ethiopia | 59754 (47133–72375) | 153.5 (118–188.9) | 151147 (120693–181600) | 182.9 (140.9–224.9) | 25.9 (16.9–34.9) |
| Kenya | 16680 (12477–20883) | 112 (82.4–141.6) | 44009 (33581–54436) | 117.8 (87.3–148.3) | −5.1 (−12.4 to 2.3) |
| Madagascar | 9484 (6942–12026) | 106.3 (76.8–135.8) | 25136 (18382–31890) | 115.8 (83.7–147.9) | 3.1 (−11.7 to 18) |
| Malawi | 6842 (4968–8716) | 95.1 (67.8–122.4) | 15490 (11362–19618) | 107.7 (78.5–136.8) | 8.5 (−6.8 to 23.8) |
| Mozambique | 7701 (5471–9931) | 77.1 (53.6–100.6) | 19054 (13552–24557) | 86 (61.2–110.7) | 4.6 (−10.9 to 20.1) |
| Rwanda | 5374 (3801–6948) | 100.8 (72–129.6) | 11838 (8651–15025) | 111.7 (81.1–142.4) | 9.8 (−5.2 to 24.8) |
| Somalia | 5187 (3705–6670) | 91.2 (67.2–115.1) | 14793 (10373–19213) | 95.1 (68.6–121.6) | −4.4 (−18 to 9.2) |
| South Sudan | 4514 (3242–5787) | 105.2 (75.1–135.4) | 7949 (5771–10126) | 110.4 (80.4–140.4) | −9.1 (−22.8 to 4.5) |
| Uganda | 9440 (6498–12382) | 77.1 (55.5–98.8) | 26370 (17870–34870) | 85.1 (60.3–110) | 5.1 (−10.8 to 21) |
| United Republic of Tanzania | 20383 (14795–25970) | 105.3 (76.5–134.1) | 51756 (37943–65568) | 114.3 (82.9–145.7) | −0.9 (−15.6 to 13.9) |
| Zambia | 5537 (3957–7116) | 96.4 (69.6–123.1) | 15989 (11371–20606) | 110.4 (78.5–142.3) | 13.4 (−2.6 to 29.3) |
| High-income Asia Pacific | 145218 (121835–168602) | 83.6 (69.2–98) | 207491 (166585–248397) | 84.3 (69.9–98.7) | −22.7 (−32.2 to −13.1) |
| Brunei Darussalam | 172 (138–206) | 70.6 (58.1–83) | 310 (251–369) | 75.5 (61.4–89.6) | −6.7 (−21.2 to 7.8) |
| Japan | 125757 (105035–146478) | 96.7 (80.4–112.9) | 159586 (125402–193770) | 88 (72.7–103.2) | −25.5 (−35.7 to −15.3) |
| Republic of Korea | 17851 (13909–21792) | 44.4 (35.5–53.4) | 45182 (36480–53884) | 80.4 (66.1–94.8) | 149 (115.2–182.7) |
| Singapore | 1439 (1156–1722) | 52.1 (43–61.2) | 2413 (1888–2939) | 38.7 (30.8–46.6) | −69 (−80.3 to −57.7) |
| High-income North America | 475379 (400686–550072) | 162.9 (137.7–188) | 579544 (483537–675552) | 135.4 (113.3–157.5) | −29.3 (−40.3 to −18.2) |
| Canada | 32378 (27490–37265) | 116.2 (98.3–134) | 53806 (44630–62982) | 109.8 (93.6–126) | 6.5 (−11.6 to 24.7) |
| Greenland | 74 (61–87) | 142.1 (118.5–165.7) | 86 (71–101) | 155.8 (127.9–183.6) | 73.3 (48.2–98.4) |
| United States of America | 442917 (371828–514005) | 167.8 (141.4–194.1) | 525644 (435793–615494) | 138 (114.8–161.2) | −33.2 (−45.4 to −20.9) |
| North Africa and Middle East | 108680 (85520–131840) | 30.5 (25.1–35.8) | 225006 (176441–273570) | 37.5 (29.9–45.1) | 28.8 (17.1–40.5) |
| Afghanistan | 1202 (930–1475) | 11.6 (9.4–13.7) | 5210 (3694–6725) | 15.8 (12.1–19.5) | 32.3 (11.9–52.7) |
| Algeria | 6898 (5156–8640) | 25.7 (20.5–30.8) | 14235 (10803–17667) | 33.4 (25.5–41.3) | 36.9 (18.4–55.4) |
| Bahrain | 338 (268–407) | 74.9 (62.1–87.7) | 1178 (950–1406) | 93.5 (77.4–109.6) | 39.6 (23.9–55.3) |
| Egypt | 13423 (10325–16520) | 22.2 (17.7–26.7) | 30949 (22686–39212) | 28.1 (21.6–34.6) | 53.6 (30.2–77.1) |
| Iran (Islamic Republic of) | 17176 (12935–21416) | 28 (22.3–33.7) | 28853 (21989–35717) | 37.2 (28.3–46.2) | 46 (32.6–59.5) |
| Iraq | 9375 (7293–11457) | 50.7 (42.3–59.1) | 24304 (19263–29346) | 60.7 (49.6–71.8) | 6.2 (−9 to 21.4) |
| Jordan | 605 (446–764) | 14.4 (11.4–17.3) | 1680 (1260–2099) | 14.2 (10.9–17.4) | −17.3 (−32.2 to −2.3) |
| Kuwait | 698 (540–855) | 42.7 (34.2–51.1) | 883 (691–1075) | 25.3 (19.7–30.8) | −93.7 (−103.3 to −84.1) |
| Lebanon | 655 (533–776) | 22.5 (18.8–26.2) | 1768 (1441–2095) | 33.2 (26.8–39.6) | 66.7 (45.7–87.7) |
| Libya | 1953 (1456–2449) | 38.5 (30.2–46.8) | 2082 (1601–2563) | 37.3 (28.3–46.4) | −38 (−52.2 to −23.9) |
| Morocco | 8805 (6603–11007) | 30.9 (24.1–37.6) | 13649 (10455–16844) | 39.2 (30–48.4) | 27.3 (7.4–47.3) |
| Oman | 1341 (1068–1615) | 70.6 (59.6–81.7) | 3060 (2409–3712) | 76.1 (60.6–91.5) | −11.6 (−28.2 to 5.1) |
| Palestine | 1054 (809–1299) | 47 (38.7–55.4) | 2997 (2315–3678) | 58.3 (47.2–69.4) | 31.1 (14.9–47.3) |
| Qatar | 292 (235–348) | 76.8 (63.7–90) | 1852 (1429–2274) | 82.6 (67.4–97.9) | 73.5 (51.6–95.3) |
| Saudi Arabia | 9552 (7471–11633) | 61.9 (51.1–72.8) | 18927 (15212–22642) | 65.5 (53.4–77.6) | −19.7 (−32.8 to −6.6) |
| Sudan | 3397 (2542–4252) | 16.3 (13.1–19.5) | 9966 (7166–12765) | 21.9 (16.5–27.3) | 18.5 (−3.1 to 40) |
| Syrian Arab Republic | 3933 (2879–4987) | 25.4 (20–30.7) | 4591 (3450–5733) | 34.3 (26.1–42.4) | 34.1 (12.6–55.5) |
| Tunisia | 2687 (2054–3319) | 31.2 (24.8–37.6) | 4075 (3202–4947) | 36.9 (28.4–45.3) | 18.4 (1.5–35.4) |
| Turkey | 21489 (16638–26339) | 36.9 (30–43.9) | 38608 (30092–47125) | 49.1 (37.8–60.4) | 69 (44.7–93.2) |
| United Arab Emirates | 1533 (1241–1824) | 92.4 (77.7–107.2) | 8028 (6106–9950) | 104.7 (86.8–122.6) | 6.6 (−9.3 to 22.6) |
| Yemen | 2218 (1630–2805) | 15.4 (12.6–18.2) | 7902 (5718–10086) | 22.4 (17.1–27.6) | 34.7 (14.1–55.4) |
| Oceania | 1051 (807–1294) | 20.9 (16.6–25.3) | 2707 (2047–3366) | 24.2 (18.4–29.9) | 3 (−8.6 to 14.6) |
| American Samoa | 25 (19–30) | 58.7 (46.8–70.5) | 27 (21–33) | 58.9 (45.9–72) | −51.9 (−63.6 to −40.3) |
| Cook Islands | 1 (1–2) | 6.7 (4.9–8.5) | 1 (1–2) | 8.3 (5.7–10.9) | 13.4 (−11 to 37.7) |
| Fiji | 97 (73–121) | 15.2 (11.9–18.5) | 159 (121–197) | 19.2 (14.6–23.7) | 42.1 (24.9–59.4) |
| Guam | 49 (38–61) | 40.1 (32–48.2) | 65 (50–79) | 40.8 (31.4–50.2) | −19.7 (−32.7 to −6.6) |
| Kiribati | 9 (7–11) | 17.7 (13.9–21.4) | 17 (13–21) | 19.4 (14.9–23.9) | 5.8 (−10.9 to 22.6) |
| Marshall Islands | 8 (6–10) | 20.7 (16.2–25.1) | 11 (8–14) | 23.1 (17.6–28.5) | −4.6 (−17.4 to 8.3) |
| Micronesia (Federated States of) | 20 (15–24) | 21.9 (17.3–26.6) | 22 (16–28) | 25.2 (19.4–31.1) | 3.1 (−11.4 to 17.7) |
| Nauru | 2 (1–2) | 21.1 (16.4–25.8) | 2 (2–3) | 24.1 (18.2–29.9) | 3.2 (−12.8 to 19.2) |
| Niue | 1 (1–1) | 27.6 (21.9–33.2) | 0 (0–1) | 29.2 (22.5–36) | −13.3 (−25.9 to −0.7) |
| Northern Mariana Islands | 16 (12–19) | 46.9 (37.2–56.6) | 21 (16–26) | 48.3 (37.9–58.7) | −47.9 (−60 to −35.8) |
| Palau | 3 (2–4) | 22.6 (17.8–27.4) | 4 (3–5) | 24.9 (18.9–30.8) | −12.3 (−25 to 0.3) |
| Papua New Guinea | 607 (467–747) | 20.5 (16–25) | 1981 (1482–2480) | 24.6 (18.6–30.6) | 10.2 (−4.3 to 24.8) |
| Samoa | 43 (33–53) | 28.1 (22.2–34.1) | 62 (47–77) | 30.7 (23.5–38) | −12.4 (−26.4 to 1.5) |
| Solomon Islands | 43 (32–54) | 16.3 (12.7–19.9) | 108 (80–136) | 19 (14.6–23.4) | 7.6 (−8 to 23.2) |
| Tokelau | 0 (0–1) | 29.4 (23.4–35.3) | 0 (0–1) | 35.4 (27.5–43.4) | 9.7 (−6 to 25.4) |
| Tonga | 33 (25–40) | 36.7 (29.2–44.1) | 40 (31–49) | 38.4 (29.8–46.9) | −19.1 (−32.6 to −5.6) |
| Tuvalu | 2 (1–2) | 19.6 (15.6–23.6) | 3 (2–4) | 25.1 (19.2–31) | 21.4 (4.9–37.8) |
| Vanuatu | 27 (20–33) | 19.8 (15.5–24.2) | 61 (45–77) | 21.8 (16.9–26.7) | −10.5 (−23.8 to 2.8) |
| South Asia | 279720 (228209–331231) | 34 (28.1–39.8) | 718523 (573393–863653) | 44.8 (36.3–53.4) | 36.7 (23.3–50.2) |
| Bangladesh | 24581 (20244–28917) | 36.9 (29.5–44.2) | 72564 (57884–87244) | 50.4 (40.7–60.1) | 50.2 (28.8–71.6) |
| Bhutan | 130 (106–154) | 34 (27.7–40.3) | 357 (296–418) | 53.3 (44–62.5) | 82.1 (59.5–104.7) |
| India | 218921 (177159–260683) | 33.2 (27.2–39.2) | 552322 (432155–672489) | 44.4 (35.5–53.2) | 38.7 (24.2–53.1) |
| Nepal | 4717 (3850–5585) | 33.4 (27.6–39.2) | 12879 (10413–15346) | 47.6 (39.2–56) | 43.6 (23.4–63.7) |
| Pakistan | 31371 (24859–37883) | 35.9 (29.4–42.5) | 80401 (61981–98821) | 42.5 (33.4–51.7) | 13.8 (−1.6 to 29.1) |
| Southeast Asia | 73092 (58988–87195) | 23.5 (19.2–27.8) | 164367 (130971–197764) | 26.8 (21.5–32.2) | 36.8 (25–48.6) |
| Cambodia | 666 (535–796) | 13.7 (10.4–16.9) | 2146 (1675–2618) | 17.2 (13.4–21.1) | 77.5 (53.1–101.8) |
| Indonesia | 26273 (20709–31836) | 22.5 (17.7–27.3) | 57274 (44410–70138) | 26.3 (20.3–32.2) | 24.3 (10–38.6) |
| Lao People's Democratic Republic | 279 (220–338) | 13.1 (10.1–16.1) | 917 (711–1123) | 17.5 (13.4–21.7) | 78.1 (52.8–103.5) |
| Malaysia | 6338 (5201–7474) | 45.3 (37.2–53.3) | 11515 (9326–13703) | 41.6 (33.7–49.5) | −44.1 (−57.1 to −31.2) |
| Maldives | 25 (20–30) | 20.3 (16–24.6) | 130 (105–156) | 33.5 (27–40) | 134.5 (109.1–160) |
| Mauritius | 120 (96–144) | 14 (11.2–16.8) | 477 (375–578) | 34.5 (27.6–41.4) | 150.9 (114–187.8) |
| Myanmar | 3594 (2880–4308) | 15.6 (12–19.1) | 9801 (7644–11957) | 20.9 (16.1–25.7) | 81.4 (55.2–107.7) |
| Philippines | 11639 (9079–14199) | 29.2 (23–35.4) | 27165 (20908–33422) | 29.7 (22.9–36.5) | −4.6 (−16.2 to 6.9) |
| Seychelles | 30 (25–35) | 44.6 (37.1–52.1) | 38 (31–45) | 37.7 (30.4–44.9) | 36.9 (15.8–58) |
| Sri Lanka | 6950 (5787–8112) | 54.3 (45–63.6) | 7933 (6312–9554) | 34.7 (27.9–41.6) | −64.5 (−77.3 to −51.7) |
| Thailand | 2921 (2345–3496) | 6.7 (5.5–8) | 13038 (9953–16123) | 15.2 (11.8–18.5) | 284.3 (242.8–325.8) |
| Timor-Leste | 73 (59–86) | 19 (15–23) | 232 (179–285) | 22.4 (17.3–27.4) | 29.2 (9.3–49) |
| Viet Nam | 14080 (11376–16784) | 28.5 (22.6–34.5) | 33474 (26833–40115) | 37 (29.7–44.4) | 80.7 (57.5–104) |
| Southern Latin America | 49050 (40705–57395) | 102.8 (84.6–121) | 73399 (58930–87869) | 99.7 (81.3–118.2) | −62.2 (−72.5 to −51.9) |
| Uruguay | 3313 (2746–3879) | 98.2 (82–114.4) | 3285 (2612–3959) | 79.8 (64.4–95.1) | −58.3 (−69 to −47.6) |
| Argentina | 35542 (29104–41981) | 110.2 (89.8–130.5) | 55847 (44519–67176) | 113.2 (91.7–134.7) | −67.5 (−80.3 to −54.7) |
| Chile | 10193 (8273–12113) | 81.7 (67.1–96.4) | 14263 (11472–17054) | 72.9 (58.9–86.8) | −34.9 (−46.7 to −23.1) |
| Southern Sub-Saharan Africa | 33160 (24481–41839) | 75.4 (56.6–94.2) | 59625 (44508–74742) | 79.9 (59.6–100.2) | −17.1 (−24 to −10.2) |
| Botswana | 881 (624–1137) | 84.5 (61.4–107.6) | 1918 (1412–2424) | 88.9 (65–112.8) | −9.8 (−24.5 to 4.9) |
| Eswatini | 497 (344–649) | 81 (58.5–103.5) | 826 (607–1044) | 85.8 (62.1–109.5) | −17.2 (−32.4 to −1.9) |
| Lesotho | 965 (687–1242) | 75.1 (55.5–94.7) | 1289 (939–1639) | 80.3 (58.2–102.4) | 0.5 (−15.6 to 16.7) |
| Namibia | 982 (704–1259) | 86.9 (63.3–110.5) | 1969 (1446–2493) | 93.6 (69–118.3) | −5.4 (−19.8 to 9) |
| South Africa | 21521 (15686–27355) | 67.4 (49–85.8) | 40895 (29974–51816) | 74.6 (55–94.2) | −21.5 (−27.9 to −15.1) |
| Zimbabwe | 8316 (6472–10159) | 108.5 (83.2–133.8) | 12728 (9537–15919) | 103.5 (76.6–130.3) | −12 (−29.5 to 5.4) |
| Tropical Latin America | 144440 (121194–167686) | 121 (101.1–141) | 284686 (233197–336175) | 122.7 (99.9–145.5) | −31.6 (−40.7 to −22.4) |
| Brazil | 141884 (118919–164849) | 122.4 (102–142.7) | 280435 (229632–331237) | 124.4 (101.3–147.5) | −31.1 (−40.3 to −22) |
| Paraguay | 2556 (2060–3053) | 74.7 (61.4–87.9) | 4252 (3389–5114) | 65 (51.4–78.5) | −41.8 (−54.8 to −28.7) |
| Western Europe | 465078 (389851–540304) | 105.9 (90.9–120.8) | 594168 (485270–703066) | 96.5 (82–111.1) | −12.2 (−22.7 to −1.8) |
| Andorra | 54 (45–62) | 109 (91.7–126.4) | 121 (98–143) | 113.7 (95.5–132) | 3.3 (−11.8 to 18.3) |
| Austria | 25468 (21296–29641) | 253 (219.4–286.6) | 15625 (12781–18469) | 129.1 (108.8–149.3) | −103.9 (−114.8 to −93) |
| Belgium | 9122 (7353–10890) | 82.2 (67.6–96.7) | 8729 (6904–10554) | 54.5 (45.1–63.8) | 3.2 (−20.2 to 26.7) |
| Cyprus | 306 (250–363) | 41.2 (33.6–48.8) | 660 (526–794) | 47.8 (38.6–56.9) | 23.9 (5.4–42.3) |
| Denmark | 1722 (1462–1982) | 33.6 (28–39.1) | 2431 (1950–2912) | 30.9 (25.6–36.1) | 17.6 (−6 to 41.1) |
| Finland | 3824 (3289–4358) | 77.4 (65.6–89.2) | 5672 (4626–6718) | 70.1 (59.4–80.9) | −4.9 (−25.3 to 15.5) |
| France | 124252 (101813–146691) | 179.7 (150.7–208.7) | 175534 (133756–217311) | 168.5 (138.4–198.6) | 63.3 (37.1–89.6) |
| Germany | 88011 (75430–100591) | 101.2 (86.6–115.8) | 110506 (90438–130574) | 96.7 (81.2–112.3) | −19.5 (−33.5 to −5.4) |
| Greece | 5353 (4372–6333) | 49.1 (40.4–57.9) | 5006 (4098–5914) | 43.7 (35.6–51.9) | −21.2 (−37.5 to −4.9) |
| Iceland | 76 (63–89) | 30.4 (24.7–36.1) | 81 (64–97) | 20.1 (16.1–24.1) | −31.9 (−49.3 to −14.5) |
| Ireland | 3463 (2880–4046) | 96.1 (80.1–112) | 4828 (3918–5738) | 80.4 (66.6–94.3) | 11.8 (−6.6 to 30.1) |
| Israel | 2161 (1725–2597) | 43.1 (34.8–51.4) | 3504 (2827–4181) | 33.1 (26.4–39.8) | 38.5 (15.6–61.4) |
| Italy | 90769 (66280–115258) | 127.5 (100.5–154.4) | 90324 (65936–114712) | 87.8 (69.6–106) | −0.4 (−16.7 to 15.9) |
| Luxembourg | 375 (307–442) | 99.6 (82.4–116.8) | 490 (392–589) | 64.1 (52.1–76) | −31.7 (−47 to −16.4) |
| Malta | 330 (275–385) | 93.1 (77.3–109) | 270 (209–331) | 43 (35–51) | −66 (−78 to −54) |
| Monaco | 64 (51–76) | 128.8 (109.6–148.1) | 74 (58–90) | 114 (96–131.9) | −24 (−36.6 to −11.3) |
| Netherlands | 13863 (11835–15891) | 87.2 (74.4–100) | 17955 (14427–21484) | 76.8 (64.4–89.2) | −4.1 (−23.3 to 15.1) |
| Norway | 2446 (2034–2858) | 65.4 (53.3–77.5) | 4693 (3704–5681) | 82.1 (65.6–98.6) | 28.4 (10.6–46.2) |
| Portugal | 4750 (3939–5561) | 46.7 (38.8–54.5) | 7940 (6041–9840) | 47.7 (38.6–56.8) | 39.2 (14.9–63.5) |
| San Marino | 34 (28–40) | 123.6 (105.3–141.9) | 56 (43–70) | 103.8 (86.5–121.2) | −3.4 (−18 to 11.2) |
| Spain | 41850 (33774–49927) | 97.2 (81–113.3) | 68932 (56009–81854) | 103 (86.7–119.2) | 40.7 (8.2–73.3) |
| Sweden | 14553 (12341–16765) | 172.4 (145–199.9) | 19796 (15928–23663) | 159.7 (132–187.3) | 52.7 (29.1–76.4) |
| Switzerland | 3768 (3151–4386) | 52.2 (43.5–61) | 4766 (3874–5658) | 40.1 (33.4–46.8) | −26.1 (−41.4 to −10.9) |
| United Kingdom | 28083 (23766–32399) | 51.5 (42.5–60.5) | 45653 (37871–53435) | 64.3 (53–75.6) | 59.9 (47–72.9) |
| Western Sub-Saharan Africa | 98244 (72558–123931) | 67.5 (50.2–84.8) | 265969 (191434–340504) | 71.5 (52.3–90.8) | 12.4 (6.5–18.3) |
| Benin | 1910 (1331–2488) | 54.2 (37.9–70.6) | 5891 (4048–7733) | 58.4 (40.7–76.1) | 27.9 (11.9–43.9) |
| Burkina Faso | 3715 (2548–4881) | 53.1 (36.5–69.7) | 9560 (6573–12546) | 56.5 (39.1–73.9) | 31.6 (14.9–48.4) |
| Cabo Verde | 176 (124–229) | 62.6 (44.3–80.9) | 381 (268–493) | 70.5 (49.9–91.1) | 27.8 (13.7–41.8) |
| Cameroon | 4310 (2919–5702) | 53.8 (37.9–69.7) | 14947 (10123–19770) | 60.6 (42.6–78.7) | 26.9 (11.2–42.5) |
| Chad | 2230 (1553–2908) | 50 (35.2–64.9) | 6655 (4530–8780) | 54.1 (37.9–70.3) | 32.5 (16.9–48.1) |
| Côte d'Ivoire | 4838 (3317–6359) | 55.3 (38.7–72) | 12877 (8840–16914) | 59.3 (42.4–76.2) | 24.2 (8.2–40.2) |
| Gambia | 405 (279–532) | 56.3 (39.5–73.2) | 1111 (770–1452) | 60 (42.3–77.8) | 18.2 (3.1–33.2) |
| Ghana | 6900 (5304–8496) | 63.3 (47.7–78.9) | 18895 (13362–24428) | 68.5 (48.2–88.7) | 8.6 (−13.8 to 31) |
| Guinea | 2443 (1701–3185) | 52.1 (36.8–67.5) | 5584 (3836–7332) | 55.8 (39.5–72.1) | 20.8 (3.7–37.9) |
| Guinea-Bissau | 364 (251–478) | 49.7 (35–64.3) | 831 (568–1093) | 53.8 (38.1–69.5) | 25.7 (10–41.4) |
| Liberia | 1024 (714–1335) | 55.7 (38.5–72.9) | 2548 (1777–3319) | 59.9 (42–77.9) | 22.4 (6.8–37.9) |
| Mali | 3302 (2307–4296) | 51 (35.7–66.4) | 9478 (6476–12481) | 55.1 (38.9–71.3) | 25.1 (8.9–41.3) |
| Mauritania | 933 (634–1233) | 58.5 (41–76.1) | 2217 (1528–2906) | 63.1 (43.8–82.5) | 24 (7–40.9) |
| Niger | 2951 (1996–3907) | 53.3 (37.3–69.3) | 9408 (6387–12429) | 55.2 (38.4–72.1) | 13.7 (−1 to 28.4) |
| Nigeria | 57488 (42948–72027) | 81.9 (61.1–102.8) | 152570 (112544–192597) | 86.7 (64.4–109) | 4.4 (−1 to 9.9) |
| Sao Tome and Principe | 59 (41–77) | 63.2 (44.8–81.5) | 125 (86–164) | 68.5 (48.6–88.4) | 16.6 (0–33.1) |
| Senegal | 2025 (1362–2687) | 37 (25.3–48.7) | 4933 (3389–6476) | 39.7 (27.1–52.3) | 20.9 (4.9–36.9) |
| Sierra Leone | 1755 (1210–2299) | 55.2 (38.3–72.2) | 4006 (2789–5224) | 59.2 (42.4–75.9) | 24.9 (9.3–40.5) |
| Togo | 1412 (961–1863) | 54 (38–70) | 3950 (2732–5168) | 57.9 (40.7–75.2) | 24 (9–39) |
