# Supplementary material for: Global, regional, and national burden of cardiomyopathy (including alcoholic cardiomyopathy and others) from 1990 to 2021: An analysis of data from the global burden of disease study 2021 and forecast to 2040
Source: PLoS One. 2026 Jan 30;21(1):e0341687. doi: 10.1371/journal.pone.0341687 (PMC12858021; doi:10.1371/journal.pone.0341687)
Supplement: S8 Table — (DOCX) [file pone.0341687.s019.docx]

**S8 Table.** **1990–2021 Global and national mortality trends in total cardiomyopathy burden.**

| location_name | Number_1990 | ASR per 100,000_1990 | Number_2021 | ASR per 100,000_2021 | Percentage change in the ASRs per 100,000 |
| --- | --- | --- | --- | --- | --- |
| Global | 254222 (230367–278077) | 7.3 (6.6–7.9) | 370274 (340966–399581) | 4.5 (4.1–4.8) | −75.5 (−83.4 to −67.7) |
| Andean Latin America | 519 (443–595) | 2 (1.7–2.3) | 645 (522–767) | 1.1 (0.9–1.3) | −102.4 (−129.2 to −75.5) |
| Bolivia (Plurinational State of) | 122 (77–167) | 2.8 (1.9–3.7) | 170 (120–220) | 1.9 (1.4–2.5) | −64.2 (−136 to 7.6) |
| Ecuador | 114 (102–127) | 1.9 (1.7–2.1) | 216 (160–271) | 1.4 (1–1.7) | −102.4 (−128.5 to −76.3) |
| Peru | 283 (232–333) | 1.9 (1.5–2.3) | 259 (178–341) | 0.8 (0.5–1) | −113.7 (−157 to −70.3) |
| Australasia | 1586 (1493–1679) | 7.2 (6.8–7.6) | 1657 (1507–1806) | 3.1 (2.8–3.3) | −90.9 (−100 to −81.8) |
| Australia | 1332 (1251–1414) | 7.3 (6.9–7.8) | 1340 (1208–1472) | 2.9 (2.7–3.2) | −91.1 (−100.2 to −82.1) |
| New Zealand | 253 (229–278) | 6.8 (6.2–7.5) | 317 (292–341) | 3.9 (3.6–4.2) | −81.1 (−98.1 to −64.1) |
| Caribbean | 1232 (1011–1453) | 4.5 (3.7–5.3) | 2951 (2516–3387) | 5.6 (4.7–6.5) | 219.8 (134.6–304.9) |
| Antigua and Barbuda | 2 (2–2) | 4 (3.6–4.4) | 6 (6–7) | 6.4 (5.9–6.9) | 642.7 (459.4–826) |
| Bahamas | 13 (12–15) | 7.9 (7–8.7) | 41 (34–47) | 10.1 (8.6–11.7) | 171.6 (67.2–276) |
| Barbados | 20 (18–22) | 6.8 (6.2–7.5) | 34 (29–40) | 6.9 (5.8–8.1) | 30.4 (−19.3 to 80.1) |
| Belize | 5 (4–6) | 4.2 (3.5–4.9) | 19 (17–21) | 6.2 (5.6–6.9) | 530.7 (260.4–801) |
| Bermuda | 3 (3–4) | 5.6 (4.7–6.6) | 9 (8–11) | 6.6 (5.7–7.6) | 498.3 (206.7–789.9) |
| Cuba | 232 (209–255) | 2.3 (2.1–2.5) | 1150 (1028–1272) | 6 (5.4–6.6) | 815.4 (631.4–999.5) |
| Dominica | 9 (7–11) | 14.9 (11.6–18.1) | 14 (11–17) | 17.3 (13.9–20.8) | 43.8 (−47.1 to 134.7) |
| Dominican Republic | 156 (123–189) | 3.2 (2.2–4.1) | 339 (254–425) | 3.3 (2.5–4.2) | 113 (−56.7 to 282.7) |
| Grenada | 6 (6–7) | 8.4 (7.6–9.2) | 14 (12–15) | 13 (11.7–14.3) | 141.4 (84.8–197.9) |
| Guyana | 38 (35–42) | 9 (8–9.9) | 67 (53–80) | 10.5 (8.5–12.5) | 140.4 (36.1–244.6) |
| Haiti | 347 (170–524) | 9.5 (4.7–14.2) | 706 (373–1039) | 8.9 (4.7–13.1) | −4.7 (−72.3 to 62.9) |
| Jamaica | 83 (73–93) | 4.4 (3.9–5) | 158 (127–189) | 5 (4–6) | 94.5 (13.9–175.2) |
| Puerto Rico | 202 (188–216) | 5.8 (5.4–6.2) | 145 (124–167) | 2.1 (1.8–2.3) | 48 (−7.1 to 103) |
| Saint Kitts and Nevis | 4 (4–4) | 11.7 (10.9–12.5) | 8 (6–9) | 12.5 (10.8–14.2) | 387.2 (216.2–558.2) |
| Saint Lucia | 10 (9–11) | 11.9 (10.9–12.9) | 28 (25–32) | 12.2 (10.6–13.8) | 243 (129.8–356.1) |
| Saint Vincent and the Grenadines | 2 (2–2) | 3.1 (2.8–3.3) | 7 (6–8) | 5.1 (4.6–5.6) | 274.3 (201.7–346.9) |
| Suriname | 13 (11–16) | 4.8 (3.8–5.9) | 27 (20–34) | 4.5 (3.3–5.6) | 68.2 (−68.8 to 205.3) |
| Trinidad and Tobago | 35 (32–39) | 4.4 (4–4.8) | 71 (57–85) | 3.9 (3.1–4.6) | 86.9 (−11.5 to 185.3) |
| United States Virgin Islands | 8 (6–10) | 9.5 (7.2–11.7) | 8 (6–11) | 5.7 (4.2–7.2) | −40.6 (−118.7 to 37.6) |
| Central Asia | 2194 (1912–2477) | 4.5 (3.9–5.1) | 10887 (9173–12602) | 13 (11.1–14.9) | 254.4 (168.5–340.2) |
| Armenia | 195 (158–231) | 7.5 (6.1–8.9) | 187 (149–226) | 4.6 (3.6–5.5) | −62.9 (−110.9 to −14.9) |
| Azerbaijan | 664 (443–884) | 13 (8.6–17.5) | 1412 (721–2103) | 13.4 (7.3–19.4) | −25.3 (−128.4 to 77.9) |
| Georgia | 262 (201–322) | 4.5 (3.5–5.6) | 324 (258–390) | 5.6 (4.5–6.8) | 16.4 (−47 to 79.8) |
| Kazakhstan | 237 (197–278) | 1.8 (1.5–2.2) | 6780 (5487–8073) | 39.3 (32.2–46.4) | 3421.6 (2347.7–4495.5) |
| Kyrgyzstan | 137 (115–160) | 4.3 (3.6–5) | 516 (425–607) | 9.3 (7.7–10.9) | 412.5 (241.9–583.2) |
| Mongolia | 141 (83–199) | 13.2 (7.7–18.7) | 367 (259–474) | 15 (10.9–19) | 21 (−79.6 to 121.5) |
| Tajikistan | 49 (35–64) | 1.6 (1.1–2.1) | 80 (53–106) | 1.2 (0.8–1.6) | −66.7 (−128.1 to −5.2) |
| Turkmenistan | 392 (347–436) | 18.7 (16.5–21) | 647 (498–796) | 14.7 (11.4–18.1) | −59.3 (−99.3 to −19.2) |
| Uzbekistan | 118 (95–141) | 0.9 (0.7–1.1) | 574 (471–678) | 2.1 (1.7–2.5) | 116.6 (35.5–197.7) |
| Central Europe | 22386 (20932–23840) | 17.6 (16.4–18.8) | 27189 (24843–29535) | 12.1 (11–13.2) | −42.3 (−59.5 to −25) |
| Albania | 205 (168–243) | 12.6 (10.4–14.9) | 333 (228–438) | 8.3 (5.7–11) | −55.2 (−115.8 to 5.4) |
| Bosnia and Herzegovina | 476 (307–644) | 14.9 (9.6–20.3) | 961 (632–1290) | 15.6 (10.3–20.9) | −4.2 (−80.1 to 71.7) |
| Bulgaria | 202 (181–223) | 2.5 (2.3–2.7) | 583 (481–684) | 4.3 (3.6–5.1) | 119.1 (61.6–176.6) |
| Croatia | 350 (317–382) | 7.2 (6.5–7.9) | 647 (558–736) | 7 (6–8) | 30.7 (−8.9 to 70.2) |
| Czechia | 415 (378–452) | 3.2 (2.9–3.5) | 624 (526–723) | 3 (2.5–3.4) | 128.7 (40.1–217.3) |
| Hungary | 2735 (2489–2981) | 21.4 (19.5–23.3) | 2072 (1787–2357) | 11.3 (9.6–12.9) | −86.9 (−108.6 to −65.2) |
| Montenegro | 137 (96–179) | 24 (16.9–31.1) | 230 (161–300) | 27.7 (19.2–36.3) | 12.3 (−66.1 to 90.7) |
| North Macedonia | 335 (251–418) | 21.3 (15.9–26.6) | 581 (396–765) | 22.8 (15.2–30.4) | −13.1 (−83.1 to 56.8) |
| Poland | 6719 (6426–7013) | 16.6 (15.8–17.4) | 9000 (8096–9903) | 12.1 (10.9–13.3) | −26.2 (−47.1 to −5.3) |
| Romania | 6814 (5840–7788) | 32.9 (28.2–37.6) | 7939 (6912–8965) | 20.8 (18.1–23.6) | −54.4 (−87.3 to −21.5) |
| Serbia | 2092 (1613–2571) | 26.6 (20.5–32.8) | 2771 (1853–3688) | 16.4 (11–21.8) | −57.5 (−107.4 to −7.6) |
| Slovakia | 234 (171–297) | 4.2 (3.1–5.3) | 445 (325–566) | 4.9 (3.6–6.3) | 69.4 (−37.1 to 175.8) |
| Slovenia | 1313 (1192–1435) | 55.6 (50.2–61) | 607 (498–716) | 11.3 (9.2–13.3) | −142.9 (−159.6 to −126.2) |
| Central Latin America | 2175 (2073–2277) | 2.4 (2.2–2.5) | 4263 (3766–4760) | 1.8 (1.5–2) | −43.9 (−59.8 to −28) |
| Colombia | 605 (573–637) | 3.2 (3–3.4) | 1060 (878–1243) | 1.9 (1.6–2.3) | −6 (−38.7 to 26.8) |
| Costa Rica | 90 (83–96) | 5 (4.6–5.4) | 174 (156–193) | 3.2 (2.8–3.5) | −46.8 (−68.5 to −25.1) |
| El Salvador | 76 (60–92) | 2.1 (1.6–2.5) | 95 (73–117) | 1.5 (1.1–1.8) | −55.4 (−109.3 to −1.5) |
| Guatemala | 113 (92–133) | 2.8 (2.4–3.2) | 180 (152–207) | 1.6 (1.4–1.8) | −85.6 (−106.8 to −64.5) |
| Honduras | 80 (67–93) | 3.3 (2.7–4) | 202 (143–261) | 3.3 (2.3–4.4) | −14.2 (−72.4 to 44) |
| Mexico | 461 (440–482) | 0.9 (0.8–0.9) | 1443 (1287–1598) | 1.2 (1.1–1.3) | 78.4 (47.5–109.2) |
| Nicaragua | 51 (41–61) | 2.3 (1.9–2.7) | 83 (66–101) | 1.7 (1.4–2.1) | −48.7 (−85.3 to −12.1) |
| Panama | 69 (64–75) | 4.4 (4.1–4.8) | 180 (144–217) | 4 (3.2–4.8) | −29.9 (−61.6 to 1.7) |
| Venezuela (Bolivarian Republic of) | 631 (582–681) | 6.3 (5.7–6.9) | 845 (653–1036) | 3 (2.3–3.7) | −114 (−131.2 to −96.8) |
| Central Sub-Saharan Africa | 3627 (2129–5125) | 15.7 (8–23.4) | 7529 (3662–11397) | 14.2 (6.3–22.1) | −34.5 (−80.3 to 11.4) |
| Angola | 664 (392–936) | 15.9 (8.4–23.4) | 1660 (964–2356) | 14.1 (7.8–20.3) | −47 (−101.2 to 7.3) |
| Central African Republic | 204 (111–297) | 17.7 (8.5–26.9) | 353 (157–549) | 16.1 (6.7–25.4) | −32 (−82.8 to 18.7) |
| Congo | 170 (111–230) | 16.4 (10.3–22.6) | 328 (193–463) | 13 (8–18) | −54.4 (−102.9 to −6) |
| Democratic Republic of the Congo | 2470 (1334–3606) | 15.4 (6.7–24) | 5021 (1992–8050) | 14.4 (5.1–23.7) | −26.4 (−83.7 to 30.9) |
| Equatorial Guinea | 32 (18–46) | 16.5 (8.3–24.6) | 58 (24–92) | 10.4 (4.5–16.4) | −94.2 (−153.6 to −34.9) |
| Gabon | 86 (52–121) | 16.2 (9.4–23) | 109 (60–158) | 11.4 (6.4–16.5) | −72 (−113.8 to −30.2) |
| East Asia | 7077 (3668–10487) | 0.9 (0.4–1.3) | 17440 (12991–21889) | 1 (0.7–1.2) | 63.7 (−68.1 to 195.5) |
| China | 6456 (3144–9767) | 0.8 (0.3–1.3) | 16573 (12148–20998) | 1 (0.7–1.2) | 95.6 (−76.9 to 268.1) |
| Democratic People's Republic of Korea | 236 (132–340) | 1.7 (0.8–2.6) | 439 (266–613) | 1.6 (0.9–2.3) | 9 (−69.9 to 87.9) |
| Taiwan (Province of China) | 386 (368–403) | 2.9 (2.7–3) | 428 (388–468) | 1.1 (1–1.2) | −112.1 (−120.1 to −104.1) |
| Eastern Europe | 30456 (28639–32272) | 12.2 (11.5–12.9) | 69698 (64590–74805) | 23.3 (21.6–25) | 290 (247.6–332.3) |
| Belarus | 1398 (1139–1656) | 11.6 (9.5–13.7) | 1261 (1051–1472) | 9.7 (8.1–11.4) | −28.5 (−77.2 to 20.2) |
| Estonia | 221 (187–254) | 11.7 (9.9–13.5) | 212 (188–236) | 8.8 (7.8–9.7) | −47.9 (−71.6 to −24.2) |
| Latvia | 497 (440–554) | 15.5 (13.7–17.2) | 871 (761–981) | 27.9 (24.3–31.4) | 160.4 (113–207.8) |
| Lithuania | 284 (244–325) | 6.7 (5.7–7.6) | 495 (434–556) | 10.6 (9.4–11.9) | 119.7 (70.2–169.1) |
| Republic of Moldova | 138 (124–152) | 3.2 (2.8–3.5) | 359 (311–406) | 6.5 (5.7–7.4) | 220.6 (165.7–275.5) |
| Russian Federation | 17086 (16726–17445) | 10.6 (10.3–10.8) | 52568 (48841–56295) | 25.6 (23.8–27.3) | 467.4 (396.3–538.5) |
| Ukraine | 10832 (9336–12328) | 17.6 (15.3–20) | 13931 (10969–16893) | 21.4 (16.8–26) | 93.6 (28.1–159.1) |
| Eastern Sub-Saharan Africa | 5024 (3628–6420) | 4.7 (3.5–5.9) | 8819 (6108–11530) | 4 (2.9–5.1) | −44 (−84.3 to −3.7) |
| Burundi | 212 (135–289) | 6.2 (4–8.4) | 283 (173–393) | 4.4 (2.8–6) | −63 (−121 to −5) |
| Comoros | 16 (9–22) | 5.4 (3.2–7.6) | 20 (12–29) | 3.8 (2.2–5.3) | −76.6 (−121.4 to −31.7) |
| Djibouti | 10 (6–15) | 5 (3–7.1) | 31 (16–47) | 4 (2.2–5.8) | −49.7 (−108.8 to 9.5) |
| Eritrea | 99 (62–137) | 5.4 (3.1–7.7) | 170 (101–239) | 4.8 (3–6.6) | −52.5 (−106 to 1) |
| Ethiopia | 1117 (710–1524) | 4 (2.6–5.5) | 1674 (1092–2256) | 3 (2–4) | −69.3 (−124.8 to −13.9) |
| Kenya | 334 (241–426) | 3 (2.3–3.8) | 785 (510–1059) | 3.1 (2.1–4.1) | −15.1 (−56.5 to 26.4) |
| Madagascar | 586 (394–778) | 8.1 (5.8–10.4) | 1078 (660–1496) | 6.8 (4.4–9.1) | −41.3 (−96.2 to 13.7) |
| Malawi | 239 (155–323) | 4 (2.9–5) | 384 (238–529) | 4 (2.6–5.4) | −4.9 (−69.4 to 59.6) |
| Mozambique | 270 (205–335) | 3.5 (2.7–4.2) | 552 (358–746) | 3.8 (2.6–5) | 6 (−77.2 to 89.1) |
| Rwanda | 268 (167–368) | 6.5 (4.2–8.8) | 304 (170–438) | 4.1 (2.4–5.7) | −88.3 (−135.8 to −40.7) |
| Somalia | 218 (128–308) | 5.2 (3.1–7.4) | 443 (225–661) | 4.6 (2.4–6.8) | −38.3 (−104.2 to 27.5) |
| South Sudan | 202 (130–275) | 5.5 (3.4–7.6) | 263 (140–386) | 4.3 (2.6–6) | −54.4 (−109.6 to 0.8) |
| Uganda | 499 (305–693) | 4.8 (3.2–6.3) | 767 (439–1095) | 3.5 (2.1–4.9) | −76.3 (−121.5 to −31.1) |
| United Republic of Tanzania | 765 (523–1007) | 5.3 (3.7–6.8) | 1347 (827–1867) | 4.1 (2.6–5.6) | −50.8 (−106.4 to 4.7) |
| Zambia | 184 (119–249) | 4.5 (3–6) | 710 (288–1132) | 7.7 (3.5–11.9) | 162.4 (−36 to 360.9) |
| High-income Asia Pacific | 10532 (9762–11303) | 6.1 (5.6–6.6) | 9990 (8541–11440) | 1.8 (1.6–2) | −130.8 (−135.3 to −126.3) |
| Brunei Darussalam | 12 (10–14) | 10.4 (8.1–12.6) | 20 (16–24) | 6.6 (5.3–7.9) | −81.4 (−109.1 to −53.7) |
| Japan | 9822 (9128–10516) | 6.6 (6.1–7.1) | 8660 (7301–10019) | 2 (1.8–2.2) | −125.5 (−130.4 to −120.7) |
| Republic of Korea | 572 (403–741) | 2.4 (1.6–3.1) | 1218 (971–1466) | 1.4 (1.1–1.7) | −95.3 (−133.8 to −56.8) |
| Singapore | 126 (119–133) | 5.9 (5.5–6.3) | 92 (82–102) | 1.1 (1–1.2) | −167 (−170.1 to −163.9) |
| High-income North America | 30154 (28619–31689) | 8.7 (8.3–9.1) | 30262 (27889–32635) | 4.7 (4.4–5.1) | −77.2 (−85.2 to −69.2) |
| Canada | 1103 (1027–1179) | 3.5 (3.3–3.7) | 1585 (1438–1733) | 2.2 (2–2.4) | −66.3 (−82.3 to −50.3) |
| Greenland | 5 (4–6) | 13.9 (11.5–16.4) | 6 (4–7) | 8.8 (6.9–10.8) | −41.5 (−99.2 to 16.3) |
| United States of America | 29045 (27555–30535) | 9.2 (8.8–9.7) | 28670 (26375–30966) | 5 (4.7–5.4) | −76.7 (−84.9 to −68.5) |
| North Africa and Middle East | 6745 (4646–8845) | 3 (1.7–4.3) | 8789 (6140–11437) | 2 (1.3–2.7) | −68.3 (−99 to −37.6) |
| Afghanistan | 191 (60–321) | 2.4 (0.6–4.3) | 338 (161–515) | 2.5 (0.7–4.2) | −23.1 (−92.8 to 46.6) |
| Algeria | 364 (222–506) | 2.7 (1.2–4.1) | 588 (309–868) | 2 (1–2.9) | −56.3 (−111.2 to −1.5) |
| Bahrain | 14 (12–16) | 8.9 (7.3–10.4) | 32 (24–40) | 5 (4–6) | −89 (−125 to −52.9) |
| Egypt | 1752 (736–2768) | 4.2 (1.3–7) | 1484 (746–2222) | 2.5 (1.1–3.8) | −94.4 (−134.1 to −54.7) |
| Iran (Islamic Republic of) | 620 (444–797) | 1.8 (1.2–2.4) | 911 (643–1179) | 1.3 (0.8–1.7) | −72.9 (−104.2 to −41.5) |
| Iraq | 578 (386–769) | 5.2 (3.2–7.2) | 943 (654–1232) | 4.1 (2.5–5.6) | −44.7 (−124.8 to 35.4) |
| Jordan | 18 (13–22) | 1 (0.7–1.2) | 29 (22–35) | 0.4 (0.3–0.5) | −112.3 (−148.5 to −76) |
| Kuwait | 19 (17–21) | 2.4 (2.1–2.6) | 17 (14–21) | 0.7 (0.6–0.8) | −127.3 (−143.9 to −110.7) |
| Lebanon | 34 (15–54) | 1.7 (0.7–2.6) | 65 (44–86) | 1 (0.7–1.3) | −92.6 (−160.6 to −24.7) |
| Libya | 90 (57–122) | 2.3 (1.4–3.3) | 83 (51–116) | 1.8 (1.1–2.5) | −46.5 (−122.4 to 29.4) |
| Morocco | 468 (271–664) | 2.6 (1–4.2) | 660 (289–1031) | 2.2 (0.9–3.4) | −33.9 (−94 to 26.2) |
| Oman | 69 (43–94) | 8.5 (5.1–11.9) | 100 (65–134) | 5 (3.6–6.5) | −95.2 (−154.6 to −35.9) |
| Palestine | 56 (41–72) | 5.5 (3.9–7.1) | 80 (57–104) | 3.6 (2.7–4.5) | −68.4 (−117.7 to −19.2) |
| Qatar | 8 (6–10) | 8.3 (5.6–11) | 23 (13–34) | 3 (1.9–4.1) | −109 (−159.1 to −58.9) |
| Saudi Arabia | 513 (355–671) | 7.4 (4.8–10.1) | 888 (633–1143) | 4.9 (3.5–6.2) | −79.7 (−135.5 to −24) |
| Sudan | 380 (152–608) | 2.6 (1–4.2) | 489 (272–706) | 2 (1–2.9) | −63.8 (−121.3 to −6.3) |
| Syrian Arab Republic | 238 (157–319) | 2.7 (1.4–4) | 224 (108–341) | 2 (0.9–3) | −58.9 (−120.7 to 2.8) |
| Tunisia | 120 (68–172) | 2.2 (1–3.4) | 218 (84–352) | 1.9 (0.7–3.1) | −31.6 (−91.8 to 28.6) |
| Turkey | 957 (620–1294) | 2.4 (1.5–3.3) | 1062 (702–1423) | 1.3 (0.8–1.7) | −86.2 (−138.4 to −34) |
| United Arab Emirates | 52 (34–71) | 9.4 (6.3–12.4) | 163 (119–207) | 6.2 (4.6–7.8) | −84.4 (−125.2 to −43.6) |
| Yemen | 202 (93–312) | 2.6 (0.8–4.3) | 381 (193–569) | 2.3 (0.9–3.6) | −45.1 (−106.1 to 16) |
| Oceania | 151 (92–210) | 4.2 (2.5–5.9) | 380 (231–528) | 4.2 (2.5–5.9) | −28 (−71 to 15) |
| American Samoa | 2 (2–2) | 8.1 (6.4–9.9) | 3 (3–4) | 7.4 (5.9–8.9) | −62.9 (−104.7 to −21.1) |
| Cook Islands | 0 (0–0) | 0.5 (0.4–0.7) | 0 (0–0) | 0.4 (0.3–0.5) | −100.1 (−137.8 to −62.4) |
| Fiji | 13 (10–16) | 3.2 (2.5–4) | 24 (17–30) | 3.2 (2.5–4) | −20.1 (−69.6 to 29.4) |
| Guam | 3 (3–4) | 4.4 (3.7–5.2) | 4 (4–5) | 2.2 (1.9–2.6) | −93.9 (−118 to −69.8) |
| Kiribati | 2 (1–3) | 4.8 (2.2–7.3) | 3 (2–5) | 4.6 (2.6–6.7) | −10.5 (−72.4 to 51.3) |
| Marshall Islands | 1 (1–1) | 5.2 (3–7.3) | 2 (1–2) | 4.5 (2.7–6.4) | −43.3 (−93.3 to 6.8) |
| Micronesia (Federated States of) | 3 (2–4) | 6.3 (3.4–9.2) | 3 (2–5) | 4.9 (3.2–6.7) | −57.8 (−107.1 to −8.5) |
| Nauru | 0 (0–0) | 6.5 (4.5–8.5) | 0 (0–1) | 7.4 (4.9–10) | −1.8 (−72.5 to 69) |
| Niue | 0 (0–0) | 4.7 (3.3–6.1) | 0 (0–0) | 4.6 (3.7–5.5) | −34.3 (−84.8 to 16.2) |
| Northern Mariana Islands | 1 (1–1) | 4.3 (3.2–5.5) | 2 (2–3) | 4.4 (3.5–5.4) | −37.4 (−98.4 to 23.5) |
| Palau | 0 (0–0) | 3.5 (2.5–4.5) | 1 (0–1) | 2.9 (2.2–3.6) | −48.1 (−96.1 to −0.1) |
| Papua New Guinea | 99 (53–145) | 4.2 (2.1–6.2) | 290 (157–422) | 4.5 (2.3–6.7) | −19.8 (−80.8 to 41.2) |
| Samoa | 4 (3–6) | 4.9 (3.2–6.6) | 6 (4–8) | 4.1 (3–5.2) | −50.5 (−92.8 to −8.1) |
| Solomon Islands | 5 (3–8) | 4 (2–6.1) | 14 (8–20) | 4.1 (2.2–6) | −18.6 (−76.8 to 39.7) |
| Tokelau | 0 (0–0) | 5.4 (3.3–7.5) | 0 (0–0) | 5.6 (3.8–7.4) | −27 (−87.4 to 33.5) |
| Tonga | 2 (1–2) | 3.3 (2.3–4.4) | 2 (2–3) | 2.7 (2–3.5) | −49.5 (−99.4 to 0.5) |
| Tuvalu | 0 (0–1) | 5.7 (3.3–8) | 0 (0–1) | 4.5 (3.1–5.8) | −58.9 (−104.6 to −13.2) |
| Vanuatu | 3 (2–5) | 4.4 (2.4–6.5) | 8 (5–11) | 4.1 (2.4–5.8) | −36.4 (−80.8 to 8) |
| South Asia | 27341 (15413–39269) | 4.8 (2.6–7) | 67758 (51893–83623) | 5 (3.9–6.2) | −10.2 (−65.4 to 45) |
| Bangladesh | 3219 (1413–5026) | 6.9 (2.8–11) | 7752 (4691–10813) | 6.4 (3.9–8.9) | −37.1 (−109.1 to 34.8) |
| Bhutan | 13 (4–21) | 5.6 (1.3–9.8) | 32 (19–45) | 5.6 (3.4–7.9) | −16.4 (−108.8 to 76) |
| India | 20463 (11933–28994) | 4.4 (2.5–6.2) | 51777 (39784–63769) | 4.7 (3.6–5.8) | −5.5 (−62 to 51) |
| Nepal | 516 (223–808) | 5.8 (2.1–9.4) | 1115 (650–1580) | 5.5 (3.1–7.8) | −27.2 (−108.4 to 53.9) |
| Pakistan | 3130 (1608–4652) | 5.2 (2.4–8) | 7083 (4698–9467) | 6.1 (3.9–8.2) | 11.8 (−60.3 to 83.8) |
| Southeast Asia | 8250 (6499–10001) | 3.9 (3.1–4.8) | 17143 (14539–19748) | 3.2 (2.7–3.6) | −30 (−73 to 13.1) |
| Cambodia | 121 (67–175) | 3.3 (1.8–4.8) | 294 (197–391) | 3.1 (2.2–4.1) | −8.8 (−82.6 to 65) |
| Indonesia | 2712 (1732–3691) | 3.4 (2.2–4.6) | 6849 (4995–8702) | 4.2 (3.1–5.2) | 38 (−31.7 to 107.8) |
| Lao People's Democratic Republic | 75 (38–111) | 4.7 (2.4–7) | 143 (95–192) | 3.9 (2.5–5.2) | −38.7 (−102.9 to 25.5) |
| Malaysia | 477 (367–588) | 5.2 (3.9–6.5) | 791 (664–918) | 3.2 (2.6–3.7) | −92.4 (−154.1 to −30.7) |
| Maldives | 3 (1–4) | 3.9 (2.3–5.5) | 8 (6–10) | 2.7 (2–3.4) | −80.2 (−140.5 to −20) |
| Mauritius | 11 (11–12) | 1.7 (1.6–1.8) | 75 (68–81) | 4.6 (4.2–4.9) | 176.5 (143.2–209.8) |
| Myanmar | 879 (521–1238) | 4.9 (3–6.8) | 1585 (1147–2024) | 4 (2.9–5.1) | −35.3 (−99.8 to 29.2) |
| Philippines | 1457 (1087–1826) | 6.9 (4.6–9.2) | 2950 (2260–3639) | 4.4 (3.2–5.5) | −54.2 (−85.1 to −23.3) |
| Seychelles | 6 (5–7) | 11.2 (9.3–13.1) | 6 (5–8) | 6.2 (5–7.4) | −63.5 (−105.9 to −21.1) |
| Sri Lanka | 928 (688–1167) | 9.4 (7.1–11.7) | 586 (238–933) | 2.4 (1–3.9) | −139.3 (−177 to −101.6) |
| Thailand | 154 (75–233) | 0.5 (0.2–0.7) | 704 (509–900) | 0.7 (0.5–0.9) | 126.2 (−53 to 305.4) |
| Timor-Leste | 10 (5–14) | 4.1 (2.1–6.1) | 29 (18–39) | 4.1 (2.7–5.6) | 4.7 (−72.7 to 82.1) |
| Viet Nam | 1406 (908–1904) | 4.1 (2.6–5.6) | 3100 (2266–3934) | 3.8 (2.8–4.9) | 7.5 (−89.5 to 104.5) |
| Southern Latin America | 6680 (6096–7265) | 15.5 (14.1–16.9) | 7430 (6755–8105) | 8.3 (7.6–9.1) | −126.5 (−133.2 to −119.7) |
| Uruguay | 460 (422–499) | 12 (11.1–13) | 325 (297–353) | 5.4 (5–5.9) | −117.4 (−126.5 to −108.3) |
| Argentina | 5652 (5098–6205) | 18.9 (17–20.8) | 6460 (5837–7083) | 11.3 (10.2–12.4) | −124.9 (−132.9 to −117) |
| Chile | 568 (538–598) | 5.8 (5.5–6.2) | 645 (592–697) | 2.6 (2.4–2.8) | −120.4 (−128.5 to −112.3) |
| Southern Sub-Saharan Africa | 3754 (2987–4522) | 15.1 (11.6–18.6) | 6525 (5783–7266) | 12.8 (11.3–14.2) | −42.8 (−84.6 to −1.1) |
| Botswana | 77 (55–99) | 16.5 (11.8–21.1) | 139 (98–180) | 11.6 (8.3–14.9) | −87 (−131.4 to −42.5) |
| Eswatini | 40 (28–51) | 15.6 (11.1–20.1) | 55 (35–75) | 10.9 (7.1–14.7) | −94.3 (−130.1 to −58.6) |
| Lesotho | 100 (61–140) | 13.9 (8.4–19.3) | 115 (72–158) | 12.6 (8.1–17.1) | −64.8 (−123 to −6.7) |
| Namibia | 87 (67–107) | 16.4 (12.5–20.4) | 155 (114–196) | 13.5 (10–16.9) | −64.6 (−112.1 to −17.2) |
| South Africa | 2941 (2289–3594) | 15.1 (11.3–18.9) | 5128 (4477–5779) | 12.4 (10.9–14) | −50.2 (−95.6 to −4.9) |
| Zimbabwe | 509 (398–620) | 15.3 (11.8–18.7) | 933 (685–1181) | 15.4 (11.4–19.3) | −14.1 (−86.9 to 58.6) |
| Tropical Latin America | 13390 (12827–13953) | 15.6 (14.8–16.5) | 17353 (16070–18636) | 6.9 (6.4–7.5) | −125.9 (−129.9 to −121.9) |
| Brazil | 13252 (12698–13807) | 15.9 (15.1–16.8) | 17132 (15861–18403) | 7 (6.5–7.6) | −126.3 (−130.3 to −122.3) |
| Paraguay | 137 (111–164) | 6.1 (4.8–7.3) | 221 (167–275) | 3.9 (3–4.9) | −76.7 (−125.9 to −27.6) |
| Western Europe | 58911 (54467–63355) | 10.4 (9.6–11.1) | 35955 (32312–39598) | 3.4 (3.1–3.7) | −130 (−135.7 to −124.3) |
| Andorra | 4 (3–6) | 10 (6.5–13.5) | 9 (6–12) | 5.4 (3.7–7) | −96 (−135.3 to −56.7) |
| Austria | 4690 (4320–5059) | 40 (36.8–43.1) | 1574 (1376–1771) | 7.1 (6.3–7.9) | −166.9 (−169.4 to −164.5) |
| Belgium | 1358 (1232–1485) | 8.8 (8–9.7) | 938 (812–1064) | 3.2 (2.8–3.6) | −86.2 (−97.4 to −75) |
| Cyprus | 43 (30–56) | 8.5 (5.6–11.3) | 63 (48–79) | 3.7 (2.8–4.7) | −114.2 (−145.3 to −83.2) |
| Denmark | 217 (199–236) | 2.7 (2.5–3) | 170 (152–189) | 1.4 (1.2–1.5) | −97.2 (−110.4 to −83.9) |
| Finland | 444 (398–491) | 6.7 (6–7.5) | 516 (471–562) | 4.3 (4–4.6) | −72.2 (−90.4 to −54) |
| France | 6608 (6114–7102) | 7.6 (7–8.1) | 5615 (4911–6318) | 3.1 (2.7–3.4) | −85.2 (−97.8 to −72.7) |
| Germany | 12314 (10986–13641) | 9.9 (8.9–11) | 9627 (8541–10713) | 4.5 (4.1–5) | −111.3 (−122.7 to −99.8) |
| Greece | 784 (722–846) | 5.8 (5.3–6.3) | 765 (688–842) | 3.1 (2.8–3.3) | −69.3 (−91.6 to −47) |
| Iceland | 9 (8–10) | 3 (2.8–3.3) | 8 (7–9) | 1.3 (1.1–1.4) | −111.8 (−121.8 to −101.8) |
| Ireland | 499 (464–534) | 13.6 (12.6–14.7) | 313 (271–355) | 3.8 (3.3–4.3) | −137.3 (−144.9 to −129.7) |
| Israel | 148 (135–161) | 3.4 (3.1–3.7) | 140 (122–157) | 1 (0.9–1.2) | −115.6 (−127.3 to −103.9) |
| Italy | 20621 (18561–22680) | 25.4 (22.7–28.2) | 4803 (4075–5531) | 2.7 (2.3–3.1) | −131.6 (−142.5 to −120.7) |
| Luxembourg | 47 (44–50) | 9.4 (8.8–10) | 33 (29–36) | 2.7 (2.4–3) | −132.1 (−139.8 to −124.4) |
| Malta | 50 (46–54) | 13.3 (12.1–14.5) | 23 (20–26) | 2.2 (1.9–2.4) | −149.2 (−156.5 to −142) |
| Monaco | 11 (8–14) | 14.4 (10.4–18.4) | 8 (6–11) | 7.4 (5.3–9.4) | −98.1 (−130.3 to −65.9) |
| Netherlands | 1937 (1778–2096) | 9.9 (9–10.7) | 1067 (940–1195) | 2.8 (2.5–3.1) | −143.7 (−148.5 to −139) |
| Norway | 108 (102–114) | 1.8 (1.7–1.9) | 179 (162–197) | 1.7 (1.6–1.9) | −45.3 (−54.7 to −36) |
| Portugal | 531 (496–566) | 4.5 (4.2–4.9) | 646 (572–719) | 2.4 (2.2–2.7) | −74.3 (−88.9 to −59.7) |
| San Marino | 3 (3–4) | 9.4 (7–11.8) | 3 (2–4) | 3.2 (2–4.3) | −116.4 (−150.1 to −82.7) |
| Spain | 5441 (4990–5892) | 10.7 (9.8–11.7) | 5085 (4427–5743) | 4.5 (4–5) | −113.4 (−122.6 to −104.2) |
| Sweden | 460 (435–486) | 3.4 (3.2–3.6) | 465 (409–521) | 2.1 (1.9–2.4) | 29.5 (−8.4 to 67.5) |
| Switzerland | 494 (439–549) | 4.7 (4.2–5.2) | 399 (346–452) | 1.8 (1.6–2.1) | −122.1 (−135.1 to −109) |
| United Kingdom | 2040 (1958–2122) | 2.5 (2.4–2.6) | 3472 (3170–3774) | 2.6 (2.4–2.8) | −0.5 (−9.2 to 8.3) |
| Western Sub-Saharan Africa | 12038 (8505–15570) | 13.9 (9.5–18.3) | 17612 (13805–21418) | 8.7 (7–10.3) | −98.1 (−121.8 to −74.4) |
| Benin | 231 (146–315) | 10.9 (6.6–15.2) | 395 (283–508) | 7.3 (5.2–9.4) | −84.5 (−133.3 to −35.8) |
| Burkina Faso | 635 (333–936) | 15.6 (7.6–23.7) | 1054 (671–1436) | 11.9 (7.4–16.4) | −77.3 (−128.7 to −25.9) |
| Cabo Verde | 11 (7–14) | 4.2 (2.6–5.9) | 15 (11–19) | 3.4 (2.4–4.3) | −46.6 (−152.1 to 58.8) |
| Cameroon | 587 (402–773) | 13.5 (9.1–17.9) | 1148 (797–1500) | 8.7 (6.1–11.3) | −87.1 (−137.7 to −36.5) |
| Chad | 362 (174–550) | 12.7 (5.7–19.6) | 616 (364–868) | 9.9 (5.6–14.2) | −68.4 (−119.9 to −17) |
| Côte d'Ivoire | 568 (378–757) | 13.7 (9.1–18.3) | 1045 (714–1376) | 9 (6.5–11.5) | −91.7 (−132.3 to −51) |
| Gambia | 47 (28–67) | 13.2 (7.8–18.6) | 96 (70–122) | 9.4 (6.9–12) | −83.3 (−136 to −30.6) |
| Ghana | 1044 (779–1309) | 16.7 (12.1–21.2) | 2490 (1719–3261) | 15.3 (10.7–19.8) | −53.4 (−110.6 to 3.9) |
| Guinea | 435 (259–610) | 12.9 (7.4–18.5) | 574 (383–765) | 9.7 (6.4–13) | −68.9 (−122.8 to −15) |
| Guinea-Bissau | 64 (38–89) | 15.4 (9.3–21.5) | 79 (56–103) | 10.2 (7.2–13.3) | −90.6 (−140.1 to −41.1) |
| Liberia | 166 (101–232) | 13.7 (7.9–19.5) | 188 (117–259) | 8.7 (5.4–12.1) | −89.3 (−141.5 to −37.1) |
| Mali | 415 (233–598) | 10.5 (5.4–15.6) | 682 (436–929) | 7.1 (4.5–9.6) | −91.4 (−133.3 to −49.4) |
| Mauritania | 129 (89–169) | 13.2 (8.9–17.6) | 174 (118–229) | 8.3 (5.8–10.9) | −97.4 (−142.7 to −52.1) |
| Niger | 360 (182–538) | 12.8 (5.4–20.3) | 731 (373–1088) | 9.1 (4.5–13.6) | −88.3 (−131.4 to −45.1) |
| Nigeria | 6037 (4017–8057) | 14.1 (9.3–19) | 6900 (4837–8963) | 7.1 (5.3–8.9) | −123 (−147.6 to −98.4) |
| Sao Tome and Principe | 6 (4–9) | 10.4 (6.3–14.5) | 8 (6–10) | 7.4 (5.5–9.4) | −83 (−139.3 to −26.6) |
| Senegal | 458 (277–640) | 13.6 (8.1–19.1) | 715 (494–936) | 9.3 (6.3–12.3) | −90 (−137.8 to −42.1) |
| Sierra Leone | 309 (189–429) | 14 (8–20) | 371 (241–501) | 9.1 (6.1–12.2) | −93.2 (−138.3 to −48.1) |
| Togo | 173 (111–234) | 13.2 (8.4–18.1) | 330 (214–446) | 9 (6.1–11.8) | −80.1 (−133 to −27.2) |
