# Supplementary material for: Global, regional, and national burden of cardiomyopathy (including alcoholic cardiomyopathy and others) from 1990 to 2021: An analysis of data from the global burden of disease study 2021 and forecast to 2040
Source: PLoS One. 2026 Jan 30;21(1):e0341687. doi: 10.1371/journal.pone.0341687 (PMC12858021; doi:10.1371/journal.pone.0341687)
Supplement: S9 Table — (DOCX) [file pone.0341687.s020.docx]

**S9 Table. 1990–2021 Global and national DALYs trends in total cardiomyopathy burden.**

| location_name | Number_1990 | ASR per 100,000_1990 | Number_2021 | ASR per 100,000_2021 | Percentage change in the ASRs per 100,000 |
| --- | --- | --- | --- | --- | --- |
| Global | 7541688 (6684606–8398771) | 175.1 (157.8–192.4) | 10690819 (9864323–11517314) | 129.8 (119.7–140) | −54.3 (−66.3 to −42.2) |
| Andean Latin America | 26400 (20934–31866) | 71.7 (60.2–83.2) | 23695 (19321–28070) | 38.1 (31–45.1) | −102.3 (−128.4 to −76.2) |
| Bolivia (Plurinational State of) | 7060 (4027–10092) | 103.6 (65.7–141.5) | 6868 (5051–8685) | 64.8 (47.7–81.9) | −70.6 (−140.8 to −0.4) |
| Ecuador | 5103 (4577–5629) | 59.3 (52.9–65.6) | 7297 (5565–9029) | 43.3 (33.3–53.4) | −103.3 (−127.5 to −79.1) |
| Peru | 14237 (11326–17149) | 67.7 (55.7–79.7) | 9531 (6910–12151) | 27.5 (20–35) | −109.7 (−151.7 to −67.8) |
| Australasia | 41536 (39505–43567) | 190.8 (181.6–199.9) | 38792 (36038–41545) | 88.1 (81.7–94.6) | −82.1 (−91.8 to −72.3) |
| Australia | 33661 (32003–35319) | 185.7 (176.1–195.2) | 30745 (28358–33132) | 82.8 (76.3–89.2) | −78.5 (−88.7 to −68.3) |
| New Zealand | 7874 (7160–8589) | 217.2 (197.7–236.7) | 8047 (7509–8585) | 115.4 (107.8–122.9) | −88.3 (−103.6 to −72.9) |
| Caribbean | 47803 (35101–60506) | 151.6 (117.7–185.5) | 91042 (73173–108910) | 180.8 (141.8–219.8) | 193.1 (117.9–268.3) |
| Antigua and Barbuda | 60 (54–65) | 111.4 (100.8–121.9) | 165 (152–178) | 157.5 (146.2–168.7) | 563.7 (404.6–722.8) |
| Bahamas | 502 (451–553) | 249.8 (223.3–276.3) | 1299 (1081–1516) | 304.1 (254.1–354.1) | 156.3 (57.6–255) |
| Barbados | 514 (466–563) | 194.2 (175.5–213) | 809 (676–942) | 180.3 (151–209.6) | 11 (−35.8 to 57.9) |
| Belize | 212 (184–240) | 139.3 (117.6–161) | 652 (586–718) | 181.9 (163.9–199.9) | 500.3 (253.5–747.2) |
| Bermuda | 90 (75–105) | 144.2 (119.9–168.5) | 192 (166–219) | 166.1 (143.2–189.1) | 453.2 (192.6–713.9) |
| Cuba | 8131 (7337–8925) | 78.7 (71–86.4) | 28631 (25502–31761) | 168.7 (151–186.4) | 675.6 (517.8–833.3) |
| Dominica | 236 (187–285) | 389 (307.7–470.4) | 370 (291–449) | 470.5 (372.5–568.4) | 49.7 (−41.7 to 141.1) |
| Dominican Republic | 8563 (6886–10241) | 123.7 (100.4–147) | 12230 (9395–15065) | 114.5 (88–140.9) | 105.2 (−50.4 to 260.9) |
| Grenada | 184 (167–201) | 252.6 (228.7–276.4) | 384 (341–426) | 337.2 (301.6–372.9) | 107.7 (53.8–161.6) |
| Guyana | 1532 (1384–1679) | 274.4 (247.2–301.6) | 2118 (1701–2535) | 299.3 (240.8–357.7) | 130.2 (28.1–232.3) |
| Haiti | 15517 (4956–26079) | 296 (148.6–443.4) | 29343 (14515–44171) | 282.4 (148.1–416.6) | −2.9 (−73.4 to 67.7) |
| Jamaica | 2684 (2373–2994) | 135 (119.2–150.8) | 4319 (3461–5178) | 142.5 (113.9–171) | 72.5 (−8.2 to 153.1) |
| Puerto Rico | 5545 (5188–5902) | 155.1 (145.3–165) | 3241 (2818–3664) | 61.6 (53.6–69.5) | 57.1 (4.2–110) |
| Saint Kitts and Nevis | 122 (114–129) | 334.3 (312.6–356) | 215 (179–250) | 316.4 (268.4–364.5) | 324.8 (171.1–478.5) |
| Saint Lucia | 289 (262–316) | 303.4 (274.9–332) | 725 (625–825) | 316.9 (273.8–360) | 221.4 (115.8–327) |
| Saint Vincent and the Grenadines | 78 (72–85) | 91.4 (84.7–98.1) | 186 (166–207) | 139 (124–154) | 234.4 (165.6–303.2) |
| Suriname | 510 (432–588) | 157.5 (130.2–184.8) | 861 (654–1068) | 140.5 (108.1–172.9) | 66.9 (−58.7 to 192.6) |
| Trinidad and Tobago | 1156 (1059–1253) | 121.2 (111.1–131.3) | 2006 (1619–2393) | 114.9 (92.9–136.9) | 96.1 (−3.4 to 195.5) |
| United States Virgin Islands | 259 (192–325) | 269.5 (202.5–336.5) | 214 (157–271) | 181.8 (132.4–231.3) | −26.3 (−105.9 to 53.2) |
| Central Asia | 79367 (69767–88966) | 141.6 (123.9–159.3) | 359966 (301502–418430) | 386.1 (325.6–446.5) | 243.4 (157.3–329.5) |
| Armenia | 5881 (4794–6969) | 197.7 (161.2–234.1) | 4976 (3915–6037) | 128.3 (101.7–154.9) | −40.5 (−99.8 to 18.8) |
| Azerbaijan | 23780 (15976–31584) | 391.1 (257.8–524.4) | 47468 (24048–70888) | 408.3 (216.2–600.4) | −26.2 (−127.1 to 74.6) |
| Georgia | 7431 (5736–9126) | 125.9 (97.1–154.6) | 8184 (6390–9979) | 163.2 (128.5–197.8) | 25.6 (−40.5 to 91.8) |
| Kazakhstan | 9277 (7744–10810) | 62.8 (52.3–73.2) | 217646 (173412–261880) | 1123.6 (906.6–1340.6) | 2947.1 (1970.7–3923.6) |
| Kyrgyzstan | 5405 (4584–6227) | 160.7 (134.5–186.9) | 19737 (16392–23082) | 322.4 (267.3–377.5) | 310.5 (182.1–438.9) |
| Mongolia | 5013 (2910–7116) | 378.4 (223.1–533.7) | 12986 (9111–16860) | 437.3 (311.8–562.8) | 29.1 (−77.5 to 135.8) |
| Tajikistan | 1917 (1209–2625) | 49.5 (33.3–65.7) | 3401 (2279–4522) | 40 (26.8–53.1) | −56.4 (−123.4 to 10.5) |
| Turkmenistan | 15455 (13727–17183) | 588.6 (521.2–656) | 24678 (19197–30160) | 501.6 (389.8–613.4) | −51.5 (−93.8 to −9.2) |
| Uzbekistan | 5207 (4206–6208) | 31.2 (25.1–37.3) | 20890 (17137–24644) | 65.4 (53.7–77.1) | 83.2 (11.6–154.7) |
| Central Europe | 459793 (433966–485620) | 340.1 (321–359.1) | 536150 (485550–586750) | 268 (241.3–294.7) | −23.3 (−43.1 to −3.4) |
| Albania | 4486 (3648–5324) | 219.6 (179.4–259.8) | 6170 (4121–8219) | 160.9 (109.4–212.4) | −36.3 (−106.1 to 33.5) |
| Bosnia and Herzegovina | 11034 (7227–14842) | 289.4 (192.4–386.4) | 17682 (11335–24028) | 309.5 (197.2–421.8) | 4.3 (−72 to 80.7) |
| Bulgaria | 5025 (4513–5538) | 52.6 (48.2–57) | 11213 (8983–13444) | 92 (73.8–110.2) | 138.3 (68.7–208) |
| Croatia | 6389 (5874–6905) | 120.6 (110.8–130.5) | 11866 (9987–13746) | 143.1 (119.4–166.9) | 65.4 (16.2–114.7) |
| Czechia | 10298 (9455–11140) | 82 (75.5–88.6) | 14055 (11662–16448) | 76.7 (63.2–90.2) | 146.1 (55.8–236.4) |
| Hungary | 58929 (53362–64496) | 450.1 (408.5–491.7) | 50303 (42549–58058) | 311.4 (261.4–361.4) | −70 (−95.2 to −44.7) |
| Montenegro | 3049 (2146–3953) | 504.2 (357.9–650.5) | 4563 (3235–5891) | 530.6 (381.8–679.4) | 2.8 (−62.4–68) |
| North Macedonia | 7407 (5565–9248) | 424.6 (325.2–524.1) | 12194 (7781–16607) | 428.1 (292–564.2) | −14.7 (−81.5 to 52) |
| Poland | 142854 (137550–148158) | 346.2 (333.3–359.2) | 172520 (154491–190548) | 262.2 (233.8–290.6) | −0.2 (−26.7 to 26.3) |
| Romania | 134187 (117353–151020) | 568.9 (498.8–638.9) | 157711 (135465–179956) | 466.4 (396.3–536.6) | −36.8 (−70.4 to −3.2) |
| Serbia | 41300 (32313–50286) | 458.8 (360.5–557.1) | 50323 (36319–64328) | 327.8 (241.1–414.5) | −40.8 (−88.5 to 6.9) |
| Slovakia | 5960 (4448–7473) | 106.1 (79.6–132.6) | 10713 (7428–13997) | 128.3 (88.8–167.7) | 71.4 (−33.1 to 175.8) |
| Slovenia | 21521 (19545–23498) | 898.7 (814.2–983.3) | 9032 (7420–10644) | 203.7 (167.1–240.3) | −141.2 (−157.8 to −124.7) |
| Central Latin America | 92469 (87637–97301) | 70.9 (67.8–74.1) | 138930 (122285–155576) | 56.5 (49.4–63.6) | −29.6 (−46.7 to −12.5) |
| Colombia | 23713 (22321–25104) | 94.4 (89.7–99) | 29249 (24555–33943) | 56.8 (47.5–66) | −2.7 (−33.2 to 27.8) |
| Costa Rica | 2793 (2609–2978) | 129.4 (120.6–138.1) | 4607 (4162–5052) | 86.8 (78.4–95.3) | −40 (−62.5 to −17.5) |
| El Salvador | 3541 (2745–4337) | 72.5 (57.9–87.2) | 2952 (2309–3595) | 46.8 (36.7–57) | −60.2 (−109.7 to −10.7) |
| Guatemala | 6053 (4787–7318) | 83.8 (69.8–97.7) | 7729 (6523–8935) | 56.8 (48.1–65.6) | −72.3 (−96.6 to −48) |
| Honduras | 3581 (3023–4139) | 100.7 (84.1–117.3) | 6721 (4727–8714) | 89 (64–114.1) | −31.3 (−79.5 to 17) |
| Mexico | 24852 (23087–26617) | 31.9 (30.2–33.6) | 56107 (50315–61899) | 45.3 (40.6–49.9) | 97.2 (64.6–129.7) |
| Nicaragua | 2825 (2081–3568) | 80.1 (65.8–94.3) | 2896 (2362–3429) | 51.4 (42–60.7) | −61.4 (−96 to −26.7) |
| Panama | 2428 (2229–2628) | 128.4 (118.3–138.6) | 4965 (4036–5893) | 113.8 (92.6–134.9) | −28.6 (−59.8 to 2.7) |
| Venezuela (Bolivarian Republic of) | 22684 (21208–24159) | 166.8 (154.7–178.8) | 23705 (18607–28803) | 85.1 (66.9–103.3) | −106.2 (−124.6 to −87.7) |
| Central Sub-Saharan Africa | 157251 (102617–211885) | 417.3 (240.6–594.1) | 285029 (150953–419104) | 361.4 (177–545.8) | −31.1 (−63.3 to 1.1) |
| Angola | 29407 (16678–42137) | 431.3 (248.2–614.3) | 67601 (41023–94179) | 361.9 (210.6–513.1) | −43.7 (−87.7 to 0.2) |
| Central African Republic | 8793 (5061–12524) | 472.2 (250.3–694.1) | 14939 (7429–22450) | 431.2 (194.1–668.2) | −26.7 (−64.9 to 11.6) |
| Congo | 6644 (4311–8978) | 432.3 (285–579.6) | 11998 (7022–16974) | 335.2 (202.1–468.3) | −51.4 (−87.6 to −15.2) |
| Democratic Republic of the Congo | 108455 (68025–148886) | 408.2 (211.5–605) | 184477 (84363–284590) | 361.7 (149.2–574.2) | −23.9 (−62 to 14.1) |
| Equatorial Guinea | 1264 (752–1776) | 437.2 (242.6–631.8) | 2417 (933–3901) | 280.2 (121.6–438.7) | −89.3 (−141.9 to −36.7) |
| Gabon | 2687 (1688–3686) | 394.3 (240.9–547.7) | 3597 (1936–5259) | 286.1 (159.7–412.6) | −73.2 (−106.1 to −40.3) |
| East Asia | 343948 (200682–487215) | 32.5 (18.8–46.3) | 475010 (368813–581207) | 28.2 (22.3–34.1) | 52.3 (−76.1 to 180.8) |
| China | 321436 (179067–463805) | 31.2 (17.1–45.3) | 450153 (343896–556411) | 27.7 (21.7–33.7) | 72.8 (−90.2 to 235.7) |
| Democratic People's Republic of Korea | 9721 (6304–13137) | 51.2 (31.9–70.4) | 12928 (8326–17529) | 45.6 (29–62.3) | 6.6 (−74.8 to 87.9) |
| Taiwan (Province of China) | 12792 (12202–13382) | 75.3 (72–78.5) | 11929 (11032–12826) | 37.6 (34.9–40.4) | −85 (−95.8 to −74.3) |
| Eastern Europe | 1058290 (989084–1127495) | 416 (389.6–442.4) | 2377495 (2203109–2551880) | 864.2 (803–925.5) | 411.8 (359–464.6) |
| Belarus | 48373 (38620–58126) | 410.8 (329.4–492.2) | 49341 (40987–57696) | 410.4 (341.3–479.5) | 21 (−58.6 to 100.6) |
| Estonia | 8084 (6758–9410) | 440.8 (367.5–514.2) | 5286 (4685–5886) | 263.5 (233.6–293.5) | −77.8 (−97.9 to −57.7) |
| Latvia | 18765 (16565–20964) | 606.1 (536.4–675.7) | 25851 (22507–29196) | 981.6 (855.8–1107.3) | 124.3 (81.4–167.2) |
| Lithuania | 10392 (8813–11971) | 251.9 (213.5–290.3) | 14630 (12778–16483) | 366.4 (321.1–411.7) | 96.8 (48.4–145.1) |
| Republic of Moldova | 5542 (4969–6116) | 122.5 (110–135.1) | 11969 (10356–13582) | 232.7 (201.9–263.5) | 187.3 (134.9–239.7) |
| Russian Federation | 590892 (579660–602124) | 353.2 (346.3–360.1) | 1843566 (1708651–1978481) | 966.1 (898.3–1034) | 722.2 (619–825.4) |
| Ukraine | 376242 (319296–433188) | 612 (520.7–703.3) | 426851 (324274–529429) | 727.2 (553.5–901) | 96.2 (23.2–169.3) |
| Eastern Sub-Saharan Africa | 280297 (192018–368576) | 169.8 (126.3–213.4) | 443195 (302143–584248) | 142.7 (101.5–183.8) | −24.3 (−51.8 to 3.2) |
| Burundi | 11903 (7272–16534) | 228.5 (145.8–311.2) | 14439 (8782–20095) | 150 (95.5–204.5) | −40.1 (−75.1 to −5.2) |
| Comoros | 879 (477–1280) | 205.2 (117.5–292.9) | 891 (542–1241) | 138 (83.9–192.2) | −50.2 (−80.1 to −20.4) |
| Djibouti | 586 (346–827) | 181 (109.6–252.4) | 1448 (758–2139) | 141.4 (78.4–204.4) | −32.4 (−67.4 to 2.6) |
| Eritrea | 5598 (3663–7533) | 203.5 (128.1–278.9) | 8300 (4788–11813) | 166.6 (103.4–229.8) | −33.4 (−75.2 to 8.4) |
| Ethiopia | 63961 (42111–85812) | 149.1 (98.5–199.8) | 87762 (59424–116100) | 112.3 (77.3–147.3) | −27.5 (−65.9 to 10.9) |
| Kenya | 17610 (12691–22530) | 104.7 (78.7–130.7) | 34908 (23706–46110) | 101.5 (69.4–133.5) | −14.5 (−39 to 9.9) |
| Madagascar | 31506 (20231–42780) | 301.6 (206.5–396.7) | 55108 (33751–76465) | 250.2 (159.7–340.7) | −28.6 (−62.2 to 4.9) |
| Malawi | 14427 (8695–20160) | 149.5 (104.4–194.5) | 18848 (11621–26076) | 141.9 (90.5–193.3) | −10.2 (−44.7 to 24.3) |
| Mozambique | 13925 (9463–18386) | 121.6 (94.6–148.6) | 26305 (17101–35510) | 131.3 (87.5–175.1) | 1.6 (−40.3 to 43.5) |
| Rwanda | 15041 (9373–20708) | 239.1 (152.9–325.3) | 14043 (7895–20192) | 140.4 (82.1–198.7) | −55.9 (−91.6 to −20.2) |
| Somalia | 13019 (7707–18330) | 191.5 (114.9–268.1) | 24789 (12988–36590) | 163.6 (85.7–241.5) | −28.3 (−70.7 to 14.1) |
| South Sudan | 11231 (6537–15925) | 205.2 (130.8–279.5) | 15366 (7365–23368) | 171.4 (98.7–244.1) | −34.2 (−71.1 to 2.6) |
| Uganda | 29543 (16129–42957) | 175.2 (114.5–235.9) | 42253 (24403–60102) | 127 (75.7–178.3) | −43.2 (−76.4 to −10) |
| United Republic of Tanzania | 40591 (26464–54719) | 183 (130.1–236) | 66811 (40700–92921) | 145.9 (91.1–200.6) | −34.1 (−65 to −3.2) |
| Zambia | 10277 (6440–14114) | 154.4 (102.1–206.7) | 31537 (12850–50224) | 260.2 (111–409.3) | 86.4 (−37.4 to 210.1) |
| High-income Asia Pacific | 255971 (242395–269546) | 141.4 (133–149.8) | 177106 (159462–194751) | 49.3 (45.3–53.2) | −126.6 (−131.4 to −121.7) |
| Brunei Darussalam | 478 (385–572) | 269.4 (217.4–321.5) | 695 (568–822) | 173.8 (143.8–203.8) | −78.3 (−106.4 to −50.3) |
| Japan | 230739 (220648–240829) | 159.5 (152.2–166.8) | 146537 (131276–161797) | 54.3 (50.5–58.2) | −122.4 (−127.1 to −117.7) |
| Republic of Korea | 20597 (13543–27650) | 61.2 (42.2–80.2) | 27703 (22651–32754) | 38.1 (31–45.2) | −86.8 (−125.3 to −48.3) |
| Singapore | 4157 (3947–4367) | 161.9 (153.9–169.8) | 2172 (1970–2374) | 28.1 (25.4–30.8) | −168.3 (−171.2 to −165.4) |
| High-income North America | 799842 (770994–828689) | 249.4 (240.7–258) | 697089 (662671–731507) | 132.1 (125.7–138.5) | −76.4 (−83.5 to −69.2) |
| Canada | 31358 (29339–33378) | 103.9 (97.3–110.5) | 37079 (34403–39755) | 64.2 (59.5–69) | −65.1 (−80.2 to −50) |
| Greenland | 208 (168–248) | 444 (364.6–523.4) | 172 (134–210) | 254.8 (201.6–308.1) | −53.4 (−104.8 to −2) |
| United States of America | 768257 (740765–795749) | 264.8 (255.6–274) | 659827 (626402–693252) | 140 (133.3–146.8) | −76.3 (−83.7 to −68.9) |
| North Africa and Middle East | 390045 (227333–552756) | 112 (76.7–147.2) | 349329 (270035–428622) | 64.7 (48.5–80.8) | −78.8 (−109.3 to −48.3) |
| Afghanistan | 9238 (3673–14804) | 84.3 (29.6–138.9) | 18911 (10132–27690) | 77.4 (33–121.9) | −39.3 (−101.9 to 23.2) |
| Algeria | 20220 (12938–27502) | 85.1 (53.4–116.8) | 19823 (11938–27708) | 51.9 (30.7–73.1) | −74.8 (−121 to −28.6) |
| Bahrain | 630 (551–710) | 213.3 (184.5–242) | 1140 (846–1434) | 114.1 (91–137.2) | −95.4 (−129.7 to −61.1) |
| Egypt | 118802 (41450–196153) | 179.9 (76.7–283) | 71204 (40994–101414) | 77.8 (41.6–114) | −107.5 (−148.9 to −66.1) |
| Iran (Islamic Republic of) | 36865 (23645–50086) | 67.6 (48.6–86.6) | 28919 (23879–33959) | 36.9 (29.9–43.9) | −83.5 (−113.5 to −53.6) |
| Iraq | 29958 (20059–39856) | 176.3 (122.7–229.9) | 37417 (28630–46203) | 118.6 (87.2–150) | −66.6 (−129.1 to −4.1) |
| Jordan | 1026 (745–1307) | 31 (24–38) | 1264 (996–1533) | 13.2 (10.4–16.1) | −116.9 (−150.3 to −83.6) |
| Kuwait | 1065 (968–1161) | 82.9 (75.8–90) | 642 (524–759) | 19.9 (16.5–23.2) | −137.2 (−151.3 to −123.1) |
| Lebanon | 1163 (525–1801) | 45.7 (20.6–70.8) | 1486 (1163–1808) | 25.5 (20.1–30.9) | −104.3 (−158.9 to −49.8) |
| Libya | 6613 (4041–9184) | 124.8 (80.2–169.4) | 4267 (2709–5825) | 87.2 (52.1–122.3) | −54.8 (−117.1 to 7.5) |
| Morocco | 25266 (14158–36374) | 96.2 (56.3–136.1) | 20898 (10769–31026) | 61.6 (31.7–91.6) | −61.4 (−113.8 to −9) |
| Oman | 3158 (1994–4322) | 236.6 (149.3–323.9) | 3801 (2556–5047) | 136.1 (91.7–180.5) | −97.3 (−160.2 to −34.4) |
| Palestine | 2671 (1807–3535) | 143.4 (103.7–183) | 3177 (2188–4165) | 89 (64.7–113.2) | −75.3 (−117.6 to −33.1) |
| Qatar | 385 (299–471) | 184.9 (134.9–235) | 1033 (618–1449) | 71 (45.5–96.4) | −104.2 (−158.7 to −49.6) |
| Saudi Arabia | 24031 (16199–31863) | 211.7 (146.8–276.7) | 33725 (23860–43589) | 124.6 (91.5–157.8) | −83.3 (−135.9 to −30.7) |
| Sudan | 23739 (5089–42388) | 99.4 (42–156.7) | 26410 (16773–36047) | 68.4 (40.6–96.1) | −74.9 (−134.9 to −14.8) |
| Syrian Arab Republic | 15167 (10175–20159) | 104.8 (70–139.7) | 8381 (4114–12649) | 65.5 (32.3–98.8) | −67.2 (−128.4 to −6) |
| Tunisia | 5950 (4079–7821) | 76.7 (51.2–102.2) | 5635 (2652–8619) | 47.4 (22.1–72.7) | −67.5 (−115.8 to −19.1) |
| Turkey | 48545 (27045–70044) | 90.1 (55.7–124.4) | 35024 (27619–42428) | 43 (34.6–51.4) | −91.5 (−141.3 to −41.8) |
| United Arab Emirates | 2694 (1853–3535) | 248.5 (168.5–328.5) | 6645 (5062–8227) | 145.6 (114.1–177) | −97.9 (−131.7 to −64.1) |
| Yemen | 12646 (4011–21281) | 85 (41.3–128.7) | 19201 (11442–26961) | 70.4 (37.4–103.4) | −54.7 (−116.1 to 6.8) |
| Oceania | 7449 (4418–10479) | 139.2 (85.7–192.8) | 18289 (11464–25114) | 147.5 (92–203) | −20.3 (−66.3 to 25.8) |
| American Samoa | 85 (68–102) | 240.8 (190.5–291.1) | 111 (87–135) | 231.7 (181.9–281.6) | −56.2 (−99.5 to −12.9) |
| Cook Islands | 3 (2–4) | 18.8 (14.3–23.3) | 3 (2–3) | 13.5 (9.8–17.2) | −94.4 (−136.4 to −52.4) |
| Fiji | 585 (455–715) | 102.3 (80.4–124.1) | 928 (668–1189) | 108 (78.8–137.3) | −14.5 (−68.9 to 39.9) |
| Guam | 144 (119–169) | 130.5 (108.4–152.6) | 155 (131–179) | 90.3 (76.2–104.4) | −65 (−95.4 to −34.6) |
| Kiribati | 77 (34–120) | 136.8 (73.6–200) | 124 (72–177) | 132 (78.1–186) | −9 (−72.6 to 54.6) |
| Marshall Islands | 43 (25–60) | 153.1 (92.6–213.6) | 68 (38–98) | 144 (84.2–203.8) | −34.2 (−87.5 to 19.2) |
| Micronesia (Federated States of) | 128 (75–181) | 179.6 (109.1–250.1) | 131 (85–178) | 149.5 (98.1–201) | −49.3 (−100.7 to 2.1) |
| Nauru | 15 (9–21) | 197 (127.9–266.1) | 16 (10–23) | 201.2 (140.8–261.5) | −22.3 (−82.3 to 37.8) |
| Niue | 3 (2–4) | 144.2 (103–185.5) | 3 (2–4) | 197.4 (159.2–235.6) | 13.4 (−52 to 78.7) |
| Northern Mariana Islands | 41 (29–53) | 126.4 (92.9–160) | 69 (53–85) | 130.1 (103.5–156.6) | −38.7 (−100.3 to 22.8) |
| Palau | 14 (9–20) | 112.1 (72.6–151.7) | 19 (13–24) | 94.3 (66.5–122.2) | −43.6 (−95.3 to 8) |
| Papua New Guinea | 5169 (2715–7624) | 146.7 (79.8–213.6) | 14636 (8642–20630) | 157.2 (87.4–227.1) | −20.2 (−82.3 to 41.8) |
| Samoa | 179 (118–240) | 146.9 (94.2–199.5) | 225 (155–295) | 126.9 (89.2–164.6) | −45.2 (−93.6 to 3.3) |
| Solomon Islands | 239 (133–345) | 108.6 (55.9–161.4) | 538 (311–765) | 111 (63.6–158.3) | −16.9 (−78.3 to 44.5) |
| Tokelau | 2 (1–3) | 162.1 (99.1–225.1) | 3 (2–4) | 231 (159.7–302.3) | 20.2 (−58.1 to 98.5) |
| Tonga | 73 (55–92) | 98 (71.6–124.3) | 78 (56–100) | 83.6 (61–106.1) | −44 (−95.7 to 7.7) |
| Tuvalu | 15 (8–23) | 175 (103.1–246.9) | 16 (11–21) | 140.2 (97.7–182.8) | −56.5 (−107.1 to −5.8) |
| Vanuatu | 156 (87–225) | 139.1 (78.5–199.7) | 344 (202–486) | 135.1 (79.8–190.4) | −30.3 (−82 to 21.5) |
| South Asia | 1046248 (613894–1478602) | 133.2 (76.8–189.5) | 2038331 (1565072–2511590) | 130.9 (100.7–161) | −19.5 (−70 to 31) |
| Bangladesh | 111121 (52284–169959) | 168.7 (77.5–260) | 210234 (127434–293035) | 149.1 (91.4–206.9) | −42.4 (−109.2 to 24.5) |
| Bhutan | 490 (210–770) | 136.1 (46.5–225.7) | 877 (498–1255) | 136.1 (79.7–192.5) | −16.9 (−103.9 to 70.2) |
| India | 790233 (468216–1112251) | 126.4 (75.3–177.5) | 1523783 (1177094–1870473) | 123.6 (95.7–151.5) | −19.5 (−71.2 to 32.2) |
| Nepal | 19890 (9528–30252) | 146.9 (64.8–229) | 32816 (18663–46968) | 132.6 (77.7–187.4) | −33.5 (−108.3 to 41.3) |
| Pakistan | 124514 (67051–181976) | 144.7 (74.7–214.6) | 270620 (185664–355576) | 159.9 (108–211.7) | 6.4 (−61.3 to 74.1) |
| Southeast Asia | 253197 (200021–306373) | 85.4 (67.6–103.2) | 474713 (391648–557777) | 73.7 (61.8–85.6) | −20.6 (−63.8 to 22.6) |
| Cambodia | 3951 (2036–5866) | 71.4 (40–102.7) | 8299 (5303–11295) | 66.1 (43.9–88.4) | −11.6 (−87.1 to 63.9) |
| Indonesia | 89234 (60414–118054) | 76.5 (49.8–103.2) | 194615 (136763–252467) | 86.3 (63.6–109) | 19.9 (−43.1 to 82.9) |
| Lao People's Democratic Republic | 2556 (888–4224) | 101.8 (52.4–151.2) | 4493 (2883–6102) | 84.7 (56.1–113.3) | −37.6 (−105.6 to 30.4) |
| Malaysia | 15024 (12277–17772) | 124.7 (98.9–150.5) | 22515 (18876–26154) | 77.6 (65.4–89.8) | −90.1 (−142.8 to −37.4) |
| Maldives | 99 (38–160) | 81.5 (47.9–115.1) | 193 (129–257) | 51.2 (38.9–63.4) | −86.3 (−143.1 to −29.5) |
| Mauritius | 377 (354–401) | 44.6 (41.9–47.3) | 1921 (1776–2067) | 121.1 (112.3–130) | 205.9 (169.2–242.6) |
| Myanmar | 28521 (15218–41824) | 107.7 (65.4–150.1) | 43600 (30459–56741) | 90.1 (65.1–115.1) | −32.2 (−98.9 to 34.6) |
| Philippines | 43245 (35502–50988) | 126.5 (96.3–156.6) | 88586 (70705–106466) | 99.2 (78.5–119.9) | −35.1 (−68.3 to −1.9) |
| Seychelles | 155 (134–176) | 258.5 (222.5–294.6) | 158 (125–191) | 140.7 (112.5–168.9) | −64.8 (−109.7 to −20) |
| Sri Lanka | 29240 (21929–36552) | 235.9 (176.7–295) | 16413 (8001–24825) | 66.3 (34–98.5) | −133 (−170.3 to −95.6) |
| Thailand | 5470 (3324–7617) | 12.5 (6.7–18.2) | 19259 (14153–24365) | 22.2 (15.9–28.4) | 180.3 (−26.8 to 387.5) |
| Timor-Leste | 366 (183–550) | 87 (45.3–128.6) | 848 (536–1161) | 89.3 (57.2–121.5) | 6.6 (−77 to 90.2) |
| Viet Nam | 34590 (23090–46090) | 80.3 (52.4–108.1) | 73150 (52920–93380) | 77.9 (57.6–98.3) | 12.3 (−86.4 to 111) |
| Southern Latin America | 165960 (153648–178272) | 359.5 (332.2–386.8) | 154447 (143937–164956) | 186.3 (173.7–198.9) | −127.8 (−133.8 to −121.9) |
| Uruguay | 10553 (9749–11357) | 288.3 (266.8–309.8) | 6487 (6017–6958) | 130.7 (121.3–140.1) | −115.7 (−125 to −106.5) |
| Argentina | 137595 (125842–149348) | 433.4 (396.2–470.7) | 131473 (121710–141236) | 243.8 (226.3–261.4) | −127.9 (−134.6 to −121.2) |
| Chile | 17804 (16961–18648) | 159.8 (152.1–167.5) | 16478 (15376–17580) | 71.2 (66.4–76) | −117.7 (−125.7 to −109.7) |
| Southern Sub-Saharan Africa | 112147 (96350–127944) | 341.4 (277.6–405.1) | 188419 (167319–209520) | 301.9 (268.2–335.7) | −29.6 (−69 to 9.8) |
| Botswana | 2536 (1810–3261) | 368 (263.4–472.6) | 4245 (3020–5471) | 260.9 (189.2–332.5) | −66.9 (−103.3 to −30.5) |
| Eswatini | 1371 (971–1771) | 349.1 (249–449.2) | 1954 (1317–2592) | 272.4 (180–364.8) | −54.1 (−92.4 to −15.7) |
| Lesotho | 2616 (1663–3568) | 283.7 (175.2–392.3) | 3488 (2234–4741) | 289.3 (184.3–394.3) | −10.3 (−66 to 45.4) |
| Namibia | 2693 (2077–3309) | 357.2 (276.8–437.6) | 4597 (3299–5894) | 299.3 (220–378.7) | −42.1 (−79.6 to −4.7) |
| South Africa | 87754 (75202–100307) | 349.7 (282.1–417.4) | 142603 (124513–160693) | 295 (258.5–331.4) | −59.5 (−80.4 to −38.7) |
| Zimbabwe | 15177 (12189–18165) | 320.4 (249–391.8) | 31533 (23241–39825) | 364.9 (270.5–459.4) | 30.5 (−48.6 to 109.5) |
| Tropical Latin America | 418326 (403123–433528) | 394.2 (379.3–409.1) | 484333 (459529–509137) | 193.8 (183.9–203.7) | −118.2 (−122.5 to −113.9) |
| Brazil | 413944 (398974–428913) | 400.6 (385.5–415.8) | 478236 (454012–502459) | 196.4 (186.3–206.5) | −118.4 (−122.7 to −114.1) |
| Paraguay | 4382 (3634–5130) | 154.1 (126.6–181.7) | 6097 (4670–7524) | 98.9 (75.7–122) | −77.4 (−119.2 to −35.6) |
| Western Europe | 1055547 (999575–1111520) | 201.5 (191.3–211.7) | 635348 (586453–684243) | 77.8 (72.6–83) | −120.9 (−126.9 to −115) |
| Andorra | 98 (70–125) | 192.5 (138.6–246.5) | 154 (112–196) | 104.3 (77.3–131.3) | −94.5 (−131.6 to −57.4) |
| Austria | 71018 (66581–75455) | 636.4 (600.6–672.1) | 23688 (21457–25919) | 134.9 (125.1–144.7) | −156.5 (−159.5 to −153.5) |
| Belgium | 23972 (22374–25570) | 171.2 (160.5–181.9) | 13946 (12502–15390) | 59.4 (54.4–64.5) | −87.8 (−98.6 to −77) |
| Cyprus | 913 (660–1165) | 141.1 (99.6–182.6) | 1156 (896–1415) | 64.7 (50.9–78.6) | −108.7 (−140 to −77.5) |
| Denmark | 4702 (4359–5045) | 69.5 (64.7–74.4) | 3205 (2925–3486) | 30.3 (27.8–32.8) | −108.3 (−119.5 to −97.2) |
| Finland | 12537 (11284–13789) | 203 (182.7–223.3) | 11345 (10491–12199) | 117 (108.6–125.4) | −85 (−100.6 to −69.5) |
| France | 126827 (119648–134005) | 164.2 (155.4–173) | 92720 (83644–101796) | 70.6 (63.3–77.9) | −77.6 (−90.4 to −64.9) |
| Germany | 258619 (234011–283227) | 232.5 (211.1–254) | 173082 (158021–188143) | 103.6 (96.2–110.9) | −107.9 (−119.6 to −96.2) |
| Greece | 14497 (13579–15415) | 112 (105.2–118.7) | 14976 (13768–16184) | 81.9 (75.8–88.1) | −47.1 (−69 to −25.2) |
| Iceland | 190 (177–202) | 68.7 (64.3–73.1) | 144 (127–160) | 26.7 (23.9–29.4) | −113.6 (−123.3 to −103.9) |
| Ireland | 9774 (9266–10282) | 261.5 (247.9–275.2) | 5729 (5112–6346) | 77.4 (69.7–85.1) | −129.5 (−137.7 to −121.3) |
| Israel | 3740 (3454–4025) | 79.7 (73.8–85.5) | 2710 (2431–2989) | 22.9 (20.5–25.3) | −113.6 (−126 to −101.2) |
| Italy | 301689 (278219–325158) | 367.2 (337.4–396.9) | 78989 (68707–89271) | 59 (51.3–66.7) | −108.4 (−122.5 to −94.3) |
| Luxembourg | 935 (884–987) | 193.8 (183.4–204.2) | 540 (487–592) | 51.5 (46.5–56.5) | −137.2 (−144.6 to −129.8) |
| Malta | 957 (897–1016) | 247.3 (231.2–263.3) | 400 (355–445) | 45.9 (41–50.9) | −143.4 (−151.2 to −135.6) |
| Monaco | 180 (132–228) | 293.9 (221.7–366.1) | 131 (96–166) | 152.7 (111.6–193.7) | −95.5 (−126.4 to −64.7) |
| Netherlands | 35463 (33422–37503) | 192.8 (182.4–203.2) | 18217 (16475–19958) | 57.5 (52.4–62.6) | −138.2 (−143.2 to −133.1) |
| Norway | 3244 (3110–3377) | 69.2 (66.3–72.2) | 3827 (3557–4098) | 48 (44.6–51.4) | −80.4 (−86.9 to −73.8) |
| Portugal | 11596 (11009–12182) | 102.2 (97.1–107.2) | 12358 (11141–13576) | 59.2 (53.5–64.8) | −67.2 (−81.9 to −52.6) |
| San Marino | 59 (46–72) | 181.5 (142.8–220.2) | 50 (33–66) | 66.4 (44.9–87.9) | −111.7 (−146.2 to −77.2) |
| Spain | 96755 (90883–102627) | 202.5 (190.8–214.2) | 89936 (81206–98666) | 102.2 (92.6–111.9) | −89.4 (−100.3 to −78.6) |
| Sweden | 12818 (12146–13489) | 117.9 (110.8–125) | 10765 (9636–11895) | 67 (59.6–74.5) | 6.6 (−24.4 to 37.5) |
| Switzerland | 10170 (9243–11096) | 112.3 (102.7–121.9) | 6566 (5837–7295) | 38.3 (34.2–42.3) | −129.7 (−141.4 to −118.1) |
| United Kingdom | 53930 (52485–55375) | 78.7 (76.5–81) | 70155 (66091–74219) | 69.4 (65.9–73) | −15.9 (−23.3 to −8.4) |
| Western Sub-Saharan Africa | 449804 (326576–573032) | 347.6 (244.5–450.6) | 704112 (533489–874736) | 225.3 (177.5–273.1) | −92.4 (−119.7 to −65) |
| Benin | 8498 (5565–11432) | 272.6 (169.6–375.7) | 15643 (11154–20132) | 187.3 (132.5–242) | −80.5 (−126.3 to −34.6) |
| Burkina Faso | 22102 (12462–31741) | 374.8 (196.3–553.2) | 36197 (24849–47545) | 277.3 (175.4–379.2) | −79.4 (−126.9 to −31.9) |
| Cabo Verde | 326 (231–421) | 115.4 (77.7–153.2) | 457 (346–568) | 91.8 (68.8–114.9) | −24.8 (−85.6 to 36) |
| Cameroon | 21637 (14168–29106) | 335.4 (228.3–442.4) | 45752 (31419–60085) | 233.1 (162.2–303.9) | −77.5 (−127.2 to −27.7) |
| Chad | 12692 (7126–18259) | 314.8 (151.9–477.7) | 25653 (15985–35322) | 251.8 (148–355.6) | −62.3 (−113.9 to −10.7) |
| Côte d'Ivoire | 24757 (16557–32957) | 346.7 (231.3–462.1) | 42829 (29056–56603) | 239.5 (165–314) | −85.5 (−129.2 to −41.8) |
| Gambia | 1867 (1184–2550) | 335.7 (200.2–471.2) | 3572 (2528–4616) | 246.7 (178.7–314.7) | −80.4 (−128.1 to −32.7) |
| Ghana | 40664 (32108–49220) | 434.7 (322.7–546.8) | 87753 (59849–115657) | 396.2 (275–517.3) | −52.5 (−109.5 to 4.5) |
| Guinea | 15853 (10221–21484) | 333.1 (201.9–464.2) | 21842 (14616–29069) | 251.4 (168.1–334.6) | −66 (−119.4 to −12.5) |
| Guinea-Bissau | 2589 (1475–3703) | 410.6 (245.2–575.9) | 3385 (2385–4386) | 276.9 (195.4–358.4) | −88.2 (−135.1 to −41.4) |
| Liberia | 6784 (4359–9208) | 365.7 (219.5–512) | 7406 (4612–10200) | 224 (140.8–307.1) | −89.4 (−140.1 to −38.8) |
| Mali | 16639 (9762–23515) | 275.4 (155.6–395.2) | 28912 (19173–38651) | 191.3 (122.3–260.3) | −80.7 (−125.9 to −35.5) |
| Mauritania | 4367 (3051–5682) | 329.3 (229.1–429.4) | 5671 (3888–7454) | 204.4 (140.8–268.1) | −94.1 (−138.6 to −49.7) |
| Niger | 15251 (8205–22297) | 316.7 (156.4–477) | 28781 (15165–42398) | 215.3 (109.4–321.3) | −87.9 (−129.3 to −46.6) |
| Nigeria | 218533 (149171–287895) | 348 (234.5–461.6) | 297712 (199818–395605) | 188 (132–244) | −115.6 (−146.2 to −84.9) |
| Sao Tome and Principe | 220 (145–294) | 254.8 (163.9–345.7) | 283 (178–388) | 192.8 (133.4–252.3) | −65.3 (−121.5 to −9) |
| Senegal | 17603 (11041–24165) | 351.5 (212.1–490.9) | 24743 (17001–32485) | 236.8 (163.1–310.5) | −89.9 (−137.6 to −42.1) |
| Sierra Leone | 12311 (7256–17366) | 373.3 (225.7–520.8) | 15117 (10096–20138) | 248.7 (161.9–335.6) | −88.7 (−137.7 to −39.7) |
| Togo | 7099 (4795–9402) | 338.3 (219.9–456.7) | 12395 (8093–16697) | 233.2 (154.9–311.4) | −76.8 (−127.4 to −26.3) |
