# Supplementary material for: Global, regional, and national burden of cardiomyopathy (including alcoholic cardiomyopathy and others) from 1990 to 2021: An analysis of data from the global burden of disease study 2021 and forecast to 2040
Source: PLoS One. 2026 Jan 30;21(1):e0341687. doi: 10.1371/journal.pone.0341687 (PMC12858021; doi:10.1371/journal.pone.0341687)
Supplement: S10 Table — (DOCX) [file pone.0341687.s021.docx]

**S10 Table.** **1990–2021 Global and national prevalence trends in alcoholic cardiomyopathy burden.**

| location_name | Number_1990 | ASR per 100,000_1990 | Number_2021 | ASR per 100,000_2021 | Percentage change in the ASRs per 100,000 |
| --- | --- | --- | --- | --- | --- |
| Global | 356476 (301779–421492) | 7.9 (6.7–9.4) | 528429 (439582–639167) | 6.2 (5.1–7.4) | −22.5 (−26.6 to −18.2) |
| Andean Latin America | 8 (6–11) | 0 (0–0.1) | 28 (20–38) | 0 (0–0.1) | 11.1 (−5.1 to 29.4) |
| Bolivia (Plurinational State of) | 1 (1–2) | 0 (0–0.1) | 5 (3–6) | 0.1 (0–0.1) | 30.9 (10–54.4) |
| Ecuador | 2 (1–2) | 0 (0–0) | 4 (3–6) | 0 (0–0) | −3.7 (−21.7 to 18.2) |
| Peru | 6 (4–8) | 0.1 (0–0.1) | 19 (14–25) | 0.1 (0–0.1) | 12.1 (−7.3 to 35.5) |
| Australasia | 3031 (2483–3675) | 13.4 (11–16.3) | 11896 (9653–14308) | 26.6 (21.8–31.8) | 98.5 (76.7–123.2) |
| Australia | 2519 (2047–3071) | 13.4 (10.9–16.3) | 10681 (8566–12819) | 28.5 (23.1–34.3) | 113.7 (87.2–142.6) |
| New Zealand | 513 (412–641) | 13.7 (11–16.8) | 1215 (966–1549) | 16.7 (13.6–20.8) | 22.1 (7.9–37.9) |
| Caribbean | 1385 (1141–1684) | 4.6 (3.8–5.6) | 9445 (7742–11510) | 18 (14.8–21.9) | 291.8 (259.7–324.1) |
| Antigua and Barbuda | 1 (1–1) | 1.2 (1–1.4) | 9 (7–11) | 7.9 (6.4–9.7) | 574.6 (482.4–674.2) |
| Bahamas | 14 (11–17) | 6.5 (5.4–7.8) | 75 (60–91) | 17.5 (14.1–21) | 171.1 (143.6–205.4) |
| Barbados | 44 (37–53) | 16.7 (13.8–20.3) | 139 (112–174) | 32 (26.3–39.1) | 91 (69.5–111.8) |
| Belize | 4 (3–5) | 3.3 (2.7–4) | 64 (52–79) | 17 (13.8–20.8) | 416.9 (356.8–489.5) |
| Bermuda | 3 (2–3) | 3.9 (3.2–4.8) | 30 (23–37) | 28.7 (23.1–35.3) | 637.7 (555.5–726.9) |
| Cuba | 519 (420–636) | 4.7 (3.8–5.8) | 6055 (4902–7423) | 38.3 (31.6–46.3) | 714 (627–807.1) |
| Dominica | 2 (2–3) | 4.1 (3.3–5) | 4 (3–6) | 5.3 (4.3–6.7) | 29.8 (13.5–47.5) |
| Dominican Republic | 161 (132–192) | 3.2 (2.6–3.8) | 735 (587–911) | 6.7 (5.4–8.3) | 112.3 (87.2–137) |
| Grenada | 8 (6–9) | 11 (8.9–13.3) | 26 (21–32) | 22.1 (18–27.2) | 101.4 (78.9–124.5) |
| Guyana | 20 (16–24) | 3.4 (2.8–4.2) | 85 (68–104) | 11.6 (9.3–14.2) | 236.6 (201–276.4) |
| Haiti | 266 (217–330) | 6.4 (5.2–7.9) | 841 (684–1040) | 8.6 (7–10.5) | 33 (19.9–47.8) |
| Jamaica | 129 (103–156) | 6.8 (5.5–8.4) | 341 (279–423) | 11.1 (9.1–13.8) | 63.8 (45.7–87.8) |
| Puerto Rico | 118 (98–145) | 3.2 (2.7–3.9) | 431 (338–538) | 8.4 (6.8–10.2) | 159.9 (133.2–189.6) |
| Saint Kitts and Nevis | 1 (1–1) | 2.7 (2.2–3.3) | 11 (9–13) | 15.1 (12.4–18.3) | 459 (402.4–528) |
| Saint Lucia | 8 (7–10) | 8.3 (6.8–10.1) | 84 (68–103) | 36.9 (30.1–45) | 346 (302.9–401.4) |
| Saint Vincent and the Grenadines | 4 (4–5) | 5.2 (4.3–6.2) | 20 (17–25) | 15.3 (12.6–18.5) | 197.6 (162.5–235.8) |
| Suriname | 7 (6–9) | 2.4 (1.9–2.9) | 34 (27–42) | 5.4 (4.3–6.6) | 124.5 (99–151.7) |
| Trinidad and Tobago | 23 (18–27) | 2.3 (1.9–2.8) | 124 (98–156) | 6.9 (5.5–8.6) | 200.6 (167–237.9) |
| United States Virgin Islands | 7 (6–9) | 7 (5.8–8.6) | 17 (13–21) | 12.6 (10.4–15.4) | 79.5 (64.5–97.6) |
| Central Asia | 2202 (1778–2698) | 3.9 (3.2–4.8) | 5182 (4163–6386) | 5.4 (4.4–6.6) | 39 (28.5–48.8) |
| Armenia | 839 (676–1013) | 27 (22.1–32.5) | 619 (504–756) | 17.4 (14.3–21.3) | −35.5 (−42 to −28.2) |
| Azerbaijan | 119 (95–150) | 1.9 (1.5–2.4) | 214 (168–270) | 1.8 (1.4–2.2) | −6.5 (−15.9 to 5.4) |
| Georgia | 127 (101–164) | 2.1 (1.7–2.7) | 95 (70–124) | 2.1 (1.6–2.8) | 0.6 (−14.1 to 17.2) |
| Kazakhstan | 75 (59–93) | 0.5 (0.4–0.6) | 690 (530–890) | 3.5 (2.7–4.5) | 611.1 (506.8–754.6) |
| Kyrgyzstan | 723 (581–890) | 20.4 (16.4–25.2) | 2798 (2233–3433) | 44.7 (36–54.6) | 119.4 (98.6–141.7) |
| Mongolia | 234 (186–289) | 15.5 (12.4–19.1) | 630 (480–805) | 19.9 (15.4–25.1) | 28.1 (13.3–44.8) |
| Tajikistan | 1 (0–1) | 0 (0–0) | 1 (1–2) | 0 (0–0) | 28.1 (14.5–44) |
| Turkmenistan | 78 (62–98) | 3.1 (2.5–3.9) | 123 (96–156) | 2.5 (2–3.1) | −20.4 (−29.2 to −11.3) |
| Uzbekistan | 6 (5–7) | 0 (0–0) | 12 (9–15) | 0 (0–0) | −8.7 (−18 to 2.8) |
| Central Europe | 21465 (17903–25571) | 15.6 (13.1–18.5) | 42372 (35272–50685) | 25 (21.1–29.5) | 60.4 (47–75) |
| Albania | 199 (164–243) | 7.9 (6.4–9.6) | 381 (299–482) | 10.7 (8.4–13.4) | 35.6 (20–51.4) |
| Bosnia and Herzegovina | 662 (545–816) | 14.8 (12.3–18.2) | 860 (667–1068) | 18.1 (14.3–22.3) | 22.1 (8.7–38) |
| Bulgaria | 114 (91–140) | 1.1 (0.9–1.3) | 127 (99–159) | 1.3 (1.1–1.6) | 22.2 (7.6–38.4) |
| Croatia | 689 (561–834) | 12.5 (10.2–15.1) | 1507 (1081–1916) | 22.8 (17.7–27.8) | 82.7 (53.3–110.3) |
| Czechia | 330 (269–398) | 2.8 (2.3–3.4) | 1756 (1378–2211) | 12.1 (9.6–15.2) | 327.1 (277.1–386.8) |
| Hungary | 4345 (3435–5426) | 34.3 (28–42.3) | 6694 (5170–8512) | 46.6 (37.6–58.4) | 35.7 (21.6–51.5) |
| Montenegro | 199 (163–241) | 31.4 (25.9–37.8) | 243 (194–303) | 28.8 (23.1–35.3) | −8 (−18.1 to 2.6) |
| North Macedonia | 310 (256–373) | 15.7 (12.9–18.8) | 482 (376–604) | 16.8 (13.3–20.6) | 6.5 (−5.1 to 19.1) |
| Poland | 8853 (7443–10494) | 21.7 (18.4–25.7) | 21882 (18304–26044) | 38.4 (32.5–45.4) | 76.7 (56.4–100.3) |
| Romania | 3077 (2381–3892) | 11.8 (9.2–14.9) | 3541 (2718–4549) | 13.3 (10.7–16.6) | 12.8 (−1.4 to 28.9) |
| Serbia | 1526 (1218–1915) | 14 (11.4–17.3) | 2321 (1790–2887) | 18.9 (15.1–23.2) | 35 (21.8–52) |
| Slovakia | 310 (251–375) | 5.6 (4.5–6.8) | 1093 (854–1396) | 14.7 (11.5–18.6) | 163.5 (131.1–200.3) |
| Slovenia | 507 (400–635) | 21.9 (17.5–27.3) | 869 (671–1086) | 26.7 (21.5–32.5) | 21.8 (8.5–35.5) |
| Central Latin America | 3427 (2844–4077) | 2.7 (2.3–3.1) | 7976 (6595–9617) | 3 (2.5–3.6) | 12.7 (5.2–20.6) |
| Colombia | 178 (143–220) | 0.6 (0.5–0.8) | 980 (797–1214) | 1.8 (1.5–2.3) | 181.4 (154.7–216.5) |
| Costa Rica | 131 (105–160) | 5.5 (4.5–6.8) | 374 (304–457) | 7 (5.7–8.5) | 27 (14.6–41.4) |
| El Salvador | 24 (19–29) | 0.6 (0.5–0.8) | 35 (29–43) | 0.6 (0.4–0.7) | −11.3 (−21.5 to −0.3) |
| Guatemala | 99 (79–125) | 1.9 (1.5–2.3) | 230 (184–285) | 1.6 (1.3–2) | −11.4 (−22.6 to 1.3) |
| Honduras | 55 (44–67) | 2 (1.6–2.3) | 138 (110–174) | 1.6 (1.3–2) | −16.5 (−25.1 to −5.8) |
| Mexico | 1949 (1603–2354) | 2.8 (2.4–3.4) | 4813 (3903–5878) | 3.5 (2.9–4.3) | 23.6 (14.4–33.3) |
| Nicaragua | 55 (44–68) | 2.2 (1.8–2.7) | 123 (99–152) | 2 (1.6–2.5) | −11 (−21.6 to −1.7) |
| Panama | 57 (46–69) | 2.9 (2.4–3.6) | 131 (107–157) | 3 (2.4–3.6) | 1.3 (−8.8 to 12.3) |
| Venezuela (Bolivarian Republic of) | 879 (708–1080) | 6.1 (5–7.4) | 1150 (941–1445) | 4 (3.3–5) | −33.4 (−41.4 to −24) |
| Central Sub-Saharan Africa | 658 (477–888) | 1.9 (1.4–2.5) | 1485 (1093–2035) | 1.6 (1.2–2.2) | −12.8 (−20.7 to −4) |
| Angola | 132 (94–181) | 2 (1.5–2.7) | 312 (226–425) | 1.6 (1.2–2.1) | −20.6 (−30.7 to −10.3) |
| Central African Republic | 32 (23–46) | 1.7 (1.3–2.4) | 56 (41–78) | 1.5 (1.1–2) | −14.1 (−24.1 to −3.1) |
| Congo | 21 (15–28) | 1.4 (1–1.8) | 43 (32–58) | 1.1 (0.8–1.4) | −22.1 (−30.8 to −12.8) |
| Democratic Republic of the Congo | 456 (328–618) | 1.9 (1.4–2.5) | 1054 (768–1454) | 1.7 (1.3–2.3) | −8.4 (−18.4 to 2.7) |
| Equatorial Guinea | 5 (3–6) | 1.7 (1.3–2.2) | 8 (6–11) | 0.8 (0.6–1.1) | −50 (−55.4 to −42.7) |
| Gabon | 12 (9–16) | 1.7 (1.2–2.2) | 12 (9–16) | 0.9 (0.7–1.2) | −47.8 (−54 to −39.9) |
| East Asia | 7047 (5871–8429) | 0.6 (0.5–0.7) | 30311 (24089–37517) | 1.6 (1.3–2) | 158.1 (133–183.6) |
| China | 5634 (4658–6799) | 0.5 (0.4–0.6) | 28103 (22175–34991) | 1.6 (1.2–1.9) | 200.4 (171.3–230.5) |
| Democratic People's Republic of Korea | 203 (163–254) | 1 (0.8–1.3) | 434 (348–529) | 1.4 (1.2–1.7) | 36.4 (22.5–52) |
| Taiwan (Province of China) | 1211 (983–1468) | 6.3 (5.2–7.6) | 1774 (1411–2145) | 5.8 (4.6–7.1) | −8.5 (−17.9 to 1.4) |
| Eastern Europe | 142047 (119221–168870) | 56.5 (47.8–66.8) | 173043 (142599–211309) | 62.5 (51.9–75) | 10.5 (3.3–17.8) |
| Belarus | 5354 (4294–6681) | 46.5 (37.3–57.7) | 5336 (4092–6692) | 46.1 (36.4–57.6) | −0.8 (−11.7 to 8.8) |
| Estonia | 606 (482–757) | 34.5 (27.3–42.8) | 587 (458–750) | 33.5 (26.7–42.8) | −3 (−16.1 to 11.1) |
| Latvia | 1177 (945–1444) | 38.5 (31.1–46.8) | 1634 (1280–2057) | 62.2 (49.6–77.1) | 61.5 (43.4–81.1) |
| Lithuania | 1102 (870–1367) | 27.3 (21.6–34) | 1392 (1085–1770) | 37.2 (29.1–47) | 36.1 (19.8–55.6) |
| Republic of Moldova | 612 (479–760) | 13.5 (10.6–16.9) | 1459 (1108–1891) | 30 (22.8–39.1) | 121.9 (92.2–157) |
| Russian Federation | 95037 (80093–112435) | 57.4 (48.4–67.5) | 128321 (105674–158267) | 65.7 (54.6–79.2) | 14.5 (5.4–23) |
| Ukraine | 38159 (31765–45602) | 63 (53.4–75.4) | 34314 (28495–41693) | 60.9 (50.9–73.2) | −3.3 (−10.8 to 4.9) |
| Eastern Sub-Saharan Africa | 3349 (2436–4504) | 2.6 (2–3.5) | 8012 (5775–10964) | 2.5 (1.9–3.4) | −3.2 (−8.1 to 2.8) |
| Burundi | 98 (69–137) | 2.6 (1.9–3.5) | 245 (173–335) | 2.7 (2–3.7) | 5 (−6.3 to 20.3) |
| Comoros | 8 (6–12) | 2.6 (1.9–3.5) | 16 (11–22) | 2.3 (1.7–3.1) | −9 (−18.7 to 1.6) |
| Djibouti | 8 (6–12) | 2.7 (2–3.7) | 28 (19–38) | 2.6 (1.9–3.5) | −5.5 (−16.7 to 7.6) |
| Eritrea | 54 (37–77) | 2.2 (1.6–3) | 116 (79–163) | 2.2 (1.5–3) | −2 (−14.4 to 11.6) |
| Ethiopia | 966 (710–1296) | 2.8 (2.2–3.7) | 2494 (1796–3373) | 3 (2.3–4) | 6.5 (−0.1 to 13.6) |
| Kenya | 377 (277–502) | 2.6 (2–3.3) | 937 (672–1238) | 2.3 (1.7–3.1) | −10.7 (−16.6 to −4.5) |
| Madagascar | 230 (163–319) | 2.8 (2.1–3.8) | 568 (400–772) | 2.6 (1.9–3.5) | −6.2 (−16.6 to 6.4) |
| Malawi | 151 (104–214) | 2.2 (1.6–3) | 303 (209–427) | 2.1 (1.5–2.9) | −5.2 (−15.5 to 6.7) |
| Mozambique | 217 (159–301) | 2.4 (1.8–3.2) | 463 (333–642) | 2.2 (1.6–3) | −7.6 (−18.5 to 5) |
| Rwanda | 123 (86–170) | 2.5 (1.8–3.4) | 266 (185–371) | 2.5 (1.8–3.4) | −1.4 (−13 to 10.9) |
| Somalia | 122 (85–170) | 2.4 (1.8–3.2) | 319 (216–461) | 2.2 (1.6–2.9) | −9 (−19.1 to 1.6) |
| South Sudan | 117 (84–162) | 2.9 (2.1–3.9) | 167 (118–232) | 2.4 (1.8–3.3) | −14.6 (−23.6 to −3.4) |
| Uganda | 289 (199–397) | 2.6 (1.9–3.4) | 733 (506–1026) | 2.4 (1.8–3.3) | −5.9 (−18.1 to 6.9) |
| United Republic of Tanzania | 458 (325–630) | 2.6 (1.9–3.5) | 1018 (710–1392) | 2.3 (1.7–3.2) | −9.9 (−20.3 to 1.9) |
| Zambia | 126 (88–177) | 2.4 (1.7–3.2) | 332 (228–470) | 2.3 (1.7–3.3) | −1.6 (−12.3 to 12) |
| High-income Asia Pacific | 13835 (11204–17351) | 6.7 (5.5–8.4) | 13148 (10795–16171) | 5 (4–6.1) | −25.8 (−32.1 to −18.5) |
| Brunei Darussalam | 13 (10–16) | 6.4 (5.1–8.1) | 28 (22–36) | 5.4 (4.2–6.8) | −16.1 (−25.4 to −5.1) |
| Japan | 13534 (10932–16968) | 8.5 (6.9–10.6) | 12338 (10063–15174) | 7.1 (5.7–8.6) | −17.3 (−24.8 to −9.2) |
| Republic of Korea | 238 (187–300) | 0.6 (0.4–0.7) | 714 (574–894) | 0.9 (0.8–1.2) | 67.7 (43.3–96.5) |
| Singapore | 49 (39–64) | 1.5 (1.2–2) | 69 (55–88) | 0.9 (0.7–1.1) | −43.8 (−50.9 to −35.8) |
| High-income North America | 62598 (51831–75371) | 19.7 (16.4–23.8) | 89214 (74204–108044) | 17.4 (14.6–20.9) | −11.7 (−19.4 to −1.6) |
| Canada | 6256 (5069–7627) | 20.2 (16.4–24.5) | 12719 (10164–15833) | 23.6 (19.2–28.8) | 16.7 (1.9–32.1) |
| Greenland | 4 (3–5) | 6.4 (5.1–8.1) | 7 (6–9) | 10.6 (8.2–13.5) | 66.3 (45.5–92.7) |
| United States of America | 56337 (46782–67856) | 19.7 (16.3–23.9) | 76487 (62813–93683) | 16.7 (13.9–20) | −15.1 (−23.8 to −4) |
| North Africa and Middle East | 897 (751–1087) | 0.4 (0.3–0.5) | 2524 (2072–3153) | 0.4 (0.4–0.5) | 5.5 (−1.2 to 13) |
| Afghanistan | 27 (22–34) | 0.4 (0.3–0.5) | 64 (51–81) | 0.4 (0.3–0.5) | −6 (−17 to 5.8) |
| Algeria | 105 (85–128) | 0.7 (0.6–0.8) | 309 (251–388) | 0.7 (0.6–0.9) | 6.2 (−4.5 to 18) |
| Bahrain | 5 (4–7) | 1.6 (1.3–1.9) | 30 (24–39) | 1.8 (1.5–2.3) | 14.5 (3.5–26.6) |
| Egypt | 6 (5–8) | 0 (0–0) | 17 (13–22) | 0 (0–0) | 27.2 (10.5–45.9) |
| Iran (Islamic Republic of) | 249 (204–302) | 0.7 (0.6–0.8) | 736 (572–930) | 0.8 (0.6–1) | 12.5 (3.5–21.5) |
| Iraq | 4 (4–6) | 0 (0–0.1) | 12 (9–15) | 0 (0–0) | −13.7 (−23.4 to −3.1) |
| Jordan | 1 (1–2) | 0.1 (0.1–0.1) | 6 (5–7) | 0.1 (0–0.1) | −16 (−25 to −5.5) |
| Kuwait | 35 (27–43) | 2.7 (2.1–3.3) | 62 (49–79) | 1.2 (1–1.5) | −53.8 (−59.5 to −47.2) |
| Lebanon | 1 (1–2) | 0.1 (0–0.1) | 4 (3–5) | 0.1 (0.1–0.1) | 18.8 (5.2–35.7) |
| Libya | 19 (15–24) | 0.8 (0.6–0.9) | 36 (28–46) | 0.5 (0.4–0.6) | −35.6 (−44.5 to −26.9) |
| Morocco | 101 (83–124) | 0.6 (0.5–0.7) | 218 (175–274) | 0.6 (0.5–0.7) | −0.2 (−11.7 to 12.9) |
| Oman | 2 (2–3) | 0.2 (0.1–0.2) | 6 (5–8) | 0.1 (0.1–0.2) | −19.3 (−31.9 to −6.8) |
| Palestine | 3 (3–4) | 0.3 (0.3–0.4) | 13 (10–16) | 0.4 (0.3–0.4) | 7 (−4.8 to 19.3) |
| Qatar | 2 (2–3) | 0.6 (0.5–0.7) | 30 (23–39) | 1 (0.8–1.3) | 66.4 (47.6–85.6) |
| Saudi Arabia | 48 (39–60) | 0.5 (0.4–0.6) | 148 (116–189) | 0.4 (0.3–0.5) | −25.8 (−32.8 to −17) |
| Sudan | 60 (49–73) | 0.5 (0.4–0.6) | 124 (100–155) | 0.4 (0.3–0.5) | −17.3 (−28 to −4.9) |
| Syrian Arab Republic | 36 (30–45) | 0.5 (0.4–0.7) | 80 (63–103) | 0.5 (0.4–0.7) | −1.7 (−13 to 11.8) |
| Tunisia | 55 (45–67) | 0.9 (0.8–1.1) | 123 (100–154) | 0.9 (0.8–1.1) | −0.5 (−10.5 to 11.9) |
| Turkey | 79 (64–98) | 0.2 (0.1–0.2) | 246 (197–317) | 0.3 (0.2–0.3) | 35.9 (19.2–55.9) |
| United Arab Emirates | 16 (12–20) | 1.1 (0.9–1.4) | 146 (108–193) | 1 (0.8–1.3) | −6.9 (−18 to 5.3) |
| Yemen | 39 (32–48) | 0.6 (0.5–0.7) | 112 (90–138) | 0.5 (0.4–0.6) | −12.9 (−22.9 to −2.6) |
| Oceania | 10 (8–13) | 0.2 (0.2–0.3) | 21 (17–26) | 0.2 (0.2–0.3) | −12.9 (−19.7 to −6.4) |
| American Samoa | 0 (0–1) | 1.2 (1–1.5) | 0 (0–0) | 0.6 (0.5–0.7) | −53.6 (−58.5 to −48.6) |
| Cook Islands | 0 (0–0) | 0 (0–0) | 0 (0–0) | 0 (0–0) | −10.1 (−27.7 to 8.6) |
| Fiji | 0 (0–0) | 0 (0–0) | 0 (0–0) | 0 (0–0.1) | 15.9 (5.2–28.3) |
| Guam | 1 (1–1) | 0.6 (0.5–0.7) | 1 (1–1) | 0.5 (0.4–0.6) | −21.7 (−30 to −12.8) |
| Kiribati | 0 (0–0) | 0.5 (0.4–0.6) | 0 (0–0) | 0.4 (0.4–0.5) | −4.4 (−13.9 to 6.1) |
| Marshall Islands | 0 (0–0) | 0.2 (0.2–0.2) | 0 (0–0) | 0.2 (0.1–0.2) | −16.5 (−23.8 to −8.9) |
| Micronesia (Federated States of) | 0 (0–0) | 0.2 (0.2–0.3) | 0 (0–0) | 0.2 (0.2–0.2) | −12.1 (−20.2 to −3.8) |
| Nauru | 0 (0–0) | 0.2 (0.1–0.2) | 0 (0–0) | 0.2 (0.1–0.2) | −11.1 (−20.3 to −0.9) |
| Niue | 0 (0–0) | 0.2 (0.2–0.3) | 0 (0–0) | 0.2 (0.1–0.2) | −19.6 (−26.9 to −11.7) |
| Northern Mariana Islands | 0 (0–1) | 1.3 (1.1–1.6) | 0 (0–0) | 0.6 (0.5–0.8) | −52.5 (−57.7 to −46.6) |
| Palau | 0 (0–0) | 0.2 (0.2–0.2) | 0 (0–0) | 0.1 (0.1–0.2) | −22.9 (−30 to −15.5) |
| Papua New Guinea | 6 (5–8) | 0.3 (0.2–0.3) | 16 (13–20) | 0.2 (0.2–0.3) | −10.2 (−18.9 to −2.4) |
| Samoa | 0 (0–0) | 0.3 (0.2–0.3) | 0 (0–0) | 0.2 (0.2–0.2) | −22 (−29.2 to −13.6) |
| Solomon Islands | 0 (0–0) | 0.2 (0.1–0.2) | 1 (1–1) | 0.2 (0.1–0.2) | −9.1 (−17.8 to 1.4) |
| Tokelau | 0 (0–0) | 0.3 (0.2–0.3) | 0 (0–0) | 0.2 (0.2–0.3) | −11.2 (−20.2 to −2.5) |
| Tonga | 0 (0–0) | 0.3 (0.3–0.4) | 0 (0–0) | 0.2 (0.2–0.3) | −24 (−31.2 to −15.8) |
| Tuvalu | 0 (0–0) | 0.2 (0.2–0.2) | 0 (0–0) | 0.2 (0.1–0.2) | −7.4 (−16.1 to 2.6) |
| Vanuatu | 0 (0–0) | 0.2 (0.2–0.2) | 0 (0–0) | 0.2 (0.1–0.2) | −20.8 (−28.5 to −13.5) |
| South Asia | 4590 (3835–5525) | 0.6 (0.5–0.8) | 11199 (9044–13682) | 0.7 (0.5–0.8) | 4.2 (−2.8 to 12.4) |
| Bangladesh | 460 (373–564) | 0.8 (0.6–1) | 1344 (1087–1640) | 0.9 (0.7–1.1) | 13 (0–25.4) |
| Bhutan | 2 (2–3) | 0.6 (0.5–0.8) | 6 (5–7) | 0.8 (0.6–1) | 24.8 (11.7–41.6) |
| India | 3631 (2992–4377) | 0.6 (0.5–0.7) | 8688 (6977–10725) | 0.6 (0.5–0.8) | 4.6 (−3.6 to 12.8) |
| Nepal | 79 (64–97) | 0.7 (0.5–0.8) | 179 (144–223) | 0.7 (0.5–0.8) | 0.3 (−11.8 to 14.6) |
| Pakistan | 418 (348–498) | 0.6 (0.5–0.8) | 982 (785–1207) | 0.6 (0.5–0.7) | −5 (−13.5 to 3.8) |
| Southeast Asia | 1210 (995–1466) | 0.4 (0.3–0.4) | 3219 (2644–3943) | 0.4 (0.4–0.5) | 22.9 (15.3–30.8) |
| Cambodia | 13 (10–16) | 0.2 (0.2–0.3) | 50 (39–63) | 0.3 (0.3–0.4) | 51.8 (36.9–69.4) |
| Indonesia | 420 (345–515) | 0.3 (0.3–0.4) | 971 (773–1209) | 0.3 (0.3–0.4) | 7.5 (−2 to 17.1) |
| Lao People's Democratic Republic | 5 (4–6) | 0.2 (0.2–0.2) | 17 (13–22) | 0.3 (0.2–0.3) | 44.6 (27.6–64.3) |
| Malaysia | 2 (1–2) | 0 (0–0) | 3 (2–4) | 0 (0–0) | −36.1 (−42.9 to −28.3) |
| Maldives | 1 (0–1) | 0.5 (0.4–0.6) | 4 (4–6) | 0.8 (0.7–1) | 70.2 (55.9–89.7) |
| Mauritius | 15 (12–19) | 1.6 (1.3–2) | 23 (18–28) | 1.3 (1.1–1.6) | −17.7 (−26.4 to −8.3) |
| Myanmar | 73 (57–89) | 0.3 (0.2–0.3) | 204 (157–255) | 0.4 (0.3–0.5) | 47.2 (30.1–66.7) |
| Philippines | 215 (175–264) | 0.5 (0.4–0.6) | 473 (381–586) | 0.5 (0.4–0.6) | −6.3 (−13.8 to 1.3) |
| Seychelles | 0 (0–0) | 0.2 (0.1–0.2) | 0 (0–0) | 0.3 (0.2–0.3) | 52.7 (33.4–70) |
| Sri Lanka | 156 (123–194) | 1.2 (0.9–1.4) | 210 (169–266) | 0.8 (0.7–1) | −28.3 (−36.3 to −18) |
| Thailand | 90 (72–111) | 0.2 (0.2–0.2) | 497 (392–614) | 0.5 (0.4–0.6) | 159.5 (135–191.4) |
| Timor-Leste | 2 (1–2) | 0.4 (0.3–0.4) | 4 (3–5) | 0.4 (0.3–0.5) | 11.1 (−1.3 to 25.5) |
| Viet Nam | 217 (177–271) | 0.5 (0.4–0.6) | 758 (591–967) | 0.7 (0.5–0.9) | 51.3 (35.4–71.6) |
| Southern Latin America | 3479 (2844–4230) | 7.4 (6–9) | 2105 (1705–2587) | 2.7 (2.2–3.3) | −63.9 (−67 to −59.8) |
| Uruguay | 713 (567–886) | 20.7 (16.6–25.6) | 477 (373–598) | 11 (8.8–13.6) | −47.1 (−52.8 to −40.2) |
| Argentina | 2249 (1808–2772) | 7 (5.6–8.7) | 889 (713–1104) | 1.7 (1.4–2.1) | −75.5 (−78.7 to −71.7) |
| Chile | 516 (419–631) | 4.3 (3.5–5.3) | 740 (604–930) | 3.3 (2.6–4.1) | −24.9 (−32.8 to −15.4) |
| Southern Sub-Saharan Africa | 381 (247–557) | 0.7 (0.5–1) | 452 (293–643) | 0.6 (0.4–0.8) | −23.3 (−28.1 to −17.8) |
| Botswana | 16 (10–23) | 1.5 (1.1–2.1) | 32 (22–47) | 1.3 (0.9–1.8) | −15.4 (−26 to −3.4) |
| Eswatini | 9 (6–13) | 1.4 (1–1.9) | 12 (8–18) | 1.1 (0.7–1.5) | −23.6 (−35 to −9.1) |
| Lesotho | 16 (11–24) | 1.3 (0.9–1.9) | 24 (15–34) | 1.2 (0.9–1.7) | −6.6 (−19.7 to 7) |
| Namibia | 18 (12–26) | 1.5 (1.1–2.1) | 33 (21–48) | 1.3 (0.9–1.9) | −13.5 (−25.3 to −1.7) |
| South Africa | 239 (148–358) | 0.6 (0.4–0.9) | 220 (142–323) | 0.4 (0.3–0.6) | −32.6 (−37.5 to −27.5) |
| Zimbabwe | 83 (56–116) | 1.1 (0.8–1.4) | 131 (86–187) | 1 (0.7–1.3) | −7.4 (−19.2 to 7.8) |
| Tropical Latin America | 15372 (12580–18309) | 11.9 (9.8–14.1) | 19237 (15574–23399) | 7.5 (6–9.1) | −37.1 (−42.2 to −31.9) |
| Brazil | 15275 (12496–18197) | 12.1 (10–14.4) | 19081 (15449–23224) | 7.6 (6.2–9.3) | −37 (−42.2 to −31.8) |
| Paraguay | 97 (77–120) | 3.1 (2.5–3.8) | 156 (122–195) | 2.2 (1.7–2.7) | −29.5 (−37.4 to −21.5) |
| Western Europe | 66147 (54656–78958) | 13.7 (11.5–16.3) | 88261 (71203–108293) | 13.4 (11–16.1) | −2.4 (−9.8 to 6) |
| Andorra | 10 (8–13) | 17.2 (14–20.8) | 22 (18–28) | 16.8 (13.4–20.8) | −2.3 (−12.8 to 9.1) |
| Austria | 4072 (3296–4742) | 37.1 (30.5–42.8) | 2144 (1762–2533) | 16.3 (13.5–19.1) | −56.2 (−62.8 to −46.2) |
| Belgium | 764 (615–942) | 6.1 (4.9–7.5) | 1463 (1197–1787) | 8.8 (7.2–10.7) | 43.1 (22.7–67) |
| Cyprus | 30 (24–36) | 3.6 (2.9–4.4) | 68 (54–85) | 3.8 (3.1–4.8) | 7.1 (−4.8 to 20) |
| Denmark | 411 (336–494) | 6.5 (5.3–7.9) | 782 (601–998) | 8.8 (6.9–11) | 36.1 (18.1–58.2) |
| Finland | 1511 (1224–1839) | 25.2 (20.4–30.6) | 2462 (1906–3144) | 29.7 (23.4–36.7) | 17.6 (2.2–34.8) |
| France | 8164 (6720–9897) | 11.7 (9.6–14.1) | 21419 (16704–27541) | 20.6 (16.2–26) | 75.2 (51.9–100.6) |
| Germany | 37003 (29767–45272) | 35.1 (28.6–42.6) | 34170 (26684–43110) | 25.6 (20.7–31.4) | −27.1 (−34.5 to −18.8) |
| Greece | 172 (137–215) | 1.3 (1.1–1.7) | 175 (144–208) | 1.2 (1–1.4) | −10.2 (−21.4 to 3.8) |
| Iceland | 8 (6–9) | 2.9 (2.3–3.6) | 14 (11–17) | 3.1 (2.4–3.9) | 6.2 (−7.8 to 21.6) |
| Ireland | 275 (226–331) | 7.4 (6.1–8.8) | 652 (511–813) | 9.8 (7.8–12.1) | 32.1 (17.1–49.5) |
| Israel | 46 (37–56) | 1 (0.8–1.2) | 170 (135–212) | 1.6 (1.3–2) | 63.8 (45.4–87.9) |
| Italy | 2002 (1657–2388) | 2.9 (2.4–3.5) | 3073 (2513–3674) | 3.9 (3.2–4.8) | 32.3 (19.2–45.2) |
| Luxembourg | 54 (43–67) | 11.5 (9.3–14.1) | 113 (88–146) | 12.6 (10–16) | 9.9 (−2.6 to 23.6) |
| Malta | 15 (12–19) | 3.8 (3.1–4.6) | 23 (18–30) | 3.4 (2.7–4.2) | −10.4 (−20.6 to 0.9) |
| Monaco | 15 (12–18) | 27.7 (22.6–33.5) | 17 (13–21) | 24.1 (19.3–29.6) | −12.7 (−21.4 to −3.1) |
| Netherlands | 2318 (1954–2754) | 12.7 (10.8–15) | 3713 (2888–4824) | 14.2 (11.3–17.7) | 11.9 (−4.5 to 27.9) |
| Norway | 318 (259–384) | 6.8 (5.5–8.1) | 489 (390–620) | 6.8 (5.4–8.4) | 0 (−10 to 11.2) |
| Portugal | 570 (466–696) | 4.8 (3.9–5.7) | 1159 (913–1479) | 6.7 (5.4–8.5) | 41.5 (23.4–62.8) |
| San Marino | 3 (2–3) | 9.4 (7.7–11.6) | 6 (5–7) | 10.8 (8.7–13.4) | 15.1 (3.7–28.7) |
| Spain | 2408 (1940–2979) | 5.1 (4.1–6.2) | 5105 (4193–6020) | 6.9 (5.8–8.1) | 36.4 (12.5–68.3) |
| Sweden | 1467 (1199–1798) | 14 (11.4–17) | 3473 (2723–4547) | 23.3 (18.6–29.4) | 66.7 (46.7–90.5) |
| Switzerland | 965 (765–1208) | 11 (8.8–13.6) | 1535 (1196–1956) | 11.5 (9.1–14.3) | 4.4 (−8.2 to 18.7) |
| United Kingdom | 3492 (2872–4220) | 5.4 (4.4–6.6) | 5938 (4824–7265) | 7.4 (6–8.9) | 36.4 (26.6–46.4) |
| Western Sub-Saharan Africa | 3338 (2496–4462) | 2.6 (2–3.4) | 9298 (6788–12252) | 2.8 (2.1–3.6) | 6.4 (1.8–11.3) |
| Benin | 68 (50–93) | 2.3 (1.7–3.1) | 250 (178–339) | 2.8 (2.1–3.7) | 20.7 (9.3–35) |
| Burkina Faso | 140 (102–190) | 2.4 (1.8–3.2) | 450 (326–610) | 3 (2.2–4) | 26.1 (12.4–40.8) |
| Cabo Verde | 8 (6–11) | 3.3 (2.5–4.5) | 22 (16–30) | 3.8 (2.9–5.2) | 15.2 (5.8–26) |
| Cameroon | 182 (131–251) | 2.7 (2–3.6) | 697 (502–960) | 3.1 (2.3–4.2) | 14.2 (2.8–26.2) |
| Chad | 85 (63–115) | 2.2 (1.7–3) | 282 (206–385) | 2.8 (2.1–3.7) | 25.1 (13.4–38.3) |
| Côte d'Ivoire | 188 (136–263) | 2.5 (1.9–3.3) | 589 (429–821) | 2.9 (2.2–3.9) | 17.6 (5.3–31.4) |
| Gambia | 15 (11–20) | 2.4 (1.8–3.3) | 46 (34–62) | 2.7 (2–3.7) | 11.8 (0–23.7) |
| Ghana | 251 (185–340) | 2.5 (1.9–3.4) | 695 (497–944) | 2.5 (1.9–3.4) | 0.1 (−13.8 to 15.6) |
| Guinea | 96 (72–131) | 2.3 (1.8–3.1) | 237 (172–318) | 2.7 (2–3.5) | 14.1 (1.4–28.3) |
| Guinea-Bissau | 13 (10–18) | 2.1 (1.6–2.9) | 35 (25–49) | 2.5 (1.9–3.3) | 17.9 (5.6–31.3) |
| Liberia | 43 (32–58) | 2.7 (2–3.6) | 123 (89–169) | 3.1 (2.3–4.1) | 15.1 (2.7–26.7) |
| Mali | 134 (98–183) | 2.4 (1.8–3.2) | 418 (303–561) | 2.8 (2.1–3.7) | 17.7 (5.3–31.4) |
| Mauritania | 34 (25–46) | 2.4 (1.8–3.3) | 90 (66–124) | 2.8 (2.2–3.8) | 16.4 (3.2–30.4) |
| Niger | 115 (84–157) | 2.4 (1.8–3.3) | 385 (274–533) | 2.7 (2–3.6) | 10.4 (−1.2 to 22.1) |
| Nigeria | 1737 (1301–2292) | 2.8 (2.1–3.6) | 4316 (3225–5704) | 2.7 (2.1–3.5) | −1.6 (−6 to 2.9) |
| Sao Tome and Principe | 2 (1–3) | 2.5 (1.9–3.4) | 5 (3–6) | 2.7 (2–3.6) | 8.2 (−5.4 to 21.3) |
| Senegal | 108 (79–145) | 2.3 (1.7–3) | 305 (225–414) | 2.6 (2–3.5) | 14 (1.5–28.1) |
| Sierra Leone | 69 (50–92) | 2.4 (1.8–3.2) | 181 (130–253) | 2.8 (2.1–3.8) | 18.3 (6.3–31.8) |
| Togo | 50 (35–69) | 2.3 (1.7–3.1) | 173 (128–235) | 2.7 (2.1–3.6) | 17.1 (5.3–30) |
