# Supplementary material for: Global, regional, and national burden of cardiomyopathy (including alcoholic cardiomyopathy and others) from 1990 to 2021: An analysis of data from the global burden of disease study 2021 and forecast to 2040
Source: PLoS One. 2026 Jan 30;21(1):e0341687. doi: 10.1371/journal.pone.0341687 (PMC12858021; doi:10.1371/journal.pone.0341687)
Supplement: S11 Table — (DOCX) [file pone.0341687.s022.docx]

**S11 Table. 1990–2021 Global and national mortality trends in alcoholic cardiomyopathy burden.**

| location_name | Number_1990 | ASR per 100,000_1990 | Number_2021 | ASR per 100,000_2021 | Percentage change in the ASRs per 100,000 |
| --- | --- | --- | --- | --- | --- |
| Global | 47073 (44168–49765) | 1.2 (1.1–1.2) | 64011 (56293–69519) | 0.7 (0.7–0.8) | −37.1 (−42.9 to −31.7) |
| Andean Latin America | 2 (1–4) | 0 (0–0) | 3 (1–5) | 0 (0–0) | −56.5 (−76.4 to −29.5) |
| Bolivia (Plurinational State of) | 0 (0–1) | 0 (0–0) | 1 (0–2) | 0 (0–0) | −33.6 (−71.5 to 62.2) |
| Ecuador | 1 (0–1) | 0 (0–0) | 1 (0–1) | 0 (0–0) | −74.7 (−85.5 to −58.2) |
| Peru | 1 (0–2) | 0 (0–0) | 1 (0–3) | 0 (0–0) | −54.1 (−79.4 to −0.6) |
| Australasia | 277 (253–303) | 1.2 (1.1–1.3) | 433 (402–469) | 0.9 (0.8–1) | −27.7 (−35.6 to −18.9) |
| Australia | 210 (193–227) | 1.1 (1–1.2) | 345 (313–380) | 0.8 (0.8–0.9) | −24.9 (−33.1 to −16.5) |
| New Zealand | 67 (55–84) | 1.8 (1.5–2.2) | 88 (80–97) | 1.1 (1–1.2) | −36.6 (−51.2 to −20.1) |
| Caribbean | 155 (108–235) | 0.6 (0.4–0.9) | 998 (800–1208) | 1.9 (1.5–2.3) | 225 (144–312.1) |
| Antigua and Barbuda | 0 (0–0) | 0.1 (0.1–0.1) | 1 (1–1) | 0.9 (0.7–1.1) | 600.2 (453.4–818.4) |
| Bahamas | 2 (2–3) | 1.4 (1.1–1.7) | 16 (13–20) | 3.7 (2.9–4.6) | 172.1 (86.2–288.7) |
| Barbados | 4 (4–5) | 1.6 (1.3–1.9) | 11 (9–15) | 2.3 (1.8–3) | 41.8 (3.1–92.6) |
| Belize | 0 (0–0) | 0.3 (0.2–0.4) | 6 (5–8) | 1.9 (1.6–2.2) | 518.5 (331.8–869.6) |
| Bermuda | 0 (0–0) | 0.3 (0.2–0.4) | 2 (2–3) | 1.7 (1.4–2.1) | 506.9 (269.3–850.4) |
| Cuba | 37 (32–43) | 0.4 (0.3–0.4) | 586 (491–683) | 3.1 (2.6–3.6) | 767.1 (599.1–963.7) |
| Dominica | 0 (0–1) | 0.8 (0.2–1.9) | 1 (0–2) | 1 (0.2–1.9) | 28 (−34.2 to 131.3) |
| Dominican Republic | 13 (5–28) | 0.3 (0.1–0.7) | 70 (13–113) | 0.7 (0.1–1.1) | 121.1 (−19.7 to 313.6) |
| Grenada | 1 (1–1) | 1.7 (1.5–2.1) | 4 (3–5) | 3.5 (2.9–4.1) | 99.1 (52.1–156.7) |
| Guyana | 6 (5–7) | 1.3 (1.1–1.6) | 23 (16–31) | 3.2 (2.3–4.4) | 145.3 (63.4–264.5) |
| Haiti | 61 (27–121) | 1.7 (0.7–3.4) | 146 (42–296) | 1.7 (0.5–3.5) | 3.3 (−40.3 to 76.3) |
| Jamaica | 10 (8–13) | 0.6 (0.5–0.8) | 34 (25–45) | 1.1 (0.8–1.5) | 94.5 (25.2–177.1) |
| Puerto Rico | 8 (7–9) | 0.2 (0.2–0.3) | 27 (22–33) | 0.5 (0.4–0.6) | 119.7 (68.8–178.5) |
| Saint Kitts and Nevis | 0 (0–0) | 0.4 (0.3–0.5) | 2 (1–2) | 2 (1.6–2.7) | 393.8 (259.5–600) |
| Saint Lucia | 1 (1–1) | 1.3 (1.1–1.5) | 12 (9–14) | 4.8 (3.8–5.8) | 273.1 (181.1–405.7) |
| Saint Vincent and the Grenadines | 0 (0–1) | 0.6 (0.6–0.7) | 3 (3–4) | 2.2 (1.9–2.6) | 257.8 (192.1–333.3) |
| Suriname | 1 (0–2) | 0.3 (0.1–0.7) | 4 (1–7) | 0.6 (0.1–1) | 82.4 (−25.6 to 243.1) |
| Trinidad and Tobago | 3 (3–4) | 0.4 (0.3–0.5) | 15 (11–20) | 0.8 (0.6–1) | 109.5 (37.4–230.4) |
| United States Virgin Islands | 1 (0–3) | 1.1 (0.5–2.7) | 2 (0–3) | 1.1 (0.3–1.8) | 4.9 (−53.5 to 98.6) |
| Central Asia | 384 (326–462) | 0.8 (0.6–0.9) | 958 (789–1187) | 1 (0.9–1.3) | 33.9 (5.3–74.7) |
| Armenia | 127 (98–163) | 4.7 (3.6–6) | 68 (51–91) | 1.6 (1.2–2.2) | −64.7 (−75.5 to −47.1) |
| Azerbaijan | 25 (3–69) | 0.4 (0–1.2) | 38 (4–123) | 0.3 (0–1) | −29.1 (−74 to 92.4) |
| Georgia | 25 (19–35) | 0.4 (0.3–0.6) | 19 (14–25) | 0.4 (0.3–0.5) | −11.3 (−42.4 to 31.9) |
| Kazakhstan | 11 (6–19) | 0.1 (0–0.1) | 222 (111–406) | 1.1 (0.6–2) | 1356.8 (699.2–2535.6) |
| Kyrgyzstan | 128 (110–154) | 4 (3.4–4.8) | 461 (381–561) | 8.1 (6.7–9.9) | 101.3 (53.6–162.5) |
| Mongolia | 45 (21–83) | 4 (1.9–7.2) | 121 (43–193) | 4.1 (1.4–6.4) | 2.8 (−52.1 to 100.7) |
| Tajikistan | 0 (0–0) | 0 (0–0) | 0 (0–0) | 0 (0–0) | −39.7 (−77.6 to 30.4) |
| Turkmenistan | 21 (14–32) | 1 (0.6–1.4) | 28 (15–50) | 0.6 (0.3–1) | −39 (−64.9 to 3.8) |
| Uzbekistan | 1 (0–1) | 0 (0–0) | 1 (1–2) | 0 (0–0) | −24.5 (−56.2 to 32.1) |
| Central Europe | 4005 (3622–4386) | 2.9 (2.6–3.1) | 5346 (4288–6113) | 2.7 (2.1–3.1) | −6.6 (−22.2 to 9.3) |
| Albania | 19 (8–30) | 1 (0.4–1.6) | 32 (8–61) | 0.8 (0.2–1.5) | −20.1 (−62.9 to 46.7) |
| Bosnia and Herzegovina | 87 (25–154) | 2.2 (0.6–4) | 114 (22–227) | 1.9 (0.4–3.8) | −11.5 (−58.5 to 64.2) |
| Bulgaria | 24 (21–29) | 0.2 (0.2–0.3) | 41 (32–52) | 0.3 (0.3–0.4) | 45 (5.7–95.1) |
| Croatia | 111 (100–123) | 2.1 (1.9–2.3) | 289 (234–354) | 3.3 (2.6–4.1) | 58.5 (23–97.4) |
| Czechia | 29 (24–35) | 0.2 (0.2–0.3) | 103 (77–132) | 0.6 (0.4–0.7) | 147.9 (70.2–244) |
| Hungary | 1275 (1125–1431) | 9.3 (8.2–10.5) | 1409 (1158–1680) | 8.1 (6.6–9.7) | −13.3 (−32.3 to 9.9) |
| Montenegro | 29 (10–46) | 4.7 (1.5–7.4) | 39 (10–65) | 4.3 (1.1–6.9) | −8.9 (−49.1 to 54) |
| North Macedonia | 55 (18–94) | 3.1 (1–5.2) | 70 (14–144) | 2.3 (0.5–4.5) | −25.9 (−64 to 38.5) |
| Poland | 1104 (1033–1168) | 2.6 (2.4–2.8) | 1796 (1499–2085) | 2.8 (2.3–3.3) | 7.1 (−13.1 to 26.3) |
| Romania | 715 (568–935) | 2.7 (2.2–3.5) | 744 (587–912) | 2.3 (1.8–2.8) | −15.8 (−40.5 to 17.9) |
| Serbia | 345 (125–560) | 3.4 (1.2–6) | 443 (106–715) | 2.9 (0.7–4.5) | −16.1 (−52 to 35.9) |
| Slovakia | 48 (18–82) | 0.8 (0.3–1.4) | 118 (25–183) | 1.4 (0.3–2.1) | 63.4 (−30.9 to 170.1) |
| Slovenia | 101 (67–136) | 4.1 (2.8–5.6) | 72 (57–90) | 1.6 (1.2–2) | −61.7 (−73.9 to −41) |
| Central Latin America | 207 (191–228) | 0.2 (0.2–0.2) | 478 (422–541) | 0.2 (0.2–0.2) | −16.8 (−29.1 to −4.4) |
| Colombia | 11 (10–12) | 0.1 (0–0.1) | 39 (31–48) | 0.1 (0.1–0.1) | 34.9 (5.4–67.5) |
| Costa Rica | 8 (7–9) | 0.4 (0.3–0.5) | 21 (17–24) | 0.4 (0.3–0.4) | −7.5 (−25.9 to 14.8) |
| El Salvador | 2 (0–3) | 0 (0–0.1) | 2 (0–4) | 0 (0–0.1) | −26 (−55.9 to 37.7) |
| Guatemala | 6 (5–7) | 0.1 (0.1–0.2) | 10 (8–12) | 0.1 (0.1–0.1) | −42.7 (−57 to −21.2) |
| Honduras | 6 (1–11) | 0.3 (0.1–0.5) | 15 (2–29) | 0.2 (0–0.4) | −15.1 (−51.6 to 39.7) |
| Mexico | 71 (65–77) | 0.1 (0.1–0.2) | 279 (235–325) | 0.2 (0.2–0.2) | 43.8 (18.5–71.4) |
| Nicaragua | 2 (1–5) | 0.1 (0–0.3) | 5 (1–9) | 0.1 (0–0.2) | −24.1 (−48.3 to 11.9) |
| Panama | 3 (2–3) | 0.2 (0.1–0.2) | 6 (5–8) | 0.1 (0.1–0.2) | −20.8 (−42.3 to 8.2) |
| Venezuela (Bolivarian Republic of) | 100 (88–116) | 0.9 (0.8–1.1) | 101 (72–133) | 0.3 (0.2–0.4) | −64 (−74.8 to −51) |
| Central Sub-Saharan Africa | 1 (0–5) | 0 (0–0) | 2 (0–12) | 0 (0–0) | −25 (−57.5 to 15.2) |
| Angola | 0 (0–1) | 0 (0–0) | 0 (0–2) | 0 (0–0) | −35.5 (−63 to 6.5) |
| Central African Republic | 0 (0–0) | 0 (0–0) | 0 (0–1) | 0 (0–0) | −22.8 (−64.8 to 16.7) |
| Congo | 0 (0–0) | 0 (0–0) | 0 (0–0) | 0 (0–0) | −33.6 (−68.1 to 8.1) |
| Democratic Republic of the Congo | 1 (0–3) | 0 (0–0) | 1 (0–8) | 0 (0–0) | −20 (−60.9 to 30.2) |
| Equatorial Guinea | 0 (0–0) | 0 (0–0) | 0 (0–0) | 0 (0–0) | −57.7 (−80.5 to −12.6) |
| Gabon | 0 (0–0) | 0 (0–0) | 0 (0–0) | 0 (0–0) | −42.7 (−67.6 to −5) |
| East Asia | 573 (253–1216) | 0.1 (0–0.1) | 2000 (426–3150) | 0.1 (0–0.2) | 56.2 (−45 to 181.5) |
| China | 477 (170–1105) | 0.1 (0–0.1) | 1861 (318–2987) | 0.1 (0–0.2) | 80.3 (−43.2 to 261.7) |
| Democratic People's Republic of Korea | 24 (9–49) | 0.1 (0.1–0.3) | 52 (16–113) | 0.2 (0.1–0.4) | 17.1 (−37.6 to 95.8) |
| Taiwan (Province of China) | 72 (67–78) | 0.5 (0.4–0.5) | 86 (76–97) | 0.2 (0.2–0.3) | −48 (−54.7 to −40.3) |
| Eastern Europe | 24150 (22429–25915) | 9.3 (8.6–9.9) | 38835 (34484–42737) | 13.1 (11.6–14.4) | 40.9 (26.7–55.9) |
| Belarus | 939 (764–1151) | 7.7 (6.3–9.4) | 800 (632–994) | 6.2 (4.9–7.7) | −20 (−43.2 to 11.6) |
| Estonia | 119 (92–145) | 6.3 (4.9–7.7) | 84 (71–98) | 3.9 (3.3–4.6) | −38.1 (−50.8 to −21.9) |
| Latvia | 249 (213–293) | 7.7 (6.6–9) | 397 (330–468) | 14.5 (12.2–17) | 89.5 (58.4–127.1) |
| Lithuania | 144 (112–174) | 3.4 (2.7–4.1) | 168 (140–199) | 3.9 (3.3–4.6) | 14.8 (−8.4 to 47.2) |
| Republic of Moldova | 67 (58–78) | 1.5 (1.3–1.7) | 218 (178–259) | 3.9 (3.2–4.7) | 165.1 (121.9–216.7) |
| Russian Federation | 14016 (13692–14303) | 8.2 (8–8.4) | 28546 (25511–31473) | 13.9 (12.4–15.2) | 68.5 (50.8–84.9) |
| Ukraine | 8615 (7166–10078) | 13.6 (11.3–15.9) | 8623 (6123–11465) | 13.6 (9.6–18.1) | 0.1 (−32 to 42.1) |
| Eastern Sub-Saharan Africa | 2 (0–9) | 0 (0–0) | 3 (0–23) | 0 (0–0) | −28.2 (−61.9 to 3.8) |
| Burundi | 0 (0–0) | 0 (0–0) | 0 (0–1) | 0 (0–0) | −33.7 (−79.3 to 15.8) |
| Comoros | 0 (0–0) | 0 (0–0) | 0 (0–0) | 0 (0–0) | −46.2 (−75.7 to −4.4) |
| Djibouti | 0 (0–0) | 0 (0–0) | 0 (0–0) | 0 (0–0) | −29.5 (−66 to 30.3) |
| Eritrea | 0 (0–0) | 0 (0–0) | 0 (0–0) | 0 (0–0) | −42.6 (−72.2 to −5.7) |
| Ethiopia | 0 (0–2) | 0 (0–0) | 1 (0–4) | 0 (0–0) | −43.9 (−78.5 to 5.7) |
| Kenya | 0 (0–1) | 0 (0–0) | 0 (0–2) | 0 (0–0) | −16 (−47.1 to 13.8) |
| Madagascar | 0 (0–1) | 0 (0–0) | 0 (0–3) | 0 (0–0) | −24.7 (−69.1 to 25.2) |
| Malawi | 0 (0–0) | 0 (0–0) | 0 (0–1) | 0 (0–0) | −5.4 (−58.7 to 54.8) |
| Mozambique | 0 (0–1) | 0 (0–0) | 0 (0–1) | 0 (0–0) | −3.5 (−56.3 to 87.8) |
| Rwanda | 0 (0–0) | 0 (0–0) | 0 (0–1) | 0 (0–0) | −50.9 (−80.1 to −9.4) |
| Somalia | 0 (0–0) | 0 (0–0) | 0 (0–1) | 0 (0–0) | −25.1 (−72.2 to 37.2) |
| South Sudan | 0 (0–0) | 0 (0–0) | 0 (0–0) | 0 (0–0) | −32.6 (−69.4 to 19) |
| Uganda | 0 (0–1) | 0 (0–0) | 0 (0–1) | 0 (0–0) | −49.3 (−78.1 to −9.8) |
| United Republic of Tanzania | 0 (0–1) | 0 (0–0) | 0 (0–3) | 0 (0–0) | −28.2 (−72.5 to 24.3) |
| Zambia | 0 (0–0) | 0 (0–0) | 0 (0–2) | 0 (0–0) | 89.6 (−57 to 281.1) |
| High-income Asia Pacific | 673 (625–735) | 0.3 (0.3–0.4) | 489 (443–532) | 0.1 (0.1–0.1) | −60.4 (−64.2 to −55.9) |
| Brunei Darussalam | 1 (0–2) | 1 (0.3–1.6) | 2 (1–4) | 0.5 (0.2–1) | −46.1 (−64.5 to −19.7) |
| Japan | 656 (608–717) | 0.4 (0.4–0.4) | 474 (434–512) | 0.2 (0.2–0.2) | −54.6 (−59.1 to −49.8) |
| Republic of Korea | 10 (2–17) | 0 (0–0) | 11 (1–28) | 0 (0–0) | −54.6 (−77.4 to −19.2) |
| Singapore | 5 (5–6) | 0.2 (0.2–0.2) | 2 (2–3) | 0 (0–0) | −86.3 (−88.5 to −83.8) |
| High-income North America | 4778 (4404–5117) | 1.4 (1.3–1.5) | 6015 (5590–6391) | 1 (0.9–1.1) | −28 (−35.4 to −20.3) |
| Canada | 251 (215–292) | 0.8 (0.7–0.9) | 370 (333–410) | 0.6 (0.5–0.6) | −27 (−40.5 to −11.2) |
| Greenland | 0 (0–1) | 0.9 (0.2–1.9) | 1 (0–1) | 0.9 (0.1–1.6) | −2.5 (−52.5 to 58) |
| United States of America | 4527 (4178–4836) | 1.5 (1.4–1.6) | 5644 (5235–6018) | 1.1 (1–1.1) | −27.7 (−35.3 to −19.9) |
| North Africa and Middle East | 102 (24–201) | 0.1 (0–0.1) | 179 (51–351) | 0 (0–0.1) | −35.1 (−53.1 to −6.2) |
| Afghanistan | 9 (2–20) | 0.1 (0–0.3) | 10 (3–23) | 0.1 (0–0.3) | −28 (−59.6 to 38.3) |
| Algeria | 11 (2–22) | 0.1 (0–0.2) | 21 (5–45) | 0.1 (0–0.2) | −30.6 (−60.2 to 35) |
| Bahrain | 0 (0–1) | 0.3 (0.1–0.4) | 1 (0–3) | 0.2 (0–0.3) | −45.7 (−68 to −2.7) |
| Egypt | 1 (0–1) | 0 (0–0) | 1 (0–2) | 0 (0–0) | −53.6 (−75.8 to −12.3) |
| Iran (Islamic Republic of) | 20 (4–43) | 0.1 (0–0.2) | 36 (7–78) | 0 (0–0.1) | −42.6 (−64 to −15.5) |
| Iraq | 1 (0–2) | 0 (0–0) | 2 (0–3) | 0 (0–0) | −23.1 (−64.6 to 82.2) |
| Jordan | 0 (0–0) | 0 (0–0) | 0 (0–0) | 0 (0–0) | −56.1 (−75.4 to −11.4) |
| Kuwait | 2 (2–3) | 0.3 (0.2–0.4) | 4 (3–6) | 0.1 (0.1–0.2) | −53.6 (−67.7 to −36.1) |
| Lebanon | 0 (0–0) | 0 (0–0) | 0 (0–0) | 0 (0–0) | −53.8 (−76.7 to 20.4) |
| Libya | 1 (0–2) | 0.1 (0–0.1) | 3 (1–5) | 0 (0–0.1) | −22 (−57.5 to 80) |
| Morocco | 14 (3–30) | 0.1 (0–0.2) | 26 (7–63) | 0.1 (0–0.2) | −16.5 (−49.9 to 50.8) |
| Oman | 0 (0–0) | 0 (0–0) | 0 (0–1) | 0 (0–0) | −54.7 (−80.3 to 18.6) |
| Palestine | 1 (0–1) | 0.1 (0–0.1) | 1 (0–2) | 0 (0–0.1) | −34.2 (−65.8 to 18.5) |
| Qatar | 0 (0–0) | 0.1 (0–0.2) | 0 (0–2) | 0 (0–0.1) | −44.8 (−76.3 to 17.2) |
| Saudi Arabia | 7 (1–12) | 0.1 (0–0.2) | 14 (3–25) | 0.1 (0–0.1) | −45 (−74.2 to 14.1) |
| Sudan | 10 (2–22) | 0.1 (0–0.3) | 13 (4–29) | 0.1 (0–0.2) | −40.5 (−65.5 to 14.9) |
| Syrian Arab Republic | 6 (1–11) | 0.1 (0–0.2) | 10 (3–19) | 0.1 (0–0.2) | −31.9 (−67.8 to 35.7) |
| Tunisia | 4 (1–8) | 0.1 (0–0.2) | 9 (2–19) | 0.1 (0–0.2) | −17.9 (−51.6 to 41.2) |
| Turkey | 7 (1–17) | 0 (0–0) | 11 (2–24) | 0 (0–0) | −39.3 (−71 to 23) |
| United Arab Emirates | 1 (0–2) | 0.1 (0–0.3) | 3 (1–9) | 0.1 (0–0.2) | −50.8 (−75.2 to −7.3) |
| Yemen | 7 (1–16) | 0.1 (0–0.3) | 13 (3–29) | 0.1 (0–0.2) | −35.6 (−63.6 to 17.2) |
| Oceania | 3 (0–5) | 0.1 (0–0.1) | 5 (1–11) | 0.1 (0–0.1) | −28.5 (−55 to 14) |
| American Samoa | 0 (0–0) | 0.3 (0.1–0.5) | 0 (0–0) | 0.1 (0–0.3) | −55.9 (−73.9 to −8.9) |
| Cook Islands | 0 (0–0) | 0 (0–0) | 0 (0–0) | 0 (0–0) | −66.6 (−85.9 to −28.5) |
| Fiji | 0 (0–0) | 0 (0–0) | 0 (0–0) | 0 (0–0) | −20.2 (−49 to 23.4) |
| Guam | 0 (0–0) | 0.2 (0.1–0.4) | 0 (0–0) | 0.1 (0–0.2) | −44.4 (−64 to −20) |
| Kiribati | 0 (0–0) | 0.2 (0–0.3) | 0 (0–0) | 0.2 (0–0.3) | −7.8 (−44.8 to 57.6) |
| Marshall Islands | 0 (0–0) | 0.1 (0–0.2) | 0 (0–0) | 0.1 (0–0.1) | −31.5 (−58.4 to 20.4) |
| Micronesia (Federated States of) | 0 (0–0) | 0.1 (0–0.2) | 0 (0–0) | 0.1 (0–0.1) | −36.3 (−63.3 to 17.2) |
| Nauru | 0 (0–0) | 0.1 (0–0.2) | 0 (0–0) | 0.1 (0–0.2) | −16.4 (−51.4 to 56) |
| Niue | 0 (0–0) | 0.1 (0–0.1) | 0 (0–0) | 0 (0–0.1) | −32.1 (−60.4 to 14.4) |
| Northern Mariana Islands | 0 (0–0) | 0.2 (0.1–0.4) | 0 (0–0) | 0.1 (0–0.4) | −42.9 (−69.7 to 35.7) |
| Palau | 0 (0–0) | 0.1 (0–0.1) | 0 (0–0) | 0 (0–0.1) | −30.7 (−61 to 19.1) |
| Papua New Guinea | 2 (0–4) | 0.1 (0–0.2) | 4 (1–8) | 0.1 (0–0.1) | −27.3 (−60.9 to 31.1) |
| Samoa | 0 (0–0) | 0.1 (0–0.2) | 0 (0–0) | 0.1 (0–0.1) | −34.1 (−62.2 to 5.5) |
| Solomon Islands | 0 (0–0) | 0.1 (0–0.2) | 0 (0–0) | 0.1 (0–0.1) | −19.8 (−52 to 45.5) |
| Tokelau | 0 (0–0) | 0.1 (0–0.2) | 0 (0–0) | 0.1 (0–0.1) | −31.8 (−61.1 to 25.8) |
| Tonga | 0 (0–0) | 0.1 (0–0.1) | 0 (0–0) | 0 (0–0.1) | −31.4 (−58.4 to 23.8) |
| Tuvalu | 0 (0–0) | 0.1 (0–0.2) | 0 (0–0) | 0.1 (0–0.1) | −38.4 (−63.6 to 6.7) |
| Vanuatu | 0 (0–0) | 0.1 (0–0.2) | 0 (0–0) | 0.1 (0–0.1) | −29.6 (−57.8 to 13.2) |
| South Asia | 713 (152–1737) | 0.1 (0–0.3) | 1451 (269–3395) | 0.1 (0–0.2) | −15.8 (−45.9 to 23.7) |
| Bangladesh | 78 (15–199) | 0.2 (0–0.4) | 153 (23–442) | 0.1 (0–0.3) | −30 (−62.5 to 34.4) |
| Bhutan | 0 (0–1) | 0.1 (0–0.3) | 1 (0–2) | 0.1 (0–0.3) | −17.6 (−55.8 to 65.9) |
| India | 559 (121–1301) | 0.1 (0–0.2) | 1139 (211–2639) | 0.1 (0–0.2) | −14.2 (−46.1 to 26.7) |
| Nepal | 13 (3–32) | 0.1 (0–0.3) | 23 (5–51) | 0.1 (0–0.2) | −22.7 (−55.6 to 67.4) |
| Pakistan | 62 (14–188) | 0.1 (0–0.3) | 136 (26–367) | 0.1 (0–0.3) | −5.3 (−40.9 to 57) |
| Southeast Asia | 170 (40–336) | 0.1 (0–0.1) | 365 (67–605) | 0.1 (0–0.1) | −10.1 (−42.8 to 30.6) |
| Cambodia | 2 (1–5) | 0 (0–0.1) | 6 (1–12) | 0 (0–0.1) | −3.4 (−46.8 to 66.1) |
| Indonesia | 56 (12–107) | 0 (0–0.1) | 137 (22–257) | 0.1 (0–0.1) | 13.5 (−29.4 to 73.3) |
| Lao People's Democratic Republic | 2 (0–3) | 0.1 (0–0.1) | 3 (1–6) | 0.1 (0–0.1) | −20.8 (−58.8 to 35.7) |
| Malaysia | 0 (0–0) | 0 (0–0) | 0 (0–1) | 0 (0–0) | −53.8 (−75.5 to 38.6) |
| Maldives | 0 (0–0) | 0.1 (0–0.2) | 0 (0–0) | 0 (0–0.1) | −49.4 (−79.4 to −0.6) |
| Mauritius | 2 (2–3) | 0.3 (0.3–0.3) | 4 (3–5) | 0.2 (0.2–0.3) | −27.1 (−39.3 to −13) |
| Myanmar | 19 (5–35) | 0.1 (0–0.1) | 31 (5–57) | 0.1 (0–0.1) | −17.7 (−55.4 to 50.5) |
| Philippines | 32 (7–62) | 0.1 (0–0.2) | 72 (15–149) | 0.1 (0–0.2) | −17.2 (−43.6 to 10.3) |
| Seychelles | 0 (0–0) | 0.1 (0–0.1) | 0 (0–0) | 0 (0–0.1) | −18.4 (−53.5 to 26.2) |
| Sri Lanka | 27 (5–65) | 0.2 (0–0.6) | 21 (4–46) | 0.1 (0–0.2) | −65 (−84.2 to −28) |
| Thailand | 6 (2–15) | 0 (0–0) | 27 (2–49) | 0 (0–0.1) | 77.2 (−59.2 to 251.8) |
| Timor-Leste | 0 (0–1) | 0.1 (0–0.1) | 1 (0–1) | 0.1 (0–0.1) | 3.4 (−46 to 79.4) |
| Viet Nam | 23 (4–43) | 0.1 (0–0.1) | 64 (11–122) | 0.1 (0–0.1) | 13.1 (−41.6 to 131.3) |
| Southern Latin America | 582 (504–668) | 1.2 (1.1–1.4) | 175 (156–195) | 0.2 (0.2–0.2) | −83.5 (−86.1 to −80.4) |
| Uruguay | 117 (101–131) | 3.1 (2.7–3.4) | 52 (46–59) | 1 (0.9–1.2) | −66.6 (−72.3 to −58.5) |
| Argentina | 418 (351–503) | 1.3 (1.1–1.5) | 84 (71–98) | 0.2 (0.1–0.2) | −88.1 (−90.6 to −85.3) |
| Chile | 47 (41–52) | 0.4 (0.4–0.5) | 39 (33–45) | 0.2 (0.1–0.2) | −65 (−71.3 to −57.3) |
| Southern Sub-Saharan Africa | 3 (0–6) | 0 (0–0) | 3 (1–9) | 0 (0–0) | −27.2 (−55.8 to 15.6) |
| Botswana | 0 (0–0) | 0 (0–0) | 0 (0–0) | 0 (0–0) | −57.6 (−80.4 to −18.9) |
| Eswatini | 0 (0–0) | 0 (0–0) | 0 (0–0) | 0 (0–0) | −64.3 (−80.3 to −35.4) |
| Lesotho | 0 (0–0) | 0 (0–0) | 0 (0–0) | 0 (0–0) | −55.8 (−79.8 to −1.2) |
| Namibia | 0 (0–0) | 0 (0–0) | 0 (0–0) | 0 (0–0) | −46.5 (−72 to 4.2) |
| South Africa | 2 (0–2) | 0 (0–0) | 1 (0–2) | 0 (0–0) | −32.5 (−62.7 to 15.7) |
| Zimbabwe | 1 (0–4) | 0 (0–0.1) | 2 (0–7) | 0 (0–0.1) | −14.9 (−52.5 to 71.7) |
| Tropical Latin America | 1740 (1609–1925) | 1.6 (1.5–1.8) | 1186 (1103–1270) | 0.4 (0.4–0.5) | −72.2 (−75.1 to −69) |
| Brazil | 1735 (1604–1919) | 1.7 (1.5–1.8) | 1178 (1092–1263) | 0.5 (0.4–0.5) | −72.3 (−75.2 to −69.1) |
| Paraguay | 5 (1–9) | 0.2 (0–0.4) | 8 (2–17) | 0.1 (0–0.3) | −41.4 (−65.9 to 20.8) |
| Western Europe | 8401 (7572–9156) | 1.5 (1.4–1.7) | 4939 (4446–5379) | 0.6 (0.5–0.6) | −61.5 (−66.9 to −56.3) |
| Andorra | 1 (0–1) | 1.4 (0.5–2.4) | 1 (0–2) | 0.7 (0.2–1.1) | −50.4 (−71.8 to −11.4) |
| Austria | 722 (643–792) | 6.1 (5.5–6.7) | 151 (133–169) | 0.9 (0.8–1) | −85.3 (−87.1 to −83.3) |
| Belgium | 87 (79–96) | 0.6 (0.5–0.7) | 104 (92–117) | 0.5 (0.4–0.5) | −19.2 (−29.1 to −7.6) |
| Cyprus | 5 (1–9) | 0.6 (0.1–1.1) | 5 (1–10) | 0.3 (0.1–0.5) | −58.3 (−75.8 to −27.9) |
| Denmark | 55 (49–62) | 0.7 (0.7–0.8) | 47 (40–54) | 0.4 (0.4–0.5) | −43.9 (−54.1 to −31.5) |
| Finland | 243 (211–276) | 3.7 (3.2–4.2) | 231 (207–258) | 2.3 (2–2.5) | −39.2 (−50 to −26.4) |
| France | 377 (348–408) | 0.5 (0.4–0.5) | 456 (389–528) | 0.4 (0.3–0.4) | −23 (−34.8 to −10.7) |
| Germany | 5411 (4618–6125) | 4.5 (3.9–5.1) | 2609 (2298–2895) | 1.5 (1.3–1.6) | −67 (−72.8 to −60.4) |
| Greece | 20 (16–24) | 0.1 (0.1–0.2) | 23 (19–27) | 0.1 (0.1–0.1) | −21.7 (−39.1 to 4.6) |
| Iceland | 1 (1–1) | 0.4 (0.4–0.4) | 1 (1–1) | 0.2 (0.2–0.2) | −52.7 (−60.9 to −44.1) |
| Ireland | 50 (46–54) | 1.3 (1.2–1.4) | 36 (29–42) | 0.5 (0.4–0.5) | −64.2 (−70.6 to −56.8) |
| Israel | 4 (4–5) | 0.1 (0.1–0.1) | 6 (5–7) | 0.1 (0–0.1) | −45.5 (−55.4 to −33.1) |
| Italy | 135 (124–143) | 0.2 (0.2–0.2) | 119 (97–138) | 0.1 (0.1–0.1) | −41.9 (−52.9 to −31.2) |
| Luxembourg | 7 (6–7) | 1.3 (1.2–1.4) | 6 (5–6) | 0.5 (0.4–0.6) | −59.3 (−65.9 to −51.8) |
| Malta | 2 (2–2) | 0.5 (0.5–0.6) | 2 (1–2) | 0.2 (0.2–0.2) | −64.7 (−70.9 to −56.9) |
| Monaco | 2 (1–3) | 3.3 (1.2–5.1) | 2 (1–2) | 1.7 (0.7–2.6) | −49.3 (−69.7 to −19.4) |
| Netherlands | 335 (306–365) | 1.7 (1.6–1.9) | 160 (138–182) | 0.5 (0.4–0.5) | −72.2 (−76 to −68) |
| Norway | 24 (23–25) | 0.4 (0.4–0.4) | 16 (14–18) | 0.2 (0.2–0.2) | −56 (−60.8 to −50.4) |
| Portugal | 54 (50–59) | 0.4 (0.4–0.5) | 67 (56–77) | 0.3 (0.3–0.4) | −26 (−38.8 to −12.2) |
| San Marino | 0 (0–0) | 0.6 (0.2–1.3) | 0 (0–0) | 0.3 (0.1–0.6) | −49 (−72.5 to −12.6) |
| Spain | 319 (285–353) | 0.6 (0.6–0.7) | 246 (207–289) | 0.3 (0.2–0.3) | −54.9 (−62.6 to −46.1) |
| Sweden | 36 (32–39) | 0.3 (0.3–0.3) | 91 (75–109) | 0.5 (0.4–0.6) | 76.8 (42.7 to 117.6) |
| Switzerland | 136 (119–157) | 1.4 (1.2–1.6) | 93 (76–111) | 0.5 (0.4–0.6) | −61.3 (−71.5 to −50.6) |
| United Kingdom | 367 (353–376) | 0.5 (0.5–0.5) | 466 (434–494) | 0.4 (0.4–0.5) | −7 (−13 to −0.8) |
| Western Sub-Saharan Africa | 153 (21–282) | 0.2 (0–0.3) | 148 (23–382) | 0.1 (0–0.2) | −60.8 (−73.4 to −37.6) |
| Benin | 2 (0–5) | 0.1 (0–0.3) | 3 (0–7) | 0.1 (0–0.1) | −51.9 (−78.1 to 8.4) |
| Burkina Faso | 8 (1–20) | 0.2 (0–0.4) | 8 (1–20) | 0.1 (0–0.2) | −53.5 (−79.9 to 7.3) |
| Cabo Verde | 0 (0–0) | 0 (0–0) | 0 (0–0) | 0 (0–0) | −26.1 (−72.5 to 119.6) |
| Cameroon | 6 (1–15) | 0.1 (0–0.3) | 9 (1–22) | 0.1 (0–0.2) | −51.9 (−78.8 to 8.2) |
| Chad | 4 (0–9) | 0.1 (0–0.3) | 4 (1–12) | 0.1 (0–0.2) | −46.8 (−75.3 to 12.4) |
| Côte d'Ivoire | 8 (1–16) | 0.2 (0–0.3) | 9 (1–20) | 0.1 (0–0.2) | −57.5 (−80.1 to −9.6) |
| Gambia | 1 (0–1) | 0.2 (0–0.4) | 1 (0–2) | 0.1 (0–0.2) | −55.2 (−80.7 to 8.3) |
| Ghana | 31 (5–70) | 0.4 (0.1–0.9) | 46 (7–113) | 0.2 (0–0.5) | −45.9 (−74.9 to 7.1) |
| Guinea | 4 (1–8) | 0.1 (0–0.3) | 4 (0–9) | 0.1 (0–0.2) | −44.2 (−73.3 to 17.6) |
| Guinea-Bissau | 1 (0–2) | 0.2 (0–0.4) | 1 (0–1) | 0.1 (0–0.2) | −57.1 (−82.4 to 6.6) |
| Liberia | 1 (0–4) | 0.1 (0–0.3) | 2 (0–4) | 0.1 (0–0.2) | −53 (−81.1 to 8.6) |
| Mali | 4 (0–9) | 0.1 (0–0.2) | 4 (0–11) | 0 (0–0.1) | −59 (−82 to −11.6) |
| Mauritania | 1 (0–3) | 0.1 (0–0.3) | 1 (0–3) | 0 (0–0.1) | −60.6 (−84.3 to −7) |
| Niger | 4 (0–10) | 0.2 (0–0.4) | 5 (1–13) | 0.1 (0–0.2) | −59.4 (−82.4 to −12.6) |
| Nigeria | 70 (8–142) | 0.2 (0–0.3) | 43 (6–120) | 0 (0–0.1) | −73.6 (−83.8 to −48.7) |
| Sao Tome and Principe | 0 (0–0) | 0.1 (0–0.3) | 0 (0–0) | 0.1 (0–0.1) | −55.1 (−81.1 to 15.2) |
| Senegal | 5 (1–11) | 0.1 (0–0.3) | 5 (1–13) | 0.1 (0–0.2) | −58.8 (−82.9 to −0.6) |
| Sierra Leone | 3 (0–8) | 0.2 (0–0.4) | 3 (0–7) | 0.1 (0–0.2) | −58.7 (−82.6 to −6.2) |
| Togo | 2 (0–4) | 0.1 (0–0.3) | 3 (0–7) | 0.1 (0–0.2) | −47.9 (−76.4 to 17.4) |
