# Supplementary material for: Global, regional, and national burden of cardiomyopathy (including alcoholic cardiomyopathy and others) from 1990 to 2021: An analysis of data from the global burden of disease study 2021 and forecast to 2040
Source: PLoS One. 2026 Jan 30;21(1):e0341687. doi: 10.1371/journal.pone.0341687 (PMC12858021; doi:10.1371/journal.pone.0341687)
Supplement: S12 Table — (DOCX) [file pone.0341687.s023.docx]

**S12 Table.** **1990–2021 Global and national DALYs trends in alcoholic cardiomyopathy burden.**

| location_name | Number_1990 | ASR per 100,000_1990 | Number_2021 | ASR per 100,000_2021 | Percentage change in the ASRs per 100,000 |
| --- | --- | --- | --- | --- | --- |
| Global | 1561763 (1456365–1653053) | 35.9 (33.5–38) | 2185528 (1928589–2368736) | 25.3 (22.4–27.5) | −29.3 (−35.3 to −23.7) |
| Andean Latin America | 36 (15–61) | 0.2 (0.1–0.3) | 50 (16–80) | 0.1 (0–0.1) | −55.4 (−74.6 to −30.1) |
| Bolivia (Plurinational State of) | 8 (1–18) | 0.3 (0–0.6) | 16 (2–32) | 0.2 (0–0.4) | −33.1 (−70.5 to 59.4) |
| Ecuador | 12 (9–18) | 0.2 (0.2–0.4) | 9 (6–13) | 0.1 (0–0.1) | −76.6 (−86.2 to −62.3) |
| Peru | 16 (3–30) | 0.2 (0–0.3) | 24 (6–44) | 0.1 (0–0.1) | −50.3 (−75.3 to 2.4) |
| Australasia | 7967 (7348–8654) | 35.5 (32.8–38.5) | 12459 (11570–13526) | 28.2 (26.2–30.6) | −20.7 (−28.8 to −10.7) |
| Australia | 5946 (5522–6345) | 31.8 (29.5–33.8) | 10014 (9165–10979) | 27.1 (24.8–29.7) | −14.6 (−23.8 to −4.8) |
| New Zealand | 2021 (1672–2513) | 54.7 (45.2–68.2) | 2445 (2220–2687) | 33.6 (30.4–36.7) | −38.6 (−51.6 to −23.1) |
| Caribbean | 5456 (3825–8086) | 19.3 (13.5–28.7) | 30773 (24178–37785) | 57.8 (45.2–71.3) | 200.1 (130.1–276.9) |
| Antigua and Barbuda | 2 (2–2) | 4 (3.2–4.7) | 29 (24–34) | 25.4 (21.7–30.1) | 540.7 (411.4–728.3) |
| Bahamas | 90 (72–109) | 46.2 (36.8–56.8) | 562 (446–711) | 123.8 (98.4–156.6) | 167.8 (84.3–275.7) |
| Barbados | 135 (111–156) | 54 (44.4–62.4) | 328 (253–419) | 72.5 (56.7–92.4) | 34.1 (−2.5 to 83.5) |
| Belize | 11 (7–15) | 10.2 (6.4–14) | 229 (192–272) | 62.2 (52.5–73.9) | 507.6 (333.7–825.9) |
| Bermuda | 6 (4–9) | 9.3 (6.7–13.1) | 59 (49–73) | 53 (43.6–65.2) | 469.4 (257.1–776.5) |
| Cuba | 1319 (1139–1499) | 12.5 (10.8–14.3) | 16814 (14088–19578) | 95.8 (80.9–111.4) | 665.5 (525.4–838.8) |
| Dominica | 14 (4–32) | 24.7 (7.9–55.4) | 27 (6–51) | 31.9 (6.6–59.8) | 29.3 (−30.4 to 135.1) |
| Dominican Republic | 487 (197–1014) | 10.2 (4.1–21.3) | 2486 (537–4056) | 23 (4.9–37.4) | 124.6 (−12.1 to 296.1) |
| Grenada | 36 (31–42) | 56.2 (47.7–65.7) | 132 (109–156) | 107.1 (89–126.9) | 90.5 (47.1–148.3) |
| Guyana | 199 (168–239) | 42.3 (35.2–50.6) | 772 (556–1037) | 104.1 (75.1–140.2) | 146.1 (65.1–262.9) |
| Haiti | 2136 (949–4096) | 53.2 (23.7–103.1) | 5264 (1556–10710) | 55.1 (16.4–110.1) | 3.5 (−41.8 to 76) |
| Jamaica | 339 (254–469) | 19.3 (14.7–26.4) | 1065 (764–1414) | 34.6 (24.9–46) | 79.2 (12.6–165.1) |
| Puerto Rico | 259 (228–299) | 7.2 (6.3–8.3) | 805 (662–966) | 16.3 (13.4–19.6) | 126.5 (76.8–182.1) |
| Saint Kitts and Nevis | 5 (4–6) | 13.5 (10.6–17.4) | 48 (37–63) | 60.3 (47.2–79.1) | 345 (217.9–523.8) |
| Saint Lucia | 38 (31–44) | 41.5 (34–48.5) | 353 (280–427) | 148.1 (117.7–178.7) | 257 (170.7–380.2) |
| Saint Vincent and the Grenadines | 16 (14–18) | 20.6 (18.7–23.1) | 97 (82–114) | 69.3 (58.4–81) | 236 (172.5–306.3) |
| Suriname | 32 (12–69) | 10.7 (3.8–23.5) | 131 (27–219) | 19.7 (4.1–33.1) | 84.7 (−17.4 to 229.6) |
| Trinidad and Tobago | 112 (85–137) | 12.1 (9.3–15) | 480 (342–630) | 26 (18.6–34.2) | 114.6 (40.8–235.7) |
| United States Virgin Islands | 36 (17–89) | 35.6 (16.8–89.2) | 50 (15–79) | 40.3 (12.3–67.7) | 13.2 (−45.7 to 108.3) |
| Central Asia | 13858 (11872–16559) | 26 (22.2–31.2) | 35980 (29711–44305) | 36.7 (30.4–45.2) | 41.1 (11.8–81.7) |
| Armenia | 4037 (3094–5102) | 133.8 (103–169.7) | 1981 (1501–2641) | 50.5 (38.4–67.1) | −62.3 (−73.6 to −43.1) |
| Azerbaijan | 969 (125–2689) | 16.2 (2.1–45) | 1370 (153–4466) | 11 (1.3–35.2) | −32.2 (−76.2 to 78.8) |
| Georgia | 859 (645–1189) | 14.1 (10.7–19.6) | 622 (467–816) | 12.8 (9.7–16.7) | −8.9 (−40.3 to 35.6) |
| Kazakhstan | 445 (231–789) | 3 (1.6–5.3) | 8295 (4148–15137) | 40.3 (20.2–73.2) | 1233.5 (628.5–2336.9) |
| Kyrgyzstan | 4995 (4301–5932) | 150.4 (128.6–180.7) | 17853 (14872–21504) | 289.5 (240–349) | 92.5 (49.2–147.7) |
| Mongolia | 1686 (800–3075) | 130.1 (61.3–238.2) | 4722 (1714–7354) | 144.8 (52.1–227.8) | 11.4 (−48 to 119) |
| Tajikistan | 1 (0–3) | 0 (0–0.1) | 2 (0–5) | 0 (0–0.1) | −37.2 (−76.8 to 37.7) |
| Turkmenistan | 839 (542–1233) | 34.9 (22.5–51.7) | 1088 (573–1938) | 21.5 (11.4–38.2) | −38.2 (−65.3 to 5.7) |
| Uzbekistan | 26 (17–38) | 0.2 (0.1–0.3) | 46 (33–63) | 0.1 (0.1–0.2) | −27 (−56.1 to 27.1) |
| Central Europe | 111046 (99953–122127) | 77.1 (69.4–84.9) | 149199 (119378–170873) | 81.7 (65.1–93.8) | 5.9 (−11.7 to 23.4) |
| Albania | 504 (227–829) | 22.7 (10.3–36.9) | 814 (210–1547) | 21 (5.6–39.3) | −7.4 (−56.7 to 72.6) |
| Bosnia and Herzegovina | 2599 (864–4570) | 58.6 (18.6–102.7) | 2977 (615–5897) | 55.2 (11.7–109.8) | −5.8 (−53.9 to 69.6) |
| Bulgaria | 677 (581–784) | 6 (5.2–7) | 1090 (822–1383) | 9.8 (7.4–12.4) | 61.7 (15.6–121.3) |
| Croatia | 2560 (2326–2800) | 44.9 (40.8–49.1) | 6558 (5168–8151) | 84.1 (65.7–104.6) | 87.6 (43.9–135.2) |
| Czechia | 854 (725–1003) | 6.7 (5.7–7.8) | 2980 (2233–3804) | 18 (13.4–22.9) | 168.2 (88.3–265.3) |
| Hungary | 34222 (30496–38125) | 254.8 (227.7–283.9) | 38744 (31727–46617) | 245.9 (201–297.7) | −3.5 (−25.4 to 22.7) |
| Montenegro | 859 (326–1373) | 133.3 (50–212) | 1098 (285–1847) | 124.2 (32.3–207.5) | −6.8 (−47.6 to 54.6) |
| North Macedonia | 1518 (577–2614) | 78.2 (29.5–134.3) | 1972 (426–4231) | 62.3 (13.6–131.7) | −20.3 (−63.1 to 51.1) |
| Poland | 27684 (26078–29099) | 64.8 (61–68.1) | 52916 (43711–61729) | 89.8 (73.6–105.1) | 38.5 (12.5–63.1) |
| Romania | 23965 (19218–31042) | 89.4 (72.1–115.5) | 21359 (16843–26466) | 72.4 (57.3–90.2) | −19 (−41.9 to 10.8) |
| Serbia | 9741 (3862–14494) | 87.8 (34.4–138.6) | 11398 (3017–17571) | 81.5 (22.2–124.3) | −7.2 (−44.9 to 41.2) |
| Slovakia | 1517 (594–2488) | 26.5 (10.1–43.7) | 3597 (753–5500) | 43.8 (9–66.8) | 65.2 (−27.9 to 167.7) |
| Slovenia | 2569 (1779–3327) | 106.4 (73.4–137) | 1525 (1196–1930) | 40.6 (31.3–51.4) | −61.8 (−74 to −41.8) |
| Central Latin America | 7771 (7160–8525) | 7.2 (6.6–7.9) | 17576 (15512–19758) | 6.6 (5.8–7.4) | −7.9 (−20.8 to 5.1) |
| Colombia | 415 (370–459) | 1.7 (1.6–1.9) | 1323 (1081–1609) | 2.4 (2–2.9) | 38.6 (11.3–68.8) |
| Costa Rica | 277 (234–325) | 13.2 (11.2–15.5) | 691 (589–801) | 12.7 (10.8–14.7) | −3.8 (−23.2 to 18.9) |
| El Salvador | 60 (10–114) | 1.6 (0.3–3.2) | 77 (13–129) | 1.2 (0.2–2.1) | −24.5 (−52.5 to 35.5) |
| Guatemala | 245 (191–297) | 4.9 (3.9–6) | 386 (319–461) | 2.9 (2.4–3.5) | −40.7 (−54.3 to −18.9) |
| Honduras | 214 (49–380) | 8.3 (1.9–15.1) | 492 (92–946) | 6.6 (1.2–12.9) | −20.5 (−53.8 to 26.3) |
| Mexico | 2842 (2596–3086) | 4.9 (4.5–5.3) | 10631 (8982–12237) | 7.7 (6.5–8.9) | 57.9 (31.4–86.1) |
| Nicaragua | 99 (25–184) | 4.7 (1.2–8.8) | 206 (52–343) | 3.5 (0.9–5.9) | −24.8 (−48.6 to 11.4) |
| Panama | 102 (85–122) | 5.8 (4.8–6.8) | 210 (163–265) | 4.8 (3.7–6) | −17.4 (−38.8 to 12.7) |
| Venezuela (Bolivarian Republic of) | 3517 (3093–4109) | 29 (25.6–33.6) | 3561 (2552–4644) | 11.8 (8.5–15.4) | −59.4 (−71.1 to −44.7) |
| Central Sub-Saharan Africa | 97 (54–236) | 0.3 (0.2–0.8) | 210 (115–595) | 0.2 (0.1–0.7) | −17.7 (−31.4 to 2.1) |
| Angola | 20 (11–51) | 0.3 (0.2–0.9) | 44 (25–124) | 0.2 (0.1–0.7) | −27.7 (−43.3 to −9.8) |
| Central African Republic | 5 (3–12) | 0.3 (0.2–0.8) | 9 (5–24) | 0.3 (0.1–0.7) | −18 (−36.8 to 7.9) |
| Congo | 4 (2–10) | 0.2 (0.1–0.7) | 7 (4–22) | 0.2 (0.1–0.5) | −28.9 (−48.4 to −10.5) |
| Democratic Republic of the Congo | 66 (36–153) | 0.3 (0.2–0.7) | 146 (78–415) | 0.3 (0.1–0.8) | −12.5 (−29.2 to 10.9) |
| Equatorial Guinea | 1 (0–2) | 0.3 (0.2–0.8) | 1 (1–4) | 0.1 (0.1–0.4) | −53.4 (−66.9 to −37.6) |
| Gabon | 2 (1–5) | 0.3 (0.2–0.7) | 2 (1–6) | 0.1 (0.1–0.4) | −45.8 (−58.4 to −26.5) |
| East Asia | 22432 (9942–47615) | 2.1 (1–4.4) | 69750 (16482–109077) | 3.6 (0.8–5.6) | 71.5 (−33.5 to 206.2) |
| China | 18890 (6933–43387) | 1.8 (0.7–4.1) | 64746 (12595–102876) | 3.4 (0.7–5.4) | 90.2 (−34.4 to 274.9) |
| Democratic People's Republic of Korea | 948 (363–1878) | 4.9 (1.8–9.7) | 1880 (614–3792) | 5.9 (1.9–11.7) | 20.7 (−36.2 to 112.1) |
| Taiwan (Province of China) | 2595 (2398–2786) | 14 (13–15) | 3124 (2779–3503) | 9.7 (8.6–11) | −30.6 (−39.7 to −19.9) |
| Eastern Europe | 883100 (816272–951221) | 339.3 (313.7–364.9) | 1426959 (1272059–1567982) | 510.5 (456.7–560.3) | 50.4 (34.6–67.7) |
| Belarus | 35635 (28541–43747) | 300.3 (240.1–368.1) | 32068 (25364–39907) | 262.9 (207.5–327.9) | −12.4 (−38.6 to 20.9) |
| Estonia | 4597 (3479–5636) | 251.7 (188.9–308.6) | 2549 (2166–2993) | 130.4 (111.2–152.7) | −48.2 (−59.3 to −33.7) |
| Latvia | 9460 (8043–11082) | 301.2 (256.6–351.6) | 14256 (11943–16704) | 575.3 (485–671.4) | 91 (59.7–128.8) |
| Lithuania | 5565 (4297–6803) | 135.1 (104.2–165.5) | 5707 (4730–6736) | 146.1 (121.9–173.8) | 8.1 (−15.6 to 40.1) |
| Republic of Moldova | 2641 (2289–3071) | 57.2 (49.5–66.5) | 7552 (6181–8948) | 142.4 (116.9–168.5) | 149.1 (108–199.2) |
| Russian Federation | 509433 (498574–519401) | 295.9 (289.3–301.8) | 1057433 (947409–1161649) | 542.4 (488.7–594.2) | 83.3 (64.8–100.4) |
| Ukraine | 315769 (259839–371806) | 505.3 (416.5–595.6) | 307394 (216929–412467) | 521.2 (368.5–699.3) | 3.2 (−30.3 to 50.4) |
| Eastern Sub-Saharan Africa | 377 (224–671) | 0.3 (0.2–0.6) | 861 (501–1620) | 0.3 (0.2–0.6) | −8.3 (−19.4 to 2.5) |
| Burundi | 12 (7–22) | 0.3 (0.2–0.7) | 27 (15–49) | 0.3 (0.2–0.6) | −5.7 (−25.5 to 11.8) |
| Comoros | 1 (1–2) | 0.3 (0.2–0.7) | 2 (1–3) | 0.3 (0.1–0.5) | −17.5 (−33.8 to −3.2) |
| Djibouti | 1 (1–2) | 0.3 (0.2–0.6) | 3 (2–6) | 0.3 (0.2–0.6) | −10.5 (−24.5 to 3.4) |
| Eritrea | 7 (4–13) | 0.3 (0.2–0.6) | 13 (7–24) | 0.2 (0.1–0.5) | −15.2 (−36.3 to 3.6) |
| Ethiopia | 106 (62–177) | 0.3 (0.2–0.6) | 252 (148–432) | 0.3 (0.2–0.5) | −2.7 (−22.1 to 10.4) |
| Kenya | 39 (24–66) | 0.3 (0.2–0.5) | 97 (57–164) | 0.2 (0.1–0.4) | −11.5 (−18.6 to −3.4) |
| Madagascar | 29 (16–64) | 0.4 (0.2–0.9) | 69 (39–172) | 0.3 (0.2–0.9) | −11.6 (−29.4 to 7.3) |
| Malawi | 17 (10–30) | 0.3 (0.2–0.5) | 34 (19–62) | 0.2 (0.1–0.5) | −5.1 (−22 to 12.8) |
| Mozambique | 24 (15–43) | 0.3 (0.2–0.5) | 51 (30–92) | 0.3 (0.2–0.5) | −6.4 (−21.3 to 13) |
| Rwanda | 15 (8–27) | 0.3 (0.2–0.6) | 28 (17–51) | 0.3 (0.2–0.5) | −14.6 (−34.7 to 4.1) |
| Somalia | 15 (9–30) | 0.3 (0.2–0.7) | 37 (20–71) | 0.3 (0.1–0.6) | −13.7 (−32.2 to 5.5) |
| South Sudan | 13 (8–24) | 0.3 (0.2–0.7) | 18 (11–36) | 0.3 (0.2–0.6) | −17.8 (−30.1 to −3.9) |
| Uganda | 33 (19–59) | 0.3 (0.2–0.6) | 76 (44–129) | 0.3 (0.2–0.5) | −15.7 (−33.1 to 1) |
| United Republic of Tanzania | 52 (30–96) | 0.3 (0.2–0.6) | 111 (61–214) | 0.3 (0.1–0.6) | −13.8 (−30.6 to 0.9) |
| Zambia | 14 (8–23) | 0.3 (0.2–0.5) | 43 (21–132) | 0.3 (0.2–1.1) | 17.8 (−15.5 to 118) |
| High-income Asia Pacific | 23591 (21977–25712) | 11.5 (10.7–12.5) | 13652 (12386–14806) | 4.5 (4.1–4.8) | −61 (−64.7 to −56.6) |
| Brunei Darussalam | 59 (20–93) | 33.1 (11–51.2) | 90 (30–159) | 18.5 (6.2–33.1) | −44.1 (−61.9 to −15.2) |
| Japan | 22951 (21385–24988) | 14.3 (13.3–15.5) | 13088 (12063–14103) | 6.4 (5.9–6.9) | −55.4 (−59.7 to −50.9) |
| Republic of Korea | 392 (79–684) | 1 (0.2–1.7) | 394 (102–913) | 0.5 (0.1–1.1) | −49.2 (−68.8 to −10.3) |
| Singapore | 189 (171–208) | 6.7 (6.1–7.3) | 80 (69–91) | 1 (0.8–1.1) | −85.8 (−87.9 to −83.3) |
| High-income North America | 140939 (131197–149688) | 43.6 (40.6–46.2) | 177118 (166935–187218) | 32.9 (31–34.7) | −24.6 (−30.9 to −17.3) |
| Canada | 7684 (6634–8805) | 24.5 (21.1–28) | 11004 (9971–12147) | 19.1 (17.4–21.1) | −21.9 (−34.9 to −6.8) |
| Greenland | 16 (4–31) | 32.4 (7.7–64.2) | 22 (4–38) | 29.8 (5.1–49.6) | −8.1 (−49.1 to 49.6) |
| United States of America | 133235 (124174–141387) | 45.7 (42.6–48.4) | 166089 (155916–176013) | 34.5 (32.4–36.5) | −24.5 (−31.3 to −17.2) |
| North Africa and Middle East | 3329 (825–5912) | 1.7 (0.4–3.2) | 5724 (1685–10424) | 1.1 (0.3–2) | −36.5 (−53.4 to −11.6) |
| Afghanistan | 289 (61–599) | 3.9 (0.8–8.3) | 356 (88–745) | 2.7 (0.7–7.2) | −32.4 (−62 to 28) |
| Algeria | 336 (73–639) | 2.5 (0.6–5) | 616 (172–1275) | 1.6 (0.5–3.4) | −35.7 (−61.4 to 15.7) |
| Bahrain | 15 (4–24) | 6.7 (1.5–10.3) | 39 (14–93) | 3.4 (1.1–6.8) | −49 (−68.1 to −5.7) |
| Egypt | 23 (7–42) | 0.1 (0–0.1) | 26 (9–55) | 0 (0–0.1) | −50.7 (−74.2 to −8.8) |
| Iran (Islamic Republic of) | 664 (138–1340) | 2.1 (0.5–4.3) | 1189 (256–2208) | 1.3 (0.3–2.6) | −37.9 (−58.8 to −10.1) |
| Iraq | 25 (3–50) | 0.3 (0–0.5) | 49 (5–93) | 0.2 (0–0.3) | −33.9 (−67.8 to 46.3) |
| Jordan | 4 (0–6) | 0.2 (0–0.4) | 9 (2–14) | 0.1 (0–0.2) | −59.6 (−76.1 to −17.5) |
| Kuwait | 89 (66–119) | 8.3 (6.5–10.9) | 143 (96–197) | 3.4 (2.3–4.5) | −59.3 (−71.4 to −44.4) |
| Lebanon | 4 (1–9) | 0.2 (0–0.4) | 5 (1–7) | 0.1 (0–0.1) | −60.3 (−79.3 to 0.9) |
| Libya | 40 (8–73) | 1.7 (0.4–3.3) | 88 (27–172) | 1.3 (0.4–2.5) | −24.5 (−59.1 to 50.3) |
| Morocco | 426 (91–863) | 2.7 (0.6–5.4) | 719 (202–1661) | 2 (0.6–4.6) | −25.1 (−55.3 to 30) |
| Oman | 6 (1–12) | 0.6 (0.1–1.2) | 9 (2–22) | 0.3 (0.1–0.7) | −54.8 (−81 to 24.9) |
| Palestine | 12 (2–22) | 1.4 (0.3–2.6) | 23 (6–45) | 0.9 (0.2–1.6) | −37.4 (−65.4 to 6.9) |
| Qatar | 4 (1–8) | 2 (0.4–4) | 20 (6–61) | 1.1 (0.3–3.3) | −42.3 (−73.1 to 26.9) |
| Saudi Arabia | 220 (34–379) | 2.9 (0.5–4.9) | 547 (105–951) | 1.7 (0.3–2.9) | −42.2 (−71.4 to 15.3) |
| Sudan | 340 (76–680) | 3.2 (0.7–6.6) | 443 (130–932) | 1.8 (0.5–3.9) | −44.1 (−68.9 to 7.6) |
| Syrian Arab Republic | 186 (41–334) | 3 (0.7–5.3) | 302 (77–598) | 2.1 (0.6–4.2) | −29.5 (−66.6 to 35.4) |
| Tunisia | 137 (26–253) | 2.5 (0.5–4.8) | 235 (57–449) | 1.8 (0.4–3.5) | −29 (−57 to 21.6) |
| Turkey | 244 (53–572) | 0.6 (0.1–1.4) | 359 (78–707) | 0.4 (0.1–0.7) | −39.2 (−68.4 to 20.3) |
| United Arab Emirates | 37 (9–76) | 4.1 (1–8.1) | 123 (40–314) | 1.8 (0.5–4.2) | −56.8 (−77.4 to −21.8) |
| Yemen | 226 (45–493) | 3.9 (0.8–8.9) | 420 (107–913) | 2.4 (0.6–5.3) | −38.5 (−66.4 to 11.4) |
| Oceania | 116 (14–237) | 2.5 (0.3–5) | 206 (34–420) | 1.9 (0.3–3.9) | −26.8 (−56.7 to 18.5) |
| American Samoa | 3 (1–5) | 8.9 (2.6–15.2) | 2 (1–5) | 4.1 (1.4–9.1) | −54.4 (−73.6 to −7.4) |
| Cook Islands | 0 (0–0) | 0 (0–0) | 0 (0–0) | 0 (0–0) | −66.5 (−84.7 to −22) |
| Fiji | 3 (0–5) | 0.5 (0.1–0.9) | 4 (1–7) | 0.4 (0.1–0.7) | −20.3 (−51.1 to 29.3) |
| Guam | 5 (2–11) | 5 (1.6–10.9) | 6 (1–12) | 3.3 (0.8–6.3) | −34.3 (−57.8 to −4.1) |
| Kiribati | 2 (1–4) | 5 (1.2–8.9) | 4 (1–8) | 4.7 (1.2–8.7) | −5.6 (−45.7 to 60.3) |
| Marshall Islands | 1 (0–1) | 2.9 (0.3–6) | 1 (0–2) | 2.1 (0.3–4.4) | −28.6 (−59.8 to 26.4) |
| Micronesia (Federated States of) | 2 (0–4) | 3.4 (0.4–6.7) | 2 (0–4) | 2.3 (0.3–4.7) | −32.9 (−60.6 to 21.8) |
| Nauru | 0 (0–0) | 3.4 (0.5–6.9) | 0 (0–0) | 2.6 (0.4–4.8) | −24.8 (−55 to 38.1) |
| Niue | 0 (0–0) | 2.2 (0.3–4) | 0 (0–0) | 1.7 (0.2–3) | −24.4 (−57.5 to 34.5) |
| Northern Mariana Islands | 2 (1–4) | 7.6 (1.7–13.9) | 3 (1–8) | 4.2 (1.2–12.2) | −44.6 (−71.1 to 32.5) |
| Palau | 0 (0–0) | 1.6 (0.2–3) | 0 (0–1) | 1.1 (0.2–2.2) | −27.9 (−57.7 to 30.7) |
| Papua New Guinea | 79 (7–176) | 2.7 (0.3–5.9) | 157 (24–338) | 1.9 (0.3–4.3) | −28.1 (−63.6 to 36) |
| Samoa | 3 (0–6) | 2.5 (0.3–5.3) | 3 (0–6) | 1.7 (0.3–3.2) | −31.9 (−63.2 to 18.1) |
| Solomon Islands | 4 (0–8) | 2.1 (0.2–4.4) | 8 (1–17) | 1.7 (0.3–3.8) | −19.5 (−53 to 48.8) |
| Tokelau | 0 (0–0) | 2.7 (0.4–6) | 0 (0–0) | 2.1 (0.3–3.9) | −23.4 (−57.5 to 50.4) |
| Tonga | 1 (0–2) | 1.5 (0.2–3) | 1 (0–2) | 1.1 (0.2–2.2) | −29.5 (−59.1 to 23.5) |
| Tuvalu | 0 (0–0) | 3.1 (0.4–5.8) | 0 (0–0) | 1.9 (0.3–3.7) | −36.9 (−65.5 to 12.6) |
| Vanuatu | 2 (0–5) | 2.6 (0.4–5.1) | 5 (1–9) | 1.9 (0.3–3.8) | −27.9 (−60.2 to 27.1) |
| South Asia | 25174 (5778–61382) | 3.5 (0.8–8.5) | 47908 (9930–112145) | 2.9 (0.6–6.7) | −18.2 (−45.2 to 19.1) |
| Bangladesh | 2658 (572–6730) | 4.7 (1–11.8) | 4878 (866–13564) | 3.2 (0.6–9.1) | −31.3 (−62.7 to 24.1) |
| Bhutan | 10 (2–31) | 3.2 (0.7–9.8) | 18 (4–58) | 2.6 (0.5–8.3) | −17.3 (−56.4 to 62) |
| India | 20009 (4566–45824) | 3.4 (0.8–7.8) | 37424 (7656–85532) | 2.8 (0.6–6.4) | −17.7 (−46.8 to 21.5) |
| Nepal | 437 (108–1081) | 3.8 (0.9–9.3) | 741 (168–1690) | 2.9 (0.6–6.5) | −24.1 (−56.9 to 59.7) |
| Pakistan | 2059 (488–6137) | 3.2 (0.7–9.4) | 4848 (996–12888) | 3 (0.6–8.1) | −4.4 (−40.2 to 58.3) |
| Southeast Asia | 6602 (1540–11985) | 2 (0.5–3.8) | 13674 (2550–21947) | 1.8 (0.3–2.9) | −6.7 (−40 to 32.6) |
| Cambodia | 93 (24–191) | 1.6 (0.4–3.1) | 230 (43–451) | 1.5 (0.3–2.9) | −4.2 (−51.5 to 66.8) |
| Indonesia | 2194 (469–3930) | 1.6 (0.4–3) | 5104 (828–9265) | 1.8 (0.3–3.1) | 7.1 (−33.5 to 63.4) |
| Lao People's Democratic Republic | 60 (13–123) | 2.3 (0.5–4.6) | 114 (23–214) | 1.8 (0.4–3.3) | −21 (−60.6 to 38.7) |
| Malaysia | 8 (1–15) | 0.1 (0–0.1) | 10 (1–21) | 0 (0–0.1) | −52.3 (−75 to 24.1) |
| Maldives | 3 (1–6) | 2.8 (0.8–5.5) | 7 (2–14) | 1.4 (0.4–2.4) | −49.6 (−81.7 to −5.1) |
| Mauritius | 86 (76–96) | 9.6 (8.6–10.7) | 134 (116–155) | 7.9 (6.8–9.1) | −18.4 (−31.9 to −2.4) |
| Myanmar | 713 (165–1260) | 2.4 (0.6–4.4) | 1139 (206–2086) | 2.1 (0.4–3.7) | −15.8 (−55.1 to 55.4) |
| Philippines | 1393 (301–2327) | 3.2 (0.7–5.9) | 2900 (612–5525) | 2.8 (0.6–5.4) | −13.3 (−42.5 to 16.5) |
| Seychelles | 1 (0–2) | 1.8 (0.3–3.2) | 2 (0–4) | 1.5 (0.2–2.8) | −19.1 (−53.8 to 32) |
| Sri Lanka | 971 (203–2221) | 7.1 (1.5–16.8) | 720 (142–1510) | 2.8 (0.6–5.7) | −60.7 (−82.1 to −24.9) |
| Thailand | 238 (71–585) | 0.5 (0.2–1.2) | 974 (123–1794) | 1 (0.1–1.9) | 103.5 (−46.7 to 306.7) |
| Timor-Leste | 10 (2–21) | 2 (0.4–4.4) | 21 (4–47) | 2.1 (0.4–4.8) | 3.9 (−45.7 to 94.4) |
| Viet Nam | 822 (166–1571) | 1.8 (0.4–3.4) | 2301 (362–4528) | 2.1 (0.3–4) | 15.7 (−38.1 to 138.4) |
| Southern Latin America | 17883 (15618–20507) | 38 (33.2–43.6) | 5025 (4485–5557) | 6.1 (5.5–6.8) | −83.9 (−86.2 to −81) |
| Uruguay | 3248 (2827–3655) | 88.9 (77.2–100.1) | 1398 (1235–1588) | 30 (26.3–33.9) | −66.3 (−71.9 to −58.2) |
| Argentina | 13079 (11086–15574) | 40.3 (34.2–47.9) | 2388 (2054–2740) | 4.5 (3.9–5.2) | −88.8 (−90.9 to −86.4) |
| Chile | 1555 (1369–1735) | 14 (12.3–15.7) | 1238 (1063–1428) | 5.2 (4.5–6) | −63 (−69.7 to −55.5) |
| Southern Sub-Saharan Africa | 133 (51–278) | 0.3 (0.1–0.6) | 172 (72–469) | 0.3 (0.1–0.6) | −18.1 (−42.4 to 29) |
| Botswana | 3 (2–7) | 0.3 (0.2–0.8) | 5 (3–10) | 0.2 (0.1–0.5) | −37.8 (−55.2 to −12.4) |
| Eswatini | 2 (1–3) | 0.3 (0.1–0.6) | 2 (1–4) | 0.2 (0.1–0.4) | −32.1 (−50.9 to −6.2) |
| Lesotho | 3 (2–5) | 0.2 (0.1–0.4) | 4 (2–7) | 0.2 (0.1–0.4) | −12.3 (−39.7 to 25.1) |
| Namibia | 3 (2–6) | 0.3 (0.2–0.6) | 5 (3–10) | 0.2 (0.1–0.5) | −25.9 (−46.6 to −0.6) |
| South Africa | 78 (26–105) | 0.2 (0.1–0.3) | 55 (23–82) | 0.1 (0–0.2) | −43.9 (−55.4 to −26.7) |
| Zimbabwe | 44 (13–172) | 0.7 (0.2–2.6) | 101 (28–366) | 0.9 (0.2–2.9) | 16.6 (−32.7 to 98.6) |
| Tropical Latin America | 67688 (62671–74420) | 57.5 (53.3–63.4) | 44412 (41533–47435) | 16.8 (15.7–18) | −70.8 (−73.8 to −67.6) |
| Brazil | 67478 (62542–74130) | 58.7 (54.4–64.7) | 44114 (41180–47075) | 17.1 (16–18.3) | −70.8 (−73.8 to −67.6) |
| Paraguay | 210 (39–359) | 7.6 (1.4–13) | 298 (78–637) | 4.4 (1.2–9.5) | −41.9 (−64.3 to 9.3) |
| Western Europe | 218705 (197714–237175) | 42.9 (38.9–46.5) | 127836 (116945–137644) | 17.6 (16.2–18.9) | −58.9 (−64.2 to −53.7) |
| Andorra | 23 (10–38) | 39 (16.9–64.1) | 28 (12–46) | 19.6 (8.3–31.7) | −49.6 (−71 to −12.9) |
| Austria | 12939 (11961–13947) | 116.1 (108.2–124.3) | 3850 (3446–4234) | 26.3 (23.7–28.7) | −77.4 (−79.8 to −74.8) |
| Belgium | 2264 (2077–2485) | 16.9 (15.5–18.5) | 2633 (2347–2922) | 14 (12.5–15.5) | −17.2 (−27.4 to −6.4) |
| Cyprus | 123 (33–230) | 15.5 (4.2–28.7) | 134 (37–259) | 7 (1.9–13.5) | −54.7 (−73.1 to −22.4) |
| Denmark | 1518 (1334–1705) | 22.1 (19.3–24.8) | 1135 (997–1293) | 11.5 (10.1–13) | −48.1 (−56.9 to −37.6) |
| Finland | 7735 (6754–8772) | 121.8 (106.3–138.3) | 6216 (5606–6823) | 70.9 (63.9–77.9) | −41.8 (−51.8 to −30.3) |
| France | 10723 (9906–11532) | 15 (13.8–16.2) | 13185 (11318–15170) | 12.5 (10.8–14.3) | −16.5 (−28.9 to −4.3) |
| Germany | 145138 (125862–161999) | 129.1 (112.3–144.4) | 63577 (56920–69886) | 41.6 (37.4–45.5) | −67.8 (−72.9 to −62) |
| Greece | 552 (439–660) | 4 (3.2–4.8) | 556 (474–650) | 3.2 (2.8–3.8) | −20 (−37 to 5) |
| Iceland | 29 (26–32) | 10.8 (9.7–11.8) | 26 (22–30) | 5.3 (4.5–6.1) | −50.4 (−58.7 to −41.6) |
| Ireland | 1180 (1091–1270) | 31 (28.8–33.3) | 939 (782–1111) | 13.2 (11–15.6) | −57.4 (−64.6 to −49.3) |
| Israel | 122 (104–139) | 2.6 (2.3–3) | 168 (144–195) | 1.5 (1.3–1.8) | −41.4 (−51.9 to −28) |
| Italy | 3316 (3153–3481) | 4.3 (4.1–4.6) | 3157 (2572–3703) | 3.3 (2.7–3.9) | −23.7 (−38.1 to −10.2) |
| Luxembourg | 177 (162–194) | 35.1 (32.3–38.4) | 135 (114–158) | 13.7 (11.5–16) | −61 (−67.3 to −53.5) |
| Malta | 58 (52–65) | 13.8 (12.5–15.4) | 41 (35–48) | 5.4 (4.5–6.4) | −60.8 (−67.5 to −52.4) |
| Monaco | 49 (20–74) | 88.6 (38.8–131.7) | 34 (14–51) | 46.9 (19.7–70.4) | −47.1 (−67.3 to −18.7) |
| Netherlands | 7747 (7242–8311) | 41.3 (38.6–44.2) | 3975 (3471–4455) | 13.6 (11.9–15.1) | −67.2 (−71.3 to −62.9) |
| Norway | 766 (728–805) | 14.8 (14–15.6) | 503 (456–561) | 6.4 (5.8–7.1) | −56.9 (−60.9 to −51.8) |
| Portugal | 1556 (1431–1691) | 12.5 (11.6–13.6) | 1775 (1509–2064) | 9.7 (8.2–11.3) | −22.4 (−34.8 to −8.3) |
| San Marino | 6 (2–11) | 18 (6.3–33.5) | 6 (2–11) | 9.7 (3.4–17.6) | −46.4 (−69.4 to −7.1) |
| Spain | 6931 (6462–7463) | 14.1 (13.2–15.1) | 6682 (5699–7786) | 8.6 (7.3–9.9) | −39.2 (−48.2 to −28.8) |
| Sweden | 1201 (1091–1321) | 10.7 (9.7–11.8) | 2702 (2265–3193) | 17.1 (14.3–20.3) | 60 (33–94.1) |
| Switzerland | 3530 (3085–4011) | 38.1 (33.3–43.4) | 2221 (1816–2612) | 14.6 (12–17.1) | −61.6 (−71.5 to −51.5) |
| United Kingdom | 10844 (10578–11117) | 15.3 (14.9–15.7) | 14044 (13232–14848) | 15.1 (14.2–15.9) | −1.6 (−7.7 to 4.7) |
| Western Sub-Saharan Africa | 5463 (881–9932) | 5.4 (0.9–9.7) | 5984 (1532–14383) | 2.3 (0.6–5.5) | −57.6 (−72.1 to −29.1) |
| Benin | 75 (14–176) | 3.5 (0.6–8.1) | 117 (33–272) | 1.8 (0.4–4.2) | −49.4 (−76.1 to 3.2) |
| Burkina Faso | 273 (36–673) | 5.6 (0.8–13.5) | 288 (71–717) | 2.6 (0.6–6.5) | −53.8 (−79 to 0.7) |
| Cabo Verde | 2 (1–3) | 0.7 (0.3–1.4) | 4 (2–6) | 0.7 (0.4–1.1) | −4.3 (−41.9 to 59.5) |
| Cameroon | 218 (35–503) | 4.1 (0.6–9.5) | 370 (98–844) | 2.2 (0.5–4.9) | −47.1 (−75.9 to 8.4) |
| Chad | 118 (20–293) | 4 (0.7–9.9) | 168 (43–427) | 2.3 (0.6–5.9) | −42.5 (−72.7 to 16.1) |
| Côte d'Ivoire | 285 (40–579) | 5.2 (0.8–10.7) | 371 (90–764) | 2.3 (0.5–4.9) | −54.9 (−79.4 to −6) |
| Gambia | 21 (3–49) | 5.1 (0.7–11.8) | 28 (7–63) | 2.3 (0.6–5.4) | −54.3 (−79.4 to −1.1) |
| Ghana | 1132 (183–2453) | 14.3 (2.5–31.7) | 1715 (323–4091) | 7.9 (1.5–18.6) | −44.9 (−74.6 to 6.5) |
| Guinea | 120 (22–281) | 3.5 (0.7–8) | 136 (35–335) | 2 (0.5–5.2) | −41.6 (−71.1 to 17.9) |
| Guinea-Bissau | 25 (4–61) | 5.5 (0.8–13.2) | 24 (6–55) | 2.4 (0.6–5.4) | −56 (−79.9 to 0.2) |
| Liberia | 52 (8–123) | 4.2 (0.6–10.3) | 65 (17–146) | 2.1 (0.5–4.7) | −50.8 (−78.8 to 8.7) |
| Mali | 131 (23–329) | 2.8 (0.5–6.8) | 161 (52–413) | 1.4 (0.4–3.7) | −50.3 (−75.5 to −0.4) |
| Mauritania | 40 (7–91) | 3.6 (0.6–8.1) | 40 (12–100) | 1.5 (0.4–3.9) | −56.4 (−80.9 to −7) |
| Niger | 142 (23–356) | 4.4 (0.7–10.5) | 198 (54–479) | 1.9 (0.5–4.6) | −56.3 (−79.4 to −12.9) |
| Nigeria | 2486 (401–4963) | 4.9 (0.8–9.7) | 1890 (550–4724) | 1.5 (0.4–3.7) | −69.9 (−83 to −35.5) |
| Sao Tome and Principe | 2 (0–4) | 3 (0.5–6.5) | 3 (1–6) | 1.8 (0.5–4.2) | −41.1 (−72.3 to 24.2) |
| Senegal | 164 (25–357) | 4.5 (0.7–9.8) | 180 (46–475) | 1.9 (0.5–5.1) | −57.6 (−81.7 to −1) |
| Sierra Leone | 113 (17–264) | 5.2 (0.8–12.6) | 111 (27–261) | 2.3 (0.5–5.8) | −55.6 (−80 to 3.8) |
| Togo | 63 (10–144) | 4.1 (0.6–9.2) | 115 (28–268) | 2.2 (0.5–5) | −46 (−75.1 to 14) |
