# Supplementary material for: Global, regional, and national burden of cardiomyopathy (including alcoholic cardiomyopathy and others) from 1990 to 2021: An analysis of data from the global burden of disease study 2021 and forecast to 2040
Source: PLoS One. 2026 Jan 30;21(1):e0341687. doi: 10.1371/journal.pone.0341687 (PMC12858021; doi:10.1371/journal.pone.0341687)
Supplement: S13 Table — (DOCX) [file pone.0341687.s024.docx]

**S13 Table.** **1990–2021 Global and national prevalence trends in other cardiomyopathy burden.**

| location_name | Number_1990 | ASR per 100,000_1990 | Number_2021 | ASR per 100,000_2021 | Percentage change in the ASRs per 100,000 |
| --- | --- | --- | --- | --- | --- |
| Global | 2285259 (1897990–2686424) | 49.6 (41.1–57.6) | 4223932 (3417357–5034258) | 53.3 (43.6–63.8) | 7.6 (1–14.6) |
| Andean Latin America | 10657 (8667–13166) | 30.2 (24.9–36.2) | 21870 (17302–26471) | 34.4 (27.4–41.6) | 14 (1.5–26.3) |
| Bolivia (Plurinational State of) | 1610 (1311–2005) | 26.6 (22–31.7) | 3978 (3150–4987) | 35.7 (28.6–43.9) | 34.4 (19.8–50.4) |
| Ecuador | 2818 (2316–3514) | 29 (24–34.6) | 5962 (4712–7368) | 34.1 (26.9–41.6) | 17.6 (2.4–34.3) |
| Peru | 6228 (4933–7657) | 31.7 (25.8–37.6) | 11930 (9327–14536) | 34.1 (26.7–41.6) | 7.6 (−5.1 to 21.1) |
| Australasia | 21299 (17762–24819) | 106.6 (89.5–124.7) | 36371 (28887–43447) | 103.6 (83.1–125.5) | −2.8 (−11.3 to 7.3) |
| Australia | 18422 (15234–21492) | 110.7 (92.4–129) | 31490 (24936–37723) | 106.3 (84.8–129.2) | −4 (−13.5 to 6.6) |
| New Zealand | 2876 (2368–3432) | 86.6 (71.5–103.7) | 4881 (3851–6062) | 89.5 (71.9–110.7) | 3.4 (−6.6 to 13.6) |
| Caribbean | 15264 (12490–18263) | 45.6 (37.3–54.1) | 25974 (21014–31004) | 54 (43.6–64.4) | 18.5 (10.3–26.7) |
| Antigua and Barbuda | 28 (23–34) | 46.7 (38.8–56.1) | 55 (44–66) | 61.4 (50.4–73.6) | 31.3 (18.3–46.2) |
| Bahamas | 148 (120–182) | 61.4 (51.3–73.5) | 215 (173–262) | 61.8 (49.8–74.9) | 0.7 (−9.1 to 11.3) |
| Barbados | 195 (161–231) | 71.8 (59.3–84.3) | 286 (227–351) | 76.5 (62.5–92.2) | 6.5 (−2.6 to 17.5) |
| Belize | 108 (88–134) | 59.8 (49.6–70.7) | 240 (195–296) | 62.5 (50.7–75.3) | 4.6 (−5.3 to 16.8) |
| Bermuda | 43 (36–51) | 74.8 (62.1–89.1) | 98 (76–123) | 106.5 (87.3–128.4) | 42.3 (28.7–58.1) |
| Cuba | 3936 (3201–4694) | 37.7 (30.5–44.6) | 7856 (6282–9673) | 62.9 (50.7–76.4) | 66.8 (48.7–83.4) |
| Dominica | 71 (59–84) | 100.3 (84.9–116.9) | 81 (66–97) | 116.9 (97.8–137.6) | 16.5 (6.4–27.3) |
| Dominican Republic | 3098 (2522–3882) | 43.6 (35.7–52.4) | 5828 (4656–7094) | 54.9 (43.8–66.4) | 25.9 (14.2–39.2) |
| Grenada | 53 (44–65) | 60.6 (49.3–72.1) | 76 (60–91) | 76.2 (61.4–91.1) | 25.8 (15–40.4) |
| Guyana | 343 (275–427) | 45.1 (36.8–54.4) | 296 (236–356) | 42.9 (34.7–51.5) | −5 (−15.8 to 6.2) |
| Haiti | 1553 (1265–1893) | 31.7 (26–37.6) | 4541 (3644–5553) | 42.7 (35–50.3) | 34.4 (21.6–49.9) |
| Jamaica | 1347 (1108–1612) | 57.5 (47.9–68.2) | 1782 (1475–2154) | 63.7 (52.7–76.3) | 10.8 (1.1–21.7) |
| Puerto Rico | 2984 (2447–3554) | 82.8 (68.4–98.2) | 2459 (1907–3163) | 53.3 (42.4–65.6) | −35.6 (−43.8 to −26.8) |
| Saint Kitts and Nevis | 39 (32–47) | 93.5 (77.8–110.4) | 58 (47–69) | 107.2 (86.7–125.5) | 14.6 (4.6–25.2) |
| Saint Lucia | 95 (79–114) | 77.3 (64.2–90.1) | 145 (118–175) | 76.7 (63.1–91.1) | −0.7 (−10.2 to 10.9) |
| Saint Vincent and the Grenadines | 27 (21–33) | 24.3 (19.7–29.3) | 33 (26–39) | 28.9 (23.4–34.9) | 18.8 (7.3–30.9) |
| Suriname | 194 (159–237) | 53 (43.7–64.3) | 341 (277–408) | 60.5 (49.2–72.6) | 14.1 (4.4–25.9) |
| Trinidad and Tobago | 409 (336–500) | 35.5 (29.3–42.2) | 623 (502–758) | 41.7 (34.1–50) | 17.4 (5.2–30.4) |
| United States Virgin Islands | 75 (62–89) | 76.6 (64–90.7) | 83 (66–104) | 78.3 (64.7–92.7) | 2.2 (−6.9 to 11.3) |
| Central Asia | 18607 (15302–22601) | 28 (23.2–33.1) | 51461 (42101–60822) | 57.9 (47.6–68.7) | 106.9 (83.8–129.2) |
| Armenia | 926 (753–1138) | 28.4 (23.4–34.5) | 1060 (839–1285) | 34.2 (27–42.3) | 20.4 (1.4–39.2) |
| Azerbaijan | 5012 (4129–6098) | 72.7 (60.6–86.4) | 9499 (7682–11400) | 93.2 (77–110.5) | 28.2 (15.7–40.8) |
| Georgia | 1608 (1324–1921) | 29.2 (24.2–34.8) | 1533 (1165–1922) | 38.2 (30.8–46.4) | 31 (16.3–46.9) |
| Kazakhstan | 4223 (3271–5330) | 26.4 (20.7–33.3) | 24034 (18850–28983) | 135.1 (106.3–162.9) | 411 (293.6–548.9) |
| Kyrgyzstan | 195 (152–249) | 4.2 (3.3–5.3) | 901 (717–1148) | 13.7 (11.2–17.1) | 226.3 (183–273.3) |
| Mongolia | 759 (614–920) | 43.3 (35.3–51.8) | 1889 (1542–2265) | 66.8 (53.7–81.5) | 54.2 (35–74) |
| Tajikistan | 781 (608–994) | 14.1 (11.4–17.2) | 1970 (1515–2537) | 19.3 (15.2–24.3) | 37.1 (21.4–52.3) |
| Turkmenistan | 2815 (2329–3418) | 84.3 (69.9–99.7) | 4084 (3367–4901) | 83.7 (69.4–100.1) | −0.7 (−9.8 to 9.2) |
| Uzbekistan | 2289 (1809–2885) | 10.5 (8.4–12.7) | 6491 (5125–7877) | 19.7 (15.8–23.7) | 87.7 (63.6–109.8) |
| Central Europe | 98465 (80347–118175) | 79.5 (65.3–95) | 155384 (122246–194150) | 93.1 (76.5–112.4) | 17.1 (8.1–26.9) |
| Albania | 1699 (1369–2054) | 67.5 (53–83.1) | 2587 (1980–3295) | 82 (65.3–102.6) | 21.5 (6.5–39) |
| Bosnia and Herzegovina | 3762 (3076–4504) | 99.4 (80.4–119.6) | 4988 (3776–6324) | 122.9 (98.6–150.1) | 23.7 (10.3–36.7) |
| Bulgaria | 1143 (890–1419) | 13.4 (10.7–16.7) | 1510 (1154–2014) | 18.6 (14.9–23.2) | 38.6 (24.9–56.3) |
| Croatia | 1159 (893–1469) | 25.4 (19.8–31.7) | 1759 (1237–2394) | 27.3 (21.1–34.5) | 7.7 (−9.2 to 25.6) |
| Czechia | 3241 (2586–4001) | 31 (24.9–37.4) | 6995 (5165–9177) | 49.2 (38.5–60.8) | 58.9 (36.6–82.5) |
| Hungary | 4368 (3304–5545) | 41 (32.3–51.4) | 5337 (3913–7136) | 46.7 (35.9–59) | 13.9 (−1.8 to 29.8) |
| Montenegro | 909 (749–1077) | 155.6 (127.5–185.7) | 1091 (872–1361) | 159.8 (131–194.1) | 2.7 (−6.2 to 14.2) |
| North Macedonia | 1686 (1376–2012) | 94.1 (76.2–112.3) | 2638 (2083–3258) | 119.7 (98.4–145.1) | 27.3 (13.8–41.6) |
| Poland | 48613 (39493–59027) | 126.1 (102.7–150.8) | 85764 (66734–107670) | 144.1 (117.7–178.8) | 14.2 (1.2–29) |
| Romania | 18545 (13921–23757) | 80.8 (61.7–103.3) | 23861 (16451–31944) | 85 (64.6–106.2) | 5.3 (−6.9 to 19.1) |
| Serbia | 6066 (4800–7366) | 69.4 (54.6–86) | 9247 (6962–11979) | 88.4 (71.3–106.9) | 27.5 (13.7–42.6) |
| Slovakia | 1680 (1365–2025) | 32.4 (26.3–39.4) | 3131 (2439–3918) | 51 (40.4–62.7) | 57.4 (39.8–77.3) |
| Slovenia | 4019 (2860–5160) | 185.1 (138.9–233.4) | 4215 (3085–5668) | 132.7 (106.8–164.3) | −28.3 (−37.5 to −15.4) |
| Central Latin America | 57005 (46482–70063) | 34.9 (28.9–41.3) | 95571 (77117–116363) | 39.2 (31.9–47.7) | 12.4 (3.7–21) |
| Colombia | 14101 (11416–17771) | 45.3 (37.5–55) | 25809 (20172–31772) | 53.3 (42.1–65.4) | 17.8 (2.4–33) |
| Costa Rica | 1957 (1608–2378) | 70.4 (58.6–83.3) | 3243 (2701–3868) | 66.4 (55.8–78.6) | −5.7 (−13.3 to 2.8) |
| El Salvador | 1825 (1466–2302) | 35.2 (28.8–42.5) | 2391 (1920–2921) | 36.9 (29.6–44.9) | 4.8 (−6.6 to 17.1) |
| Guatemala | 3666 (2897–4708) | 40.2 (32.5–48.1) | 6006 (4628–7562) | 39.7 (30.8–48.6) | −1.2 (−14.5 to 13.1) |
| Honduras | 1522 (1237–1907) | 34 (28.3–40.8) | 4143 (3200–5312) | 41.9 (33.3–51.9) | 23.2 (11.7–37.2) |
| Mexico | 20299 (15424–25728) | 22.3 (17.4–27.5) | 36304 (27908–46426) | 29.5 (22.7–37.6) | 32.2 (21–44.2) |
| Nicaragua | 1543 (1243–1972) | 40.3 (33.2–48.5) | 2694 (2171–3277) | 42.9 (35–51.5) | 6.5 (−3.4 to 16.9) |
| Panama | 1620 (1358–1933) | 76.6 (63.9–89.3) | 3766 (3112–4414) | 86.6 (71.8–101.6) | 13.1 (1.2–25.3) |
| Venezuela (Bolivarian Republic of) | 10473 (8441–13059) | 57.4 (47.5–69) | 11215 (9245–13390) | 44 (36.1–52.7) | −23.2 (−30.5 to −15) |
| Central Sub-Saharan Africa | 30397 (22663–40867) | 70.6 (51.7–92.4) | 86229 (63637–116837) | 78.6 (58.6–102.7) | 11.3 (4.1–19.9) |
| Angola | 5558 (4061–7524) | 69.7 (50.4–91.8) | 20313 (14811–27407) | 79.8 (59.3–104.4) | 14.5 (5.3–25) |
| Central African Republic | 1340 (999–1800) | 61.8 (45–80.9) | 2981 (2170–4052) | 67.3 (48.9–88.2) | 8.9 (−0.5 to 18.6) |
| Congo | 1397 (1016–1866) | 71.9 (52.9–94.2) | 3804 (2792–5149) | 81.7 (60.5–105.2) | 13.5 (3.9–23.2) |
| Democratic Republic of the Congo | 21213 (15700–28595) | 71.3 (52–92.4) | 56745 (41575–76856) | 78.5 (58.3–102.8) | 10.1 (1.5–20.7) |
| Equatorial Guinea | 235 (169–316) | 68.6 (50.5–89.1) | 992 (732–1331) | 84 (61.4–109.5) | 22.4 (11.7–34.8) |
| Gabon | 654 (479–863) | 78 (57.2–102.4) | 1394 (1020–1869) | 87.2 (64–113.6) | 11.9 (3.1–22.3) |
| East Asia | 99645 (78590–123303) | 9.4 (7.6–11.4) | 269774 (210535–337384) | 18.1 (14.3–22.7) | 92.9 (77.1–109.3) |
| China | 90973 (71312–113404) | 8.8 (7.1–10.8) | 258098 (200950–322692) | 17.9 (14.1–22.5) | 103 (86.3–120.7) |
| Democratic People's Republic of Korea | 2490 (2008–3103) | 13.4 (10.9–16.6) | 4039 (3162–4982) | 16.7 (13.2–20.8) | 24.6 (13.4–37.3) |
| Taiwan (Province of China) | 6182 (4982–7525) | 34.6 (28.2–42) | 7636 (5885–9637) | 29.6 (23.2–36.2) | −14.5 (−24 to −4.1) |
| Eastern Europe | 77760 (62841–95782) | 38.3 (30.6–47.1) | 150142 (121248–181364) | 73.5 (59.8–90) | 91.7 (75.4–109.2) |
| Belarus | 3394 (2808–4094) | 33.6 (27.8–41.1) | 3952 (3120–4828) | 48.9 (38.2–60.9) | 45.7 (25.2–68.6) |
| Estonia | 701 (576–845) | 44.7 (36.8–53.8) | 754 (587–926) | 56.2 (43.8–69.7) | 25.8 (7.4–44.2) |
| Latvia | 1547 (1262–1843) | 58.3 (48–70.5) | 2356 (1827–2916) | 99.5 (82.1–118.2) | 70.6 (49.3–92.1) |
| Lithuania | 1305 (1071–1588) | 35.8 (29.3–43.3) | 1782 (1348–2243) | 59.1 (45.4–74.7) | 65.2 (41.2–91.5) |
| Republic of Moldova | 910 (731–1100) | 20.8 (16.7–25.2) | 1285 (1040–1553) | 40.2 (32.5–49.5) | 93.3 (71.7–115.9) |
| Russian Federation | 50265 (39840–62490) | 37.9 (29.9–46.8) | 118799 (95247–143603) | 82 (66.6–100.5) | 116.1 (94.3–140.4) |
| Ukraine | 19637 (15611–24029) | 41.1 (32.4–51.4) | 21214 (16861–25914) | 52.2 (40.2–65.8) | 26.9 (14–41.5) |
| Eastern Sub-Saharan Africa | 154906 (119601–197262) | 109.3 (84.5–140.3) | 394350 (306831–496748) | 124.1 (95.6–159.2) | 13.6 (9.8–18.4) |
| Burundi | 3940 (2916–5168) | 94.4 (70.3–123.6) | 10455 (7767–13675) | 107.5 (78.4–139.3) | 13.8 (4.7–24.8) |
| Comoros | 384 (284–511) | 108.6 (79.8–141.2) | 782 (585–994) | 119 (88.7–154) | 9.6 (1.9–18.8) |
| Djibouti | 328 (240–434) | 107.6 (79.1–139.9) | 1250 (927–1587) | 117.9 (87.5–152.4) | 9.6 (0.8–20) |
| Eritrea | 2423 (1790–3213) | 95.2 (70.1–124.4) | 5592 (4112–7314) | 107 (79.9–138.8) | 12.4 (4.4–20.9) |
| Ethiopia | 58788 (47243–72477) | 150.6 (118.8–189.7) | 148652 (121169–182055) | 179.9 (141.3–225.3) | 19.4 (14.1–25.6) |
| Kenya | 16303 (12400–20803) | 109.3 (82–141.2) | 43072 (33256–54103) | 115.5 (86.4–147.4) | 5.6 (1.5–9.9) |
| Madagascar | 9254 (6877–11958) | 103.5 (75.8–134.8) | 24568 (18362–31865) | 113.1 (82.3–146.5) | 9.3 (0.7–19.5) |
| Malawi | 6691 (4966–8712) | 92.8 (68.1–122.7) | 15187 (11469–19722) | 105.5 (78.5–136.7) | 13.7 (4.6–25.6) |
| Mozambique | 7484 (5494–9952) | 74.7 (53.9–100.8) | 18591 (13705–24706) | 83.7 (61–110.5) | 12.1 (2.6–22.8) |
| Rwanda | 5251 (3839–6985) | 98.3 (72.3–129.9) | 11572 (8633–15004) | 109.3 (80.3–141.5) | 11.2 (2.3–20.5) |
| Somalia | 5065 (3830–6794) | 88.8 (66.8–114.8) | 14474 (10572–19408) | 92.9 (69–121.9) | 4.6 (−3.8 to 13.8) |
| South Sudan | 4397 (3254–5798) | 102.4 (74.3–134.6) | 7781 (5750–10103) | 108 (80.5–140.5) | 5.5 (−2.9 to 15.4) |
| Uganda | 9150 (6592–12473) | 74.5 (53.9–97.2) | 25637 (18389–35381) | 82.7 (60.4–110) | 11 (1.2–20.8) |
| United Republic of Tanzania | 19925 (14801–25972) | 102.7 (76–133.6) | 50738 (38221–65838) | 111.9 (83.3–146) | 9 (0–19.4) |
| Zambia | 5410 (4007–7165) | 94 (68.6–122.1) | 15657 (11611–20843) | 108.1 (78.5–142.3) | 15 (5.4–26.1) |
| High-income Asia Pacific | 131384 (108949–155310) | 76.9 (63.7–92.3) | 194343 (157026–238660) | 79.3 (65.8–94.5) | 3.1 (−3.4 to 10) |
| Brunei Darussalam | 159 (129–197) | 64.1 (52.3–77) | 282 (226–344) | 70.1 (56.6–84.7) | 9.3 (−0.3 to 20.4) |
| Japan | 112222 (92690–133691) | 88.1 (73.3–105.5) | 147248 (116508–184685) | 80.9 (67.1–97.5) | −8.2 (−14.7 to −1.5) |
| Republic of Korea | 17612 (13775–21658) | 43.9 (35.2–53.1) | 44468 (35913–53314) | 79.5 (65.7–94.3) | 81.2 (62.1–103.6) |
| Singapore | 1389 (1116–1682) | 50.6 (41.7–59.8) | 2344 (1842–2892) | 37.8 (30.1–45.9) | −25.2 (−33.3 to −16.5) |
| High-income North America | 412782 (337957–485478) | 143.1 (118.6–168.4) | 490330 (405044–594053) | 118 (97.8–141.5) | −17.6 (−24.3 to −11.2) |
| Canada | 26122 (21663–31098) | 96 (79.5–114.3) | 41087 (33136–50590) | 86.3 (71.1–102) | −10.1 (−19.1 to 1.2) |
| Greenland | 70 (58–84) | 135.7 (113.9–160.9) | 78 (64–94) | 145.1 (120.1–175.5) | 6.9 (−2 to 15.4) |
| United States of America | 386580 (315558–456164) | 148.1 (122.1–174.2) | 449157 (368663–545692) | 121.3 (100–146) | −18.1 (−25.5 to −11.1) |
| North Africa and Middle East | 107783 (87935–134254) | 30.1 (25.2–35.8) | 222481 (177916–275039) | 37.1 (30.2–45.4) | 23.3 (14.2–32.7) |
| Afghanistan | 1175 (945–1490) | 11.2 (9.3–13.6) | 5146 (3817–6848) | 15.4 (12.1–19.4) | 38.3 (21.9–55.7) |
| Algeria | 6793 (5315–8799) | 25 (20.3–30.7) | 13926 (10999–17860) | 32.6 (26–41.8) | 30.7 (16.1–45.6) |
| Bahrain | 332 (271–409) | 73.3 (61.5–87.1) | 1148 (938–1394) | 91.7 (76.3–108.4) | 25 (14.4–35.5) |
| Egypt | 13417 (10816–17010) | 22.2 (18.1–27) | 30932 (23784–40309) | 28.1 (22.3–35.3) | 26.5 (11.9–42.7) |
| Iran (Islamic Republic of) | 16926 (12985–21465) | 27.3 (21.9–33.3) | 28117 (21921–35644) | 36.5 (28.3–46.1) | 33.5 (24–44) |
| Iraq | 9370 (7548–11713) | 50.7 (42.5–59.4) | 24292 (19588–29670) | 60.7 (50–72.2) | 19.8 (9.1–31.7) |
| Jordan | 604 (469–788) | 14.3 (11.7–17.7) | 1674 (1301–2141) | 14.1 (11.2–17.6) | −1.3 (−12 to 10.9) |
| Kuwait | 663 (527–842) | 40 (32.2–49) | 821 (642–1025) | 24 (19–30.1) | −39.9 (−46.6 to −31.7) |
| Lebanon | 653 (543–787) | 22.4 (19–26.4) | 1764 (1460–2114) | 33.2 (27.5–40.3) | 47.9 (34.1–62.9) |
| Libya | 1934 (1502–2495) | 37.8 (30.4–46.9) | 2046 (1614–2576) | 36.8 (28.8–46.8) | −2.4 (−13.6 to 8.5) |
| Morocco | 8704 (6901–11306) | 30.3 (24.6–38.1) | 13432 (10435–16824) | 38.6 (30–48.4) | 27.5 (13–44.5) |
| Oman | 1339 (1100–1646) | 70.5 (60.3–82.4) | 3054 (2441–3745) | 75.9 (61.1–92) | 7.7 (−3 to 18.9) |
| Palestine | 1050 (836–1327) | 46.7 (39.2–55.8) | 2984 (2389–3752) | 57.9 (47.6–69.8) | 24.1 (13.7–35.4) |
| Qatar | 289 (237–349) | 76.2 (64.4–90.6) | 1822 (1455–2299) | 81.6 (66.6–97.1) | 7.1 (−3.5 to 18.1) |
| Saudi Arabia | 9504 (7664–11827) | 61.4 (51.5–73.1) | 18779 (15265–22694) | 65.1 (54.1–78.3) | 6.1 (−4.2 to 16.8) |
| Sudan | 3337 (2633–4342) | 15.8 (13–19.4) | 9841 (7279–12878) | 21.5 (16.7–27.4) | 35.8 (18.2–54.5) |
| Syrian Arab Republic | 3896 (2985–5093) | 24.9 (20.1–30.8) | 4511 (3461–5744) | 33.7 (26.1–42.4) | 35.8 (19.1–54.1) |
| Tunisia | 2632 (2066–3331) | 30.2 (24.4–37.1) | 3951 (3123–4867) | 36 (28.2–45.1) | 18.9 (6–31.5) |
| Turkey | 21410 (17384–27084) | 36.7 (30.6–44.5) | 38363 (30573–47606) | 48.9 (38.8–61.4) | 33.1 (17.5–49.3) |
| United Arab Emirates | 1516 (1255–1837) | 91.3 (77.3–106.8) | 7882 (6132–9974) | 103.6 (86–121.8) | 13.5 (3.3–25) |
| Yemen | 2179 (1690–2865) | 14.8 (12.2–17.9) | 7790 (5926–10294) | 21.9 (17.4–27.9) | 47.6 (29.9–65.9) |
| Oceania | 1040 (820–1308) | 20.7 (16.5–25.2) | 2685 (2081–3399) | 23.9 (18.5–30.1) | 15.9 (6.9–25.9) |
| American Samoa | 24 (19–30) | 57.4 (46.1–69.8) | 27 (20–33) | 58.4 (45.2–71.3) | 1.6 (−8.2 to 12.9) |
| Cook Islands | 1 (1–2) | 6.7 (5.2–8.7) | 1 (1–2) | 8.3 (6.1–11.3) | 23.5 (7.9–40.3) |
| Fiji | 97 (75–123) | 15.2 (12–18.7) | 158 (122–198) | 19.1 (15–24.1) | 26.3 (14.3–39.8) |
| Guam | 49 (39–61) | 39.5 (31.9–48.1) | 64 (50–78) | 40.3 (31.2–50.1) | 2.1 (−7.2 to 12.3) |
| Kiribati | 8 (7–11) | 17.2 (13.8–21.4) | 17 (13–21) | 19 (15.1–24.1) | 10.2 (−3.1 to 23.9) |
| Marshall Islands | 8 (6–11) | 20.5 (16.3–25.3) | 11 (8–14) | 22.9 (17.9–28.7) | 11.9 (1.5–22.5) |
| Micronesia (Federated States of) | 19 (15–25) | 21.7 (17.3–26.6) | 22 (17–28) | 25 (19.7–31.4) | 15.2 (3.5–27.5) |
| Nauru | 2 (1–2) | 20.9 (16.5–25.9) | 2 (2–3) | 23.9 (18.5–30.2) | 14.3 (2–27.4) |
| Niue | 1 (1–1) | 27.3 (22–33.3) | 0 (0–1) | 29.1 (22.7–36.1) | 6.3 (−3.1 to 17) |
| Northern Mariana Islands | 15 (12–19) | 45.5 (36.5–55.9) | 21 (16–26) | 47.6 (38–58.8) | 4.6 (−5.9 to 15.6) |
| Palau | 3 (2–4) | 22.4 (17.6–27.2) | 4 (3–5) | 24.7 (19.5–31.3) | 10.6 (0.7–21.4) |
| Papua New Guinea | 600 (470–750) | 20.2 (16–25) | 1965 (1500–2498) | 24.4 (18.7–30.8) | 20.5 (8.8–32.7) |
| Samoa | 43 (34–54) | 27.9 (22–34) | 62 (48–78) | 30.5 (23.6–38.1) | 9.6 (−1.4 to 21.7) |
| Solomon Islands | 43 (32–55) | 16.1 (12.7–19.9) | 107 (82–138) | 18.8 (14.7–23.6) | 16.7 (4.7–29.3) |
| Tokelau | 0 (0–1) | 29.1 (23.5–35.3) | 0 (0–1) | 35.2 (27.1–43) | 20.9 (8.2–34.1) |
| Tonga | 32 (26–41) | 36.3 (29.1–44.1) | 40 (31–50) | 38.1 (30–47.1) | 4.9 (−6.1 to 16) |
| Tuvalu | 2 (1–2) | 19.4 (15.6–23.6) | 3 (2–4) | 24.9 (19.4–31.2) | 28.7 (15.4–42.5) |
| Vanuatu | 26 (20–34) | 19.6 (15.7–24.3) | 61 (47–79) | 21.6 (17.1–27) | 10.3 (−0.9 to 21.1) |
| South Asia | 275130 (224850–327858) | 33.3 (27.4–39.1) | 707324 (561389–851613) | 44.1 (35.2–52.3) | 32.5 (22.2–44.4) |
| Bangladesh | 24121 (20185–28856) | 36.1 (29.9–44.5) | 71220 (57288–86643) | 49.5 (40.5–59.9) | 37.2 (20.8–55.2) |
| Bhutan | 128 (105–152) | 33.4 (27.7–40.4) | 351 (291–413) | 52.5 (43.4–61.8) | 57.3 (40.6–74.5) |
| India | 215290 (174604–258117) | 32.6 (26.5–38.5) | 543633 (426581–666885) | 43.7 (34.8–52.4) | 34.1 (23.4–47.2) |
| Nepal | 4639 (3800–5535) | 32.8 (27.1–38.7) | 12700 (10335–15268) | 47 (38.7–55.5) | 43.3 (28.4–58.9) |
| Pakistan | 30953 (24700–37723) | 35.3 (28.9–42) | 79419 (62272–99110) | 41.9 (32.8–51.1) | 18.8 (7.2–32.7) |
| Southeast Asia | 71882 (58934–87138) | 23.2 (19.2–27.8) | 161148 (129923–196703) | 26.4 (21.5–32.2) | 13.9 (4.9–22.6) |
| Cambodia | 653 (532–793) | 13.4 (10.6–17.1) | 2097 (1660–2603) | 16.9 (13.6–21.3) | 25.7 (6.5–42.8) |
| Indonesia | 25853 (20738–31864) | 22.2 (17.8–27.5) | 56303 (43952–69676) | 25.9 (20.4–32.3) | 16.8 (6.3–27.6) |
| Lao People's Democratic Republic | 274 (219–338) | 12.9 (10.3–16.4) | 899 (705–1118) | 17.3 (13.8–22.1) | 33.5 (17.6–52.7) |
| Malaysia | 6336 (5286–7558) | 45.3 (38–54.1) | 11512 (9386–13763) | 41.6 (34.3–50.1) | −8.1 (−18 to 3.4) |
| Maldives | 24 (20–29) | 19.9 (16.1–24.7) | 126 (103–154) | 32.6 (26.7–39.7) | 64.4 (45.5–83.6) |
| Mauritius | 105 (83–130) | 12.4 (9.9–15.5) | 454 (358–561) | 33.2 (26.4–40.2) | 168.7 (136.7–208.2) |
| Myanmar | 3522 (2853–4280) | 15.3 (12.4–19.5) | 9597 (7601–11913) | 20.5 (16.3–25.8) | 34.2 (15–52.8) |
| Philippines | 11424 (9094–14213) | 28.7 (23–35.5) | 26692 (21028–33541) | 29.2 (23.1–36.7) | 1.7 (−6.6 to 11) |
| Seychelles | 30 (25–35) | 44.4 (37.2–52.1) | 37 (30–45) | 37.4 (30.7–45.2) | −15.8 (−26 to −5.1) |
| Sri Lanka | 6793 (5642–7966) | 53.2 (44.7–63.3) | 7723 (6199–9439) | 33.9 (27.4–41.1) | −36.3 (−44.6 to −26.6) |
| Thailand | 2831 (2300–3451) | 6.5 (5.4–7.8) | 12540 (9653–15819) | 14.6 (11.5–18.3) | 124.7 (95.2–156.1) |
| Timor-Leste | 71 (57–85) | 18.6 (15.1–23.1) | 228 (177–283) | 22 (17.5–27.6) | 18.1 (4.1–33.4) |
| Viet Nam | 13863 (11262–16669) | 28.1 (23–34.8) | 32716 (26226–39502) | 36.4 (29.4–44.1) | 29.4 (15.5–44.7) |
| Southern Latin America | 45572 (37399–54031) | 95.5 (78.6–114.8) | 71294 (58138–87063) | 97.1 (79.7–116.6) | 1.7 (−7.4 to 11.9) |
| Uruguay | 2599 (2060–3147) | 77.5 (62.2–93.4) | 2809 (2194–3522) | 68.8 (55–85.4) | −11.2 (−19.8 to −2.5) |
| Argentina | 33293 (27151–39992) | 103.2 (84.2–124.7) | 54959 (44664–67318) | 111.5 (91.4–134.3) | 8.1 (−3.7 to 21) |
| Chile | 9677 (7850–11683) | 77.4 (62.9–92.1) | 13523 (10858–16430) | 69.6 (56.1–84) | −10 (−17.3 to −1.4) |
| Southern Sub-Saharan Africa | 32779 (25102–42456) | 74.7 (57.8–95.4) | 59173 (45574–75807) | 79.3 (60.6–101.2) | 6.2 (1.6–10.8) |
| Botswana | 865 (644–1157) | 83 (62.2–108.4) | 1886 (1409–2420) | 87.6 (65–112.8) | 5.6 (−3.3 to 15.4) |
| Eswatini | 488 (360–666) | 79.6 (59.7–104.7) | 813 (612–1049) | 84.7 (62.4–109.8) | 6.4 (−1.6 to 14.7) |
| Lesotho | 948 (696–1251) | 73.8 (55.6–94.8) | 1265 (952–1652) | 79.1 (58.6–102.8) | 7.1 (−1.3 to 16.9) |
| Namibia | 963 (726–1280) | 85.4 (64–111.2) | 1937 (1446–2493) | 92.3 (68.8–118.1) | 8.1 (0.5–17.1) |
| South Africa | 21282 (16042–27709) | 66.8 (50.1–86.8) | 40675 (30825–52665) | 74.2 (56–95.2) | 11 (7–14.9) |
| Zimbabwe | 8232 (6619–10307) | 107.4 (83.9–134.5) | 12597 (9608–15989) | 102.5 (76.7–130.3) | −4.6 (−15.8 to 6.4) |
| Tropical Latin America | 129068 (107180–153317) | 109.2 (90.8–130.5) | 265449 (215717–318397) | 115.2 (94–139.5) | 5.6 (−2.1 to 13) |
| Brazil | 126608 (104963–150538) | 110.3 (91.5–132) | 261353 (212331–313638) | 116.8 (95.2–141.4) | 5.9 (−1.8 to 13.4) |
| Paraguay | 2459 (2005–2998) | 71.6 (58.3–84.8) | 4096 (3248–4972) | 62.8 (49.2–76.3) | −12.2 (−22.6 to −1.8) |
| Western Europe | 398930 (331476–479953) | 92.2 (77.7–107.2) | 505908 (402197–616812) | 83.1 (68.8–97.4) | −9.8 (−16.5 to −2.9) |
| Andorra | 43 (35–52) | 91.8 (75.7–109.8) | 99 (77–121) | 96.9 (80–115.8) | 5.5 (−4 to 16.6) |
| Austria | 21397 (17071–25289) | 215.9 (181.6–247.7) | 13481 (10832–16467) | 112.8 (94.8–134.9) | −47.7 (−53.7 to −39.7) |
| Belgium | 8357 (6702–10224) | 76 (62.4–91.4) | 7266 (5796–9398) | 45.7 (37.2–55.6) | −39.9 (−47.1 to −31.8) |
| Cyprus | 276 (220–333) | 37.6 (30–45.1) | 592 (463–730) | 43.9 (35.2–53.5) | 16.8 (3.8–31.1) |
| Denmark | 1311 (1088–1583) | 27.1 (22.1–32.9) | 1649 (1260–2136) | 22 (17.4–27.1) | −18.6 (−31.1 to −6.2) |
| Finland | 2312 (1892–2767) | 52.2 (42.6–63.9) | 3210 (2437–4124) | 40.4 (32.2–49.1) | −22.5 (−33.4 to −9.1) |
| France | 116088 (95114–139879) | 168 (140.1–198) | 154115 (116659–199508) | 148 (118.8–178.2) | −11.9 (−21.4 to −1.7) |
| Germany | 51008 (41543–61359) | 66.1 (54.3–79.8) | 76337 (59577–96197) | 71.2 (57.2–86.4) | 7.6 (−3.3 to 19.9) |
| Greece | 5181 (4190–6149) | 47.8 (39.4–56.8) | 4831 (3966–5781) | 42.5 (35.3–51.6) | −11.1 (−20.8 to −0.1) |
| Iceland | 68 (56–82) | 27.5 (22.4–33.7) | 67 (52–83) | 17 (13.3–21.1) | −38.1 (−47.2 to −28.5) |
| Ireland | 3187 (2646–3807) | 88.6 (73.8–105.6) | 4176 (3376–5170) | 70.6 (57.7–85) | −20.3 (−28.2 to −11.1) |
| Israel | 2115 (1710–2582) | 42.1 (34.1–50.8) | 3334 (2663–4015) | 31.5 (24.9–38.3) | −25.3 (−33.4 to −16.2) |
| Italy | 88767 (66242–115214) | 124.5 (99.9–153.8) | 87251 (64792–113555) | 83.9 (66.6–102.9) | −32.6 (−42.1 to −22.5) |
| Luxembourg | 321 (260–393) | 88.1 (71.9–106) | 377 (290–479) | 51.4 (40.6–63.7) | −41.6 (−48.7 to −32.9) |
| Malta | 314 (263–373) | 89.4 (74.3–105.9) | 246 (190–312) | 39.7 (32.2–48.1) | −55.6 (−60.4 to −49.7) |
| Monaco | 49 (38–63) | 101.2 (83.7–120.6) | 57 (43–75) | 89.8 (73.3–107.8) | −11.2 (−19.5 to −2.2) |
| Netherlands | 11545 (9662–13638) | 74.5 (63.3–88.7) | 14243 (11154–17939) | 62.6 (51.3–75.3) | −16 (−26.3 to −5.7) |
| Norway | 2128 (1746–2559) | 58.6 (47.6–71.6) | 4204 (3296–5259) | 75.3 (59.9–92.7) | 28.5 (15.1–43.7) |
| Portugal | 4180 (3388–4994) | 41.9 (34.3–49.9) | 6781 (5010–8766) | 41 (32.2–50) | −2.3 (−15.6 to 12.7) |
| San Marino | 31 (25–38) | 114.2 (96.3–132.8) | 50 (39–65) | 93 (76.6–111.1) | −18.5 (−25.7 to −10.5) |
| Spain | 39442 (31840–47959) | 92.1 (76.1–108.3) | 63827 (51565–77344) | 96.1 (79.3–111.8) | 4.3 (−10.8 to 22.7) |
| Sweden | 13086 (10959–15342) | 158.5 (131.8–186.4) | 16323 (12745–20262) | 136.4 (109.7–163.9) | −14 (−22.2 to −4.5) |
| Switzerland | 2804 (2260–3412) | 41.2 (33–49.8) | 3231 (2531–4144) | 28.6 (22.6–35) | −30.6 (−37.2 to −22.9) |
| United Kingdom | 24591 (20345–28873) | 46.1 (38–55.8) | 39715 (32022–47394) | 57 (46.1–68.5) | 23.5 (14.3–31) |
| Western Sub-Saharan Africa | 94906 (72416–123752) | 64.9 (49–83.7) | 256671 (191889–340859) | 68.8 (51.3–89.8) | 6 (2.4–9.5) |
| Benin | 1842 (1353–2509) | 51.9 (37–69.6) | 5640 (4061–7743) | 55.6 (39.4–74.8) | 7.2 (−2.5 to 16.4) |
| Burkina Faso | 3574 (2552–4883) | 50.7 (35.5–68.6) | 9110 (6492–12458) | 53.5 (37.3–72.1) | 5.5 (−3.2 to 14.5) |
| Cabo Verde | 168 (119–224) | 59.3 (42.6–79.1) | 359 (256–481) | 66.7 (47.7–88.8) | 12.5 (2.8–22.4) |
| Cameroon | 4128 (2925–5705) | 51.1 (36–67.8) | 14250 (10197–19834) | 57.6 (41–77) | 12.7 (3.6–24.4) |
| Chad | 2146 (1538–2892) | 47.8 (34.2–63.9) | 6373 (4453–8700) | 51.4 (37.2–69.6) | 7.4 (−1.6 to 17.2) |
| Côte d'Ivoire | 4650 (3311–6351) | 52.9 (37–70.3) | 12288 (8791–16856) | 56.4 (40.9–74.6) | 6.7 (−2.1 to 16.4) |
| Gambia | 390 (283–536) | 53.9 (39.3–73) | 1065 (758–1440) | 57.3 (40.9–76.4) | 6.3 (−2.7 to 15.9) |
| Ghana | 6649 (5064–8251) | 60.8 (46.5–77.8) | 18200 (13079–24136) | 66 (47.6–88) | 8.5 (−7.7 to 26.2) |
| Guinea | 2347 (1689–3172) | 49.8 (35.6–66.2) | 5347 (3858–7351) | 53.2 (38.6–71.1) | 6.8 (−3 to 18.1) |
| Guinea-Bissau | 351 (252–479) | 47.5 (34.7–63.9) | 795 (564–1088) | 51.3 (36.8–68.2) | 7.8 (−1.5 to 16.4) |
| Liberia | 981 (704–1324) | 53 (37.8–72.2) | 2425 (1744–3284) | 56.9 (40.3–76.1) | 7.2 (−2.2 to 17.6) |
| Mali | 3168 (2282–4269) | 48.6 (34.8–65.4) | 9060 (6465–12465) | 52.3 (38–70.4) | 7.4 (−2.2 to 17.1) |
| Mauritania | 899 (645–1243) | 56.1 (39.9–75) | 2127 (1528–2905) | 60.3 (43.3–81.9) | 7.5 (−2.4 to 18) |
| Niger | 2837 (2013–3922) | 50.9 (36.2–68.1) | 9023 (6425–12462) | 52.6 (37.5–71.1) | 3.3 (−5.4 to 12.6) |
| Nigeria | 55750 (43096–72159) | 79.2 (60.6–102.2) | 148254 (114124–194139) | 84 (64–108.6) | 6.1 (3–9.3) |
| Sao Tome and Principe | 57 (41–76) | 60.7 (43.5–80.2) | 120 (86–164) | 65.8 (48–87.7) | 8.4 (−1 to 18.5) |
| Senegal | 1916 (1356–2679) | 34.7 (24.1–47.4) | 4628 (3256–6338) | 37.1 (25.5–50.6) | 6.9 (−1.6 to 16.2) |
| Sierra Leone | 1686 (1199–2287) | 52.8 (37.1–71) | 3826 (2733–5166) | 56.3 (41–74.5) | 6.6 (−2.8 to 15.1) |
| Togo | 1362 (980–1882) | 51.7 (36.9–68.8) | 3777 (2711–5145) | 55.3 (39.9–74.4) | 6.8 (−1.5 to 15.7) |
