# Supplementary material for: Global, regional, and national burden of cardiomyopathy (including alcoholic cardiomyopathy and others) from 1990 to 2021: An analysis of data from the global burden of disease study 2021 and forecast to 2040
Source: PLoS One. 2026 Jan 30;21(1):e0341687. doi: 10.1371/journal.pone.0341687 (PMC12858021; doi:10.1371/journal.pone.0341687)
Supplement: S15 Table — (DOCX) [file pone.0341687.s026.docx]

**S15 Table.** **1990–2021 Global and national DALYs trends in other cardiomyopathy burden.**

| location_name | Number_1990 | ASR per 100,000_1990 | Number_2021 | ASR per 100,000_2021 | Percentage change in the ASRs per 100,000 |
| --- | --- | --- | --- | --- | --- |
| Global | 207149 (180555–227935) | 6.1 (5.3–6.7) | 306262 (276191–333295) | 3.7 (3.4–4.1) | −38.5 (−43.3 to −32.3) |
| Andean Latin America | 517 (440–592) | 2 (1.7–2.3) | 642 (532–777) | 1.1 (0.9–1.3) | −45.9 (−57 to −30.6) |
| Bolivia (Plurinational State of) | 121 (83–172) | 2.8 (1.9–3.7) | 169 (126–226) | 1.9 (1.4–2.6) | −30.6 (−50.1 to 2.2) |
| Ecuador | 114 (102–126) | 1.9 (1.7–2.1) | 215 (167–277) | 1.4 (1.1–1.7) | −27.7 (−46.5 to −2.1) |
| Peru | 282 (234–336) | 1.9 (1.5–2.3) | 258 (194–356) | 0.8 (0.6–1) | −59.6 (−72.3 to −35.9) |
| Australasia | 1309 (1219–1397) | 6 (5.6–6.4) | 1223 (1067–1358) | 2.2 (1.9–2.4) | −63.2 (−66.9 to −59.5) |
| Australia | 1122 (1041–1200) | 6.2 (5.7–6.6) | 995 (856–1112) | 2.1 (1.8–2.3) | −66.3 (−69.7 to −62.5) |
| New Zealand | 187 (165–205) | 5 (4.5–5.5) | 228 (204–251) | 2.8 (2.5–3.1) | −44.5 (−50.6 to −37.1) |
| Caribbean | 1076 (852–1275) | 3.9 (3.2–4.6) | 1953 (1568–2337) | 3.7 (3–4.5) | −5.3 (−19 to 8.8) |
| Antigua and Barbuda | 2 (2–2) | 3.9 (3.5–4.3) | 5 (5–6) | 5.5 (5.1–6) | 42.6 (27.2–61.2) |
| Bahamas | 11 (10–12) | 6.5 (5.8–7.4) | 25 (20–31) | 6.5 (5.3–8) | −0.5 (−21.9 to 29.1) |
| Barbados | 15 (14–17) | 5.2 (4.6–5.9) | 23 (19–28) | 4.6 (3.7–5.7) | −11.4 (−31.8 to 11.4) |
| Belize | 5 (4–5) | 3.9 (3.2–4.6) | 13 (11–15) | 4.4 (3.8–4.9) | 12.2 (−12 to 43.1) |
| Bermuda | 3 (3–4) | 5.4 (4.4–6.3) | 7 (6–9) | 4.9 (4.1–6) | −8.6 (−29 to 19.7) |
| Cuba | 195 (171–215) | 1.9 (1.7–2.2) | 564 (487–637) | 2.9 (2.5–3.2) | 48.4 (25.4–76.2) |
| Dominica | 8 (7–10) | 14.1 (11.3–17.6) | 13 (10–16) | 16.3 (13.4–20) | 15.8 (−17.5 to 57.8) |
| Dominican Republic | 144 (119–181) | 2.9 (2.3–4.1) | 270 (210–349) | 2.7 (2.1–3.5) | −8.1 (−34.1 to 30.2) |
| Grenada | 5 (5–6) | 6.7 (6–7.4) | 10 (8–11) | 9.5 (8.3–10.6) | 42.3 (20.5–63.6) |
| Guyana | 33 (30–37) | 7.6 (6.8–8.6) | 44 (33–56) | 7.3 (5.7–9.1) | −4.9 (−30.1 to 24.9) |
| Haiti | 286 (115–456) | 7.8 (3.4–12.4) | 560 (249–864) | 7.2 (3.3–11.1) | −7.9 (−34 to 34.5) |
| Jamaica | 73 (64–83) | 3.9 (3.4–4.4) | 123 (95–154) | 3.9 (3–4.8) | 0 (−25.1 to 28.9) |
| Puerto Rico | 195 (180–208) | 5.6 (5.2–6) | 118 (97–139) | 1.6 (1.3–1.9) | −71.7 (−76.7 to −66.7) |
| Saint Kitts and Nevis | 4 (4–4) | 11.3 (10.5–12.1) | 6 (5–7) | 10.5 (8.9–12.1) | −6.6 (−22.4 to 9.4) |
| Saint Lucia | 9 (8–9) | 10.6 (9.7–11.7) | 17 (14–20) | 7.4 (6.2–8.8) | −30.2 (−42.4 to −14.7) |
| Saint Vincent and the Grenadines | 2 (2–2) | 2.5 (2.2–2.7) | 4 (3–4) | 2.9 (2.5–3.2) | 16.5 (1.1–34.4) |
| Suriname | 12 (10–15) | 4.5 (3.7–5.7) | 23 (18–31) | 3.9 (3–5.1) | −14.2 (−36.9 to 17.3) |
| Trinidad and Tobago | 32 (29–35) | 4 (3.6–4.4) | 56 (43–69) | 3.1 (2.4–3.8) | −22.6 (−40.7 to −1.7) |
| United States Virgin Islands | 7 (5–8) | 8.4 (6.6–10.5) | 7 (5–9) | 4.6 (3.4–5.9) | −45.5 (−60.2 to −23.9) |
| Central Asia | 1811 (1543–2091) | 3.7 (3.2–4.3) | 9930 (8275–11681) | 12 (10.2–13.9) | 220.5 (151.1–308.2) |
| Armenia | 67 (53–87) | 2.9 (2.2–3.7) | 119 (88–154) | 2.9 (2.1–3.7) | 1.8 (−35.6 to 56.1) |
| Azerbaijan | 638 (429–865) | 12.6 (8.4–17.2) | 1374 (753–2130) | 13.1 (7.8–19.8) | 3.9 (−41.4 to 80.5) |
| Georgia | 236 (177–296) | 4.1 (3.1–5.2) | 305 (243–375) | 5.3 (4.2–6.5) | 27.7 (−11.7 to 91) |
| Kazakhstan | 226 (187–267) | 1.8 (1.5–2.1) | 6559 (5307–7875) | 38.2 (31.1–45.2) | 2064.8 (1583.7–2697.7) |
| Kyrgyzstan | 10 (7–13) | 0.3 (0.2–0.4) | 55 (42–71) | 1.2 (1–1.6) | 311.3 (185–508.5) |
| Mongolia | 95 (52–150) | 9.2 (5.2–14.8) | 245 (176–331) | 10.9 (8.1–14.5) | 18.2 (−28 to 102.7) |
| Tajikistan | 49 (37–66) | 1.6 (1.2–2.2) | 80 (57–110) | 1.2 (0.9–1.6) | −26.9 (−51.9 to 6.9) |
| Turkmenistan | 370 (330–417) | 17.8 (15.7–20.1) | 619 (488–784) | 14.2 (11.2–17.9) | −20.2 (−37.4 to 3.7) |
| Uzbekistan | 118 (96–142) | 0.9 (0.7–1.1) | 573 (473–681) | 2.1 (1.8–2.5) | 141.2 (84.4–220.4) |
| Central Europe | 18381 (17065–19871) | 14.7 (13.6–15.9) | 21842 (19646–23969) | 9.5 (8.5–10.4) | −35.7 (−41.5 to −27.4) |
| Albania | 187 (153–225) | 11.7 (9.4–13.8) | 301 (209–413) | 7.6 (5.2–10.3) | −35.1 (−57.2 to −5.2) |
| Bosnia and Herzegovina | 389 (251–563) | 12.7 (8–18.2) | 847 (555–1181) | 13.7 (8.9–19) | 7.3 (−31 to 58.4) |
| Bulgaria | 177 (157–198) | 2.3 (2.1–2.5) | 542 (449–652) | 4 (3.3–4.8) | 74 (41.6–114) |
| Croatia | 239 (207–269) | 5.1 (4.4–5.7) | 359 (296–427) | 3.7 (3–4.4) | −27.8 (−40 to −13) |
| Czechia | 386 (350–423) | 3 (2.7–3.3) | 521 (430–620) | 2.4 (2–2.9) | −19.1 (−34.6 to −0.2) |
| Hungary | 1460 (1267–1651) | 12.1 (10.5–13.6) | 664 (552–781) | 3.2 (2.7–3.8) | −73.6 (−77.8 to −68.1) |
| Montenegro | 108 (76–151) | 19.4 (13.6–26.5) | 191 (128–256) | 23.5 (15.6–31.6) | 21.2 (−17.5 to 100.7) |
| North Macedonia | 280 (212–361) | 18.2 (13.7–23.5) | 510 (354–698) | 20.5 (13.8–28.5) | 12.8 (−25.9 to 69.3) |
| Poland | 5615 (5315–5887) | 14 (13.1–14.7) | 7204 (6310–8019) | 9.3 (8.2–10.4) | −33.3 (−39.6 to −25.7) |
| Romania | 6099 (5293–7206) | 30.2 (26–35.3) | 7194 (6237–8264) | 18.6 (16–21.5) | −38.6 (−50.5 to −20.1) |
| Serbia | 1747 (1316–2169) | 23.2 (17.2–28.5) | 2328 (1553–3284) | 13.6 (9.2–19.3) | −41.4 (−61.6 to −14.3) |
| Slovakia | 187 (145–253) | 3.4 (2.6–4.6) | 327 (237–418) | 3.6 (2.6–4.6) | 5.9 (−24.1 to 46) |
| Slovenia | 1213 (1091–1325) | 51.5 (46.1–56.5) | 535 (437–654) | 9.7 (7.9–11.9) | −81.2 (−84 to −77.6) |
| Central Latin America | 1968 (1863–2064) | 2.2 (2–2.3) | 3784 (3321–4307) | 1.6 (1.4–1.8) | −27.1 (−36.8 to −16.8) |
| Colombia | 594 (562–625) | 3.2 (3–3.3) | 1022 (850–1214) | 1.9 (1.6–2.2) | −40.8 (−50.6 to −29.7) |
| Costa Rica | 82 (76–88) | 4.6 (4.2–5) | 154 (135–171) | 2.8 (2.5–3.1) | −39.3 (−46.8 to −31.5) |
| El Salvador | 74 (58–90) | 2 (1.6–2.5) | 93 (74–117) | 1.4 (1.1–1.8) | −29.4 (−49.6 to 3.9) |
| Guatemala | 107 (82–123) | 2.6 (2.3–3.1) | 170 (144–199) | 1.5 (1.3–1.8) | −42.9 (−53.9 to −31.3) |
| Honduras | 74 (62–87) | 3.1 (2.5–3.7) | 187 (134–248) | 3.1 (2.2–4.2) | 0.9 (−31.7 to 40.3) |
| Mexico | 391 (371–412) | 0.7 (0.7–0.8) | 1163 (1030–1328) | 1 (0.9–1.1) | 34.5 (19.4–51) |
| Nicaragua | 48 (40–60) | 2.2 (1.8–2.6) | 78 (62–95) | 1.6 (1.3–2) | −24.6 (−41.1 to 0.6) |
| Panama | 66 (61–72) | 4.3 (3.9–4.6) | 174 (137–209) | 3.9 (3–4.7) | −9.1 (−29.1 to 9.1) |
| Venezuela (Bolivarian Republic of) | 532 (482–576) | 5.4 (4.7–5.9) | 743 (562–940) | 2.7 (2–3.4) | −49.9 (−61.8 to −37) |
| Central Sub-Saharan Africa | 3626 (2297–5294) | 15.7 (9.6–24.9) | 7527 (4266–12001) | 14.2 (7.8–23.6) | −9.5 (−33.1 to 22.8) |
| Angola | 664 (405–950) | 15.9 (9.5–24.5) | 1660 (951–2344) | 14.1 (8.1–20.6) | −11.4 (−41.8 to 41.5) |
| Central African Republic | 204 (121–307) | 17.7 (10.3–28.7) | 353 (186–577) | 16.1 (8.3–27.1) | −9.2 (−36.6 to 23.9) |
| Congo | 170 (106–225) | 16.4 (10.6–23) | 328 (191–461) | 13 (7.8–17.8) | −20.8 (−46.2 to 13.6) |
| Democratic Republic of the Congo | 2469 (1504–3776) | 15.4 (8.6–25.9) | 5020 (2676–8735) | 14.4 (7.5–26.1) | −6.4 (−34.7 to 34.8) |
| Equatorial Guinea | 32 (19–47) | 16.5 (9.8–26.1) | 58 (27–96) | 10.4 (5.2–17) | −36.6 (−65.1 to 32.3) |
| Gabon | 86 (54–122) | 16.2 (10.3–23.8) | 109 (58–157) | 11.4 (6.3–16.5) | −29.4 (−50 to 5.5) |
| East Asia | 6504 (3854–10605) | 0.8 (0.5–1.4) | 15440 (11999–20469) | 0.9 (0.7–1.1) | 7.5 (−45.1 to 89.5) |
| China | 5979 (3395–9952) | 0.7 (0.4–1.3) | 14712 (11193–19630) | 0.9 (0.7–1.1) | 15.3 (−44.4 to 116.8) |
| Democratic People's Republic of Korea | 212 (138–342) | 1.6 (0.9–2.7) | 387 (257–591) | 1.4 (0.9–2.2) | −8.1 (−40.8 to 43.4) |
| Taiwan (Province of China) | 313 (297–330) | 2.4 (2.2–2.5) | 341 (300–377) | 0.9 (0.8–0.9) | −64.2 (−67.8 to −60.7) |
| Eastern Europe | 6305 (5828–6849) | 2.9 (2.7–3.1) | 30863 (28266–34287) | 10.2 (9.4–11.3) | 249 (214.9–294.5) |
| Belarus | 458 (317–659) | 3.9 (2.7–5.5) | 462 (363–577) | 3.6 (2.8–4.4) | −8.5 (−40.1 to 40.3) |
| Estonia | 102 (85–124) | 5.4 (4.5–6.5) | 128 (109–148) | 4.8 (4.1–5.6) | −9.8 (−26.2 to 11.4) |
| Latvia | 248 (211–292) | 7.8 (6.6–9.1) | 475 (402–574) | 13.3 (11.2–16.3) | 70.9 (43.1–108.4) |
| Lithuania | 140 (117–167) | 3.3 (2.7–3.9) | 326 (276–382) | 6.7 (5.7–7.9) | 104.8 (69.6–151.4) |
| Republic of Moldova | 70 (61–82) | 1.7 (1.4–1.9) | 141 (119–168) | 2.6 (2.2–3.1) | 55.5 (29.5–84.9) |
| Russian Federation | 3070 (2849–3228) | 2.3 (2.1–2.5) | 24023 (22025–26500) | 11.7 (10.8–12.9) | 398.9 (347.1–485.2) |
| Ukraine | 2217 (1910–2600) | 4 (3.5–4.7) | 5308 (4160–6722) | 7.8 (6–9.8) | 93.5 (46.1–154.2) |
| Eastern Sub-Saharan Africa | 5022 (3044–5836) | 4.7 (3.3–5.6) | 8816 (5516–10938) | 4 (2.6–4.8) | −15.8 (−39.1 to 7.4) |
| Burundi | 212 (127–281) | 6.2 (4–8.4) | 283 (166–386) | 4.4 (2.8–6) | −29.3 (−58.8 to 7.6) |
| Comoros | 16 (7–21) | 5.4 (2.8–7.2) | 20 (12–29) | 3.8 (2.2–5.3) | −30.4 (−54.6 to −0.2) |
| Djibouti | 10 (6–15) | 5 (3.1–7.1) | 31 (17–48) | 4 (2.3–5.8) | −20.2 (−46.8 to 22) |
| Eritrea | 99 (60–134) | 5.4 (3.3–7.9) | 170 (106–244) | 4.8 (3.1–6.7) | −9.8 (−48.3 to 35.5) |
| Ethiopia | 1117 (674–1488) | 4 (2.5–5.5) | 1674 (1060–2225) | 3 (1.9–4) | −25.5 (−57.2 to 15) |
| Kenya | 334 (220–405) | 3 (2.2–3.7) | 785 (479–1028) | 3.1 (1.9–3.9) | 0.9 (−23.6 to 32.7) |
| Madagascar | 586 (346–730) | 8.1 (5.3–10) | 1078 (647–1483) | 6.8 (4.3–8.9) | −16.6 (−40.1 to 16.3) |
| Malawi | 239 (130–298) | 4 (2.6–4.8) | 383 (227–518) | 4 (2.5–5.2) | 0.5 (−26.4 to 34.8) |
| Mozambique | 270 (207–337) | 3.5 (2.7–4.2) | 552 (383–771) | 3.8 (2.7–5.2) | 9.5 (−24 to 59.1) |
| Rwanda | 268 (154–355) | 6.5 (4.2–8.9) | 304 (172–441) | 4.1 (2.4–5.7) | −37.4 (−65.5 to −2) |
| Somalia | 218 (129–309) | 5.2 (3.4–7.7) | 443 (250–686) | 4.6 (2.8–7.2) | −13.2 (−46.4 to 26.7) |
| South Sudan | 202 (121–266) | 5.5 (3.4–7.5) | 263 (148–394) | 4.3 (2.6–5.9) | −21.8 (−46.2 to 19.8) |
| Uganda | 499 (295–683) | 4.8 (3.3–6.4) | 767 (428–1083) | 3.5 (2.1–4.9) | −27 (−52 to 7.2) |
| United Republic of Tanzania | 765 (472–956) | 5.3 (3.5–6.6) | 1347 (806–1845) | 4.1 (2.6–5.6) | −22.6 (−46.2 to 8.3) |
| Zambia | 184 (104–235) | 4.5 (2.9–5.9) | 710 (267–1110) | 7.7 (3.4–11.8) | 72.8 (−30.3 to 177.8) |
| High-income Asia Pacific | 9859 (8864–10401) | 5.7 (5.1–6.1) | 9501 (7713–10611) | 1.7 (1.5–1.9) | −70.4 (−72.2 to −68.5) |
| Brunei Darussalam | 11 (9–13) | 9.4 (7.3–11.7) | 18 (15–21) | 6.1 (5–7.4) | −35.3 (−50.6 to −17.9) |
| Japan | 9166 (8257–9640) | 6.2 (5.5–6.6) | 8186 (6450–9167) | 1.8 (1.6–2) | −71 (−72.4 to −69.5) |
| Republic of Korea | 563 (386–723) | 2.3 (1.6–3.1) | 1208 (955–1449) | 1.4 (1.1–1.7) | −40.7 (−57.7 to −7.2) |
| Singapore | 121 (114–127) | 5.7 (5.3–6) | 89 (78–98) | 1.1 (1–1.2) | −80.7 (−82.7 to −78.6) |
| High-income North America | 25376 (23602–26588) | 7.3 (6.8–7.6) | 24247 (21349–26027) | 3.7 (3.3–4) | −49.2 (−52.1 to −46.8) |
| Canada | 853 (785–916) | 2.7 (2.5–2.9) | 1216 (1059–1344) | 1.6 (1.5–1.8) | −39.4 (−45.5 to −32.8) |
| Greenland | 5 (4–6) | 13 (10.9–15.5) | 5 (4–6) | 7.9 (6.2–9.8) | −39 (−54.3 to −20.6) |
| United States of America | 24518 (22804–25710) | 7.8 (7.3–8.1) | 23026 (20252–24776) | 4 (3.6–4.2) | −49 (−51.9 to −46.4) |
| North Africa and Middle East | 6643 (4775–8970) | 2.9 (2.1–4.8) | 8610 (6980–12268) | 2 (1.6–2.9) | −33.2 (−48.1 to −8.4) |
| Afghanistan | 182 (88–349) | 2.3 (1.1–4.8) | 328 (203–556) | 2.4 (1.3–4.8) | 4.8 (−38 to 61.2) |
| Algeria | 353 (228–511) | 2.6 (1.5–4.3) | 567 (287–846) | 1.9 (1–2.9) | −25.8 (−48.1 to 6.4) |
| Bahrain | 13 (12–15) | 8.6 (7–10.1) | 30 (23–39) | 4.9 (3.9–5.9) | −43.3 (−55.9 to −25.3) |
| Egypt | 1751 (948–2980) | 4.1 (2.5–8.1) | 1483 (1065–2541) | 2.5 (1.8–4.5) | −40.9 (−57.3 to −9.6) |
| Iran (Islamic Republic of) | 600 (456–806) | 1.8 (1.3–2.5) | 875 (740–1271) | 1.2 (1–1.9) | −30.2 (−47.9 to −8.2) |
| Iraq | 577 (411–794) | 5.2 (3.6–7.6) | 941 (717–1295) | 4 (3–6.1) | −21.6 (−48.7 to 15.3) |
| Jordan | 18 (13–22) | 1 (0.7–1.2) | 29 (23–36) | 0.4 (0.3–0.5) | −56.1 (−69.3 to −35.3) |
| Kuwait | 17 (15–18) | 2.1 (1.9–2.3) | 13 (10–17) | 0.6 (0.4–0.7) | −73.8 (−78.7 to −68.6) |
| Lebanon | 34 (18–57) | 1.6 (0.9–2.8) | 65 (47–89) | 1 (0.7–1.4) | −38.8 (−70.3 to 24.7) |
| Libya | 88 (57–122) | 2.3 (1.4–3.3) | 81 (51–117) | 1.7 (1.1–2.4) | −24.5 (−50.8 to 13.5) |
| Morocco | 454 (285–678) | 2.5 (1.4–4.5) | 634 (324–1064) | 2.1 (1–3.4) | −17.4 (−42 to 23.6) |
| Oman | 68 (48–98) | 8.5 (5.7–12.5) | 99 (63–133) | 5 (3.6–6.6) | −40.5 (−64.4 to 1.3) |
| Palestine | 56 (41–72) | 5.4 (3.9–7.1) | 80 (62–109) | 3.6 (2.7–4.6) | −34.2 (−52.8 to −2) |
| Qatar | 8 (6–10) | 8.2 (4.9–10.4) | 23 (12–33) | 2.9 (1.8–4) | −64.2 (−77 to −41.1) |
| Saudi Arabia | 506 (363–679) | 7.3 (5–10.3) | 873 (650–1160) | 4.8 (3.6–6.2) | −34.7 (−58 to 10.1) |
| Sudan | 370 (198–653) | 2.5 (1.4–4.6) | 476 (309–742) | 1.9 (1.1–3.1) | −23.3 (−52.4 to 29.7) |
| Syrian Arab Republic | 232 (159–321) | 2.6 (1.5–4.1) | 215 (110–342) | 1.9 (1.1–3.1) | −27.1 (−53.2 to 14.1) |
| Tunisia | 116 (74–178) | 2.1 (1.2–3.5) | 209 (84–350) | 1.8 (0.7–3) | −13.7 (−47.7 to 29.1) |
| Turkey | 950 (646–1319) | 2.3 (1.6–3.4) | 1051 (830–1551) | 1.2 (1–1.8) | −46.9 (−63.5 to −18.1) |
| United Arab Emirates | 52 (37–73) | 9.2 (6.6–12.6) | 160 (123–210) | 6.1 (4.6–7.8) | −33.6 (−54.9 to −9.6) |
| Yemen | 196 (104–323) | 2.4 (1.3–4.7) | 368 (236–611) | 2.2 (1.2–3.9) | −9.5 (−41 to 50.6) |
| Oceania | 148 (93–211) | 4.1 (2.7–6) | 375 (247–543) | 4.1 (2.8–6.1) | 0.5 (−21.1 to 30.1) |
| American Samoa | 2 (2–2) | 7.8 (6.3–9.8) | 3 (3–4) | 7.3 (5.9–8.9) | −7 (−29 to 23.4) |
| Cook Islands | 0 (0–0) | 0.5 (0.4–0.7) | 0 (0–0) | 0.4 (0.3–0.5) | −33.5 (−54.3 to −5.5) |
| Fiji | 13 (10–16) | 3.2 (2.5–4) | 24 (18–31) | 3.2 (2.5–4.1) | 0.1 (−28.8 to 38.8) |
| Guam | 3 (3–4) | 4.3 (3.6–5.1) | 4 (3–5) | 2.1 (1.9–2.5) | −49.5 (−58.5 to −38.9) |
| Kiribati | 2 (1–3) | 4.6 (2.3–7.3) | 3 (2–4) | 4.5 (2.6–6.7) | −2.8 (−26.8 to 42.7) |
| Marshall Islands | 1 (1–1) | 5.1 (2.8–7) | 2 (1–2) | 4.5 (2.8–6.4) | −11.8 (−34.7 to 26.9) |
| Micronesia (Federated States of) | 3 (2–4) | 6.2 (3.6–9.4) | 3 (2–5) | 4.9 (3.3–6.8) | −21.5 (−43 to 13.9) |
| Nauru | 0 (0–0) | 6.4 (4.5–8.4) | 0 (0–1) | 7.4 (5.1–10.2) | 14.6 (−24.6 to 67.7) |
| Niue | 0 (0–0) | 4.6 (3.3–6.1) | 0 (0–0) | 4.5 (3.7–5.5) | −2.3 (−30.7 to 37.2) |
| Northern Mariana Islands | 1 (1–1) | 4.1 (3.2–5.5) | 2 (1–2) | 4.3 (3.4–5.2) | 5.4 (−21.2 to 40.1) |
| Palau | 0 (0–0) | 3.5 (2.6–4.6) | 1 (0–1) | 2.9 (2.2–3.7) | −17.4 (−39.7 to 13.2) |
| Papua New Guinea | 97 (56–147) | 4.1 (2.4–6.4) | 286 (175–439) | 4.4 (2.7–7.1) | 7.5 (−24.1 to 56.1) |
| Samoa | 4 (3–6) | 4.8 (3.3–6.7) | 6 (4–8) | 4 (3–5.3) | −16.3 (−36 to 14.8) |
| Solomon Islands | 5 (3–8) | 4 (2.4–6.6) | 14 (8–21) | 4 (2.5–6.3) | 1.3 (−26.9 to 36.7) |
| Tokelau | 0 (0–0) | 5.3 (3.4–7.5) | 0 (0–0) | 5.6 (3.9–7.6) | 4.9 (−26.1 to 58) |
| Tonga | 2 (1–2) | 3.3 (2.3–4.5) | 2 (2–3) | 2.7 (2–3.4) | −18.1 (−41.7 to 14.9) |
| Tuvalu | 0 (0–1) | 5.6 (3.1–7.8) | 0 (0–1) | 4.4 (3.1–5.8) | −20.6 (−39.8 to 18.5) |
| Vanuatu | 3 (2–5) | 4.4 (2.5–6.6) | 8 (5–11) | 4.1 (2.5–5.8) | −6.8 (−29.7 to 23.5) |
| South Asia | 26628 (13855–37658) | 4.7 (2.4–6.8) | 66308 (52242–83817) | 4.9 (3.9–6.2) | 5.6 (−16.8 to 68.9) |
| Bangladesh | 3141 (1415–5023) | 6.7 (3.1–11.2) | 7599 (4935–11042) | 6.3 (4.1–9.1) | −7.1 (−37.1 to 69.3) |
| Bhutan | 12 (5–22) | 5.5 (2.2–10.6) | 32 (21–48) | 5.5 (3.8–8.3) | 1.2 (−33.8 to 105.2) |
| India | 19904 (10691–27711) | 4.3 (2.3–6) | 50638 (40344–64206) | 4.6 (3.7–5.9) | 8.7 (−14.8 to 71.6) |
| Nepal | 503 (224–807) | 5.6 (2.5–9.8) | 1092 (752–1681) | 5.4 (3.7–8.3) | −4.6 (−35.8 to 70) |
| Pakistan | 3068 (1424–4463) | 5.1 (2.4–7.9) | 6947 (4655–9411) | 6 (4–8.3) | 17 (−13 to 92.8) |
| Southeast Asia | 8080 (6180–9669) | 3.9 (3–4.7) | 16778 (14363–19543) | 3.1 (2.7–3.6) | −19.9 (−37.9 to 6.9) |
| Cambodia | 119 (67–175) | 3.3 (1.8–4.8) | 288 (200–394) | 3.1 (2.2–4.1) | −5.4 (−31.8 to 63.4) |
| Indonesia | 2656 (1849–3805) | 3.3 (2.2–4.6) | 6712 (4991–8690) | 4.1 (3.1–5.2) | 24.5 (−11.5 to 82.9) |
| Lao People's Democratic Republic | 73 (36–110) | 4.6 (2.4–7) | 141 (99–196) | 3.8 (2.7–5.3) | −17.8 (−44.1 to 42.9) |
| Malaysia | 477 (365–585) | 5.2 (3.9–6.5) | 791 (664–918) | 3.2 (2.7–3.8) | −38.6 (−55.6 to −8.7) |
| Maldives | 3 (1–4) | 3.8 (2.1–5.4) | 8 (6–10) | 2.6 (1.9–3.4) | −30.8 (−56.5 to 34.6) |
| Mauritius | 9 (8–10) | 1.4 (1.3–1.5) | 71 (64–77) | 4.3 (3.9–4.7) | 203.6 (173.1 to 234.2) |
| Myanmar | 861 (537–1253) | 4.8 (3–6.9) | 1555 (1198–2073) | 3.9 (3–5.3) | −17.6 (−44.3 to 29.3) |
| Philippines | 1424 (915–1652) | 6.8 (3.6–8.2) | 2878 (2004–3377) | 4.3 (2.8–5.1) | −37 (−48.4 to −18.1) |
| Seychelles | 6 (5–7) | 11.2 (9.3–13.1) | 6 (5–8) | 6.1 (5.1–7.5) | −45.1 (−56.9 to −27.8) |
| Sri Lanka | 901 (615–1091) | 9.2 (6.6–11.2) | 565 (350–1044) | 2.4 (1.5–4.3) | −74.3 (−84.4 to −34.1) |
| Thailand | 148 (91–247) | 0.5 (0.3–0.8) | 677 (488–877) | 0.7 (0.5–0.9) | 49 (−21 to 157.2) |
| Timor-Leste | 9 (5–15) | 4 (2.2–6.2) | 28 (19–39) | 4.1 (2.7–5.7) | 1.3 (−31.1 to 59.6) |
| Viet Nam | 1383 (972–1966) | 4 (2.8–5.8) | 3036 (2226–3890) | 3.8 (2.8–4.9) | −5.6 (−40.6 to 47.2) |
| Southern Latin America | 6098 (5528–6686) | 14.3 (12.8–15.7) | 7255 (6537–7887) | 8.1 (7.4–8.8) | −42.9 (−49 to −36.8) |
| Uruguay | 344 (308–379) | 9 (8.1–9.8) | 273 (244–297) | 4.4 (4–4.8) | −50.8 (−56.3 to −44.6) |
| Argentina | 5233 (4701–5798) | 17.6 (15.7–19.5) | 6376 (5727–6973) | 11.1 (10–12.2) | −36.8 (−44 to −29) |
| Chile | 521 (494–553) | 5.4 (5.1–5.7) | 606 (553–657) | 2.4 (2.2–2.6) | −55.5 (−59.2 to −50.9) |
| Southern Sub-Saharan Africa | 3752 (2948–4482) | 15.1 (11.4–18.4) | 6521 (5816–7299) | 12.7 (11.4–14.4) | −15.6 (−32.3 to 11) |
| Botswana | 77 (57–101) | 16.4 (12.4–21.8) | 139 (102–183) | 11.6 (8.6–15.2) | −29.4 (−54.1 to 10.1) |
| Eswatini | 40 (30–53) | 15.6 (11.8–20.7) | 55 (39–78) | 10.9 (7.7–15.3) | −30 (−53.6 to 1.9) |
| Lesotho | 100 (67–146) | 13.9 (9.3–20.2) | 115 (80–165) | 12.6 (8.7–17.7) | −9.1 (−44 to 41.7) |
| Namibia | 87 (67–107) | 16.4 (12.5–20.4) | 155 (115–197) | 13.4 (10.3–17.2) | −18.2 (−42.6 to 14.1) |
| South Africa | 2940 (2219–3524) | 15.1 (10.8–18.4) | 5126 (4513–5815) | 12.4 (11.1–14.2) | −17.7 (−34.3 to 11.3) |
| Zimbabwe | 508 (402–625) | 15.2 (11.9–18.8) | 931 (708–1204) | 15.3 (11.7–19.6) | 0.8 (−27.4 to 48.4) |
| Tropical Latin America | 11650 (11056–12137) | 14 (13.1–14.7) | 16167 (14668–17227) | 6.5 (5.9–6.9) | −53.7 (−56.2 to −51) |
| Brazil | 11518 (10929–11992) | 14.3 (13.3–15) | 15954 (14466–17002) | 6.6 (5.9–7) | −54 (−56.5 to −51.3) |
| Paraguay | 132 (106–158) | 5.9 (4.6–7.1) | 213 (165–272) | 3.8 (2.9–4.9) | −35.4 (−54 to −7.9) |
| Western Europe | 50510 (44986–53732) | 8.8 (7.8–9.4) | 31015 (26582–33808) | 2.8 (2.4–3) | −68.5 (−70.6 to −66.3) |
| Andorra | 4 (2–5) | 8.6 (5.9–12.6) | 8 (6–11) | 4.7 (3.2–6.3) | −45.6 (−66.5 to −16.1) |
| Austria | 3968 (3542–4267) | 33.8 (30.2–36.4) | 1423 (1193–1586) | 6.2 (5.3–6.9) | −81.7 (−83.2 to −80.1) |
| Belgium | 1271 (1122–1375) | 8.2 (7.3–8.9) | 834 (684–934) | 2.7 (2.3–3) | −67 (−70.2 to −64) |
| Cyprus | 39 (27–51) | 7.8 (5.1–10.8) | 58 (44–74) | 3.5 (2.5–4.4) | −55.9 (−70.4 to −31) |
| Denmark | 162 (144–179) | 2 (1.8–2.2) | 123 (106–140) | 0.9 (0.8–1.1) | −53.2 (−59.6 to −45.8) |
| Finland | 201 (163–229) | 3 (2.5–3.5) | 286 (244–319) | 2 (1.8–2.3) | −33 (−42.7 to −14.9) |
| France | 6231 (5679–6665) | 7.1 (6.5–7.6) | 5159 (4317–5716) | 2.7 (2.3–3) | −62.3 (−65.8 to −58.8) |
| Germany | 6903 (5813–8000) | 5.4 (4.6–6.3) | 7019 (5838–7927) | 3 (2.6–3.4) | −44.2 (−52.8 to −33.6) |
| Greece | 764 (693–818) | 5.7 (5.1–6.1) | 743 (656–810) | 3 (2.7–3.2) | −47.6 (−51.7 to −42.8) |
| Iceland | 8 (7–9) | 2.6 (2.4–2.9) | 7 (6–8) | 1.1 (0.9–1.2) | −59.1 (−63.9 to −53.2) |
| Ireland | 449 (411–480) | 12.3 (11.2–13.2) | 277 (232–315) | 3.3 (2.8–3.8) | −73.1 (−76.1 to −69.7) |
| Israel | 144 (132–157) | 3.3 (3–3.6) | 134 (114–149) | 1 (0.9–1.1) | −70 (−73.6 to −66.2) |
| Italy | 20486 (17847–21967) | 25.3 (21.7–27.2) | 4684 (3842–5297) | 2.6 (2.2–2.9) | −89.7 (−90.8 to −88.6) |
| Luxembourg | 41 (38–43) | 8.1 (7.5–8.7) | 27 (23–31) | 2.2 (1.9–2.5) | −72.9 (−75.8 to −69.6) |
| Malta | 48 (43–51) | 12.8 (11.4–13.9) | 22 (18–25) | 2 (1.7–2.2) | −84.5 (−86.3 to −82.6) |
| Monaco | 9 (6–12) | 11.1 (7.4–14.3) | 7 (5–9) | 5.7 (3.9–7.6) | −48.8 (−63.3 to −22.9) |
| Netherlands | 1601 (1424–1736) | 8.1 (7.2–8.8) | 907 (761–1012) | 2.3 (2–2.6) | −71.6 (−74 to −68.8) |
| Norway | 84 (78–89) | 1.4 (1.3–1.5) | 163 (143–178) | 1.5 (1.4–1.7) | 10.7 (2.5–18.1) |
| Portugal | 476 (439–509) | 4.1 (3.8–4.4) | 579 (500–645) | 2.1 (1.9–2.4) | −48.2 (−54 to −42) |
| San Marino | 3 (2–4) | 8.8 (6.4–11) | 3 (2–4) | 2.9 (1.8–4) | −67.5 (−79.4 to −48.5) |
| Spain | 5122 (4627–5526) | 10.1 (9.1–10.9) | 4839 (4078–5391) | 4.2 (3.7–4.6) | −58.5 (−62.5 to −54.2) |
| Sweden | 425 (397–448) | 3.1 (2.9–3.2) | 374 (319–426) | 1.6 (1.4–1.8) | −47.3 (−53.3 to −40.7) |
| Switzerland | 358 (305–408) | 3.3 (2.9–3.8) | 306 (252–353) | 1.3 (1.1–1.5) | −60.7 (−67.1 to −51.5) |
| United Kingdom | 1673 (1574–1735) | 2 (1.9–2.1) | 3006 (2639–3240) | 2.2 (2–2.3) | 6.5 (0.1–12.7) |
| Western Sub-Saharan Africa | 11885 (8833–15893) | 13.7 (10.4–19.2) | 17464 (13283–20888) | 8.6 (6.9–10.2) | −37.3 (−51.5 to −20.3) |
| Benin | 228 (160–329) | 10.8 (7.6–16.3) | 393 (283–509) | 7.3 (5.4–9.6) | −32.6 (−50.3 to −5.4) |
| Burkina Faso | 627 (405–1008) | 15.5 (10.2–26.3) | 1046 (760–1524) | 11.8 (8.6–17.6) | −23.8 (−46.2 to 8.5) |
| Cabo Verde | 11 (8–15) | 4.2 (2.9–6.2) | 15 (11–20) | 3.4 (2.5–4.4) | −20.5 (−55.2 to 31.6) |
| Cameroon | 581 (388–759) | 13.4 (9.1–18) | 1140 (812–1515) | 8.7 (6.5–11.7) | −35.2 (−55.7 to −4.1) |
| Chad | 359 (232–607) | 12.5 (7.9–21.8) | 612 (415–919) | 9.8 (6.7–15.3) | −21.7 (−41.6 to 12.2) |
| Côte d'Ivoire | 560 (358–736) | 13.5 (9.2–18.5) | 1036 (721–1382) | 8.9 (6.6–11.6) | −34.2 (−50.8 to −10.4) |
| Gambia | 47 (29–68) | 13 (8.5–19.3) | 95 (70–122) | 9.4 (7–12.1) | −28.1 (−51.3 to 5.1) |
| Ghana | 1013 (797–1323) | 16.2 (12.7–21.7) | 2444 (1742–3281) | 15 (10.9–20) | −7.5 (−42.3 to 37.6) |
| Guinea | 431 (288–640) | 12.8 (8.7–19.8) | 570 (400–783) | 9.6 (6.8–13.5) | −24.7 (−46.5 to 11.5) |
| Guinea-Bissau | 63 (40–91) | 15.2 (9.8–22.1) | 79 (56–103) | 10.1 (7.2–13.4) | −33.5 (−52.4 to −9.1) |
| Liberia | 165 (107–237) | 13.6 (9.2–20.7) | 187 (125–268) | 8.7 (5.9–12.6) | −36.3 (−57.2 to −3.8) |
| Mali | 412 (266–631) | 10.4 (6.9–17) | 679 (494–987) | 7 (5.2–10.3) | −32.3 (−51.2 to −5.6) |
| Mauritania | 128 (87–167) | 13.1 (8.9–17.5) | 173 (122–233) | 8.3 (5.9–11) | −36.9 (−54.8 to −7.5) |
| Niger | 356 (219–575) | 12.7 (7.9–22.8) | 726 (444–1159) | 9 (5.7–14.8) | −28.8 (−50 to 0.8) |
| Nigeria | 5967 (4154–8193) | 14 (10–19.8) | 6857 (4846–8970) | 7.1 (5.4–9.1) | −49.5 (−64.6 to −30.1) |
| Sao Tome and Principe | 6 (4–9) | 10.2 (6.4–14.5) | 8 (6–11) | 7.4 (5.6–9.4) | −27.9 (−49.3 to 9.4) |
| Senegal | 454 (293–657) | 13.5 (9.3–20.3) | 710 (514–956) | 9.3 (6.8–12.9) | −31.2 (−52.6 to −3.8) |
| Sierra Leone | 306 (196–436) | 13.8 (8.9–20.9) | 368 (245–505) | 9 (6.4–12.5) | −34.5 (−53.8 to −5.9) |
| Togo | 171 (112–235) | 13.1 (8.9–18.6) | 327 (224–456) | 8.9 (6.5–12.2) | −32.1 (−52.6 to −3.7) |
