# Supplementary material for: Global, regional, and national burden of cardiomyopathy (including alcoholic cardiomyopathy and others) from 1990 to 2021: An analysis of data from the global burden of disease study 2021 and forecast to 2040
Source: PLoS One. 2026 Jan 30;21(1):e0341687. doi: 10.1371/journal.pone.0341687 (PMC12858021; doi:10.1371/journal.pone.0341687)
Supplement: Attachment — Submitted filename: Response to Reviewers.docx [file pone.0341687.s028.docx]

**Subject: Revised Manuscript Submission: [PONE-D-25-32695] - [Global, regional, and national burden of cardiomyopathy (including alcoholic cardiomyopathy and others) from 1990 to 2021: an analysis of data from the global burden of disease study 2021 and forecast to 2040]**

Dear Dr. Claudio Alberto Dávila-Cervantes and Reviewers,

Thank you for the opportunity to revise our manuscript and for the constructive feedback from the editors and reviewers. We sincerely appreciate the editors and reviewers for their insightful comments and constructive suggestions, which have greatly helped us to improve the quality of our work.

We have carefully addressed all the comments and requirements. Our point-by-point responses are detailed below. All changes in the manuscript have been highlighted in red in the ‘Revised Manuscript with Track Changes’ file, and an unmarked version is also provided.

**Responses to Journal Requirements:**

1. **Style Requirements:** We have reformatted the entire manuscript according to the PLOS ONE style templates.
2. **Financial Disclosure:** As the funders had no role in our study, we have included the following statement in our cover letter: *“The funders had no role in study design, data collection and analysis, decision to publish, or preparation of the manuscript.”*
3. **Copyright of Map Figures:**

We thank the editor for raising this important point. We wish to provide a comprehensive explanation to clarify that the world maps in our manuscript are fully compliant with the CC BY 4.0 license. **The maps in our manuscript were generated using the map_data("world")function from the R mapspackage.** This is a critical point: the geographic boundary data within this package is **sourced directly from the Natural Earth database** (naturalearthdata.com).

- **Natural Earth** is a public domain resource that is **explicitly recommended on your journal's guidelines page** for creating CC BY-compatible maps.
- Data from Natural Earth is explicitly dedicated to the public domain, meaning it is free to use, adapt, and publish without restriction, making it perfectly compatible with the CC BY 4.0 license.

Therefore, we confirm that our maps do not utilize any copyrighted services. We believe this explanation and the accompanying caption updates resolve this requirement conclusively.

4. **Figure Quality:** We have used the LAW compression function to modify the figures formats, sizes, and clarity in the manuscripts and supplementary materials to meet PLOS ONE requirements.
5. **Citation of Suggested Works:** We have reviewed the literature and ensured that all relevant and necessary citations are included.

**Responses to Reviewers' Comments:**

**Reviewer #1:**

We thank the reviewer for the positive assessment and valuable suggestions for enhancement. The reviewer found the methodology sound and conclusions supported, but suggested improving the readability of the text, deepening the conclusions, and providing a clearer rationale for methodological choices.

**Response:**

1. **Language and Readability:** We have thoroughly revised the manuscript, particularly the Abstract and Results sections, to reduce numerical density and improve readability. Detailed data arrays have been moved to supplementary tables, and the text now focuses on summarizing key trends.
2. **Methodological Clarity:** We have expanded the Methods section and added two references (numbered 28 and 29) [1,2] (Page 9, Lines 175 to Page 10, Lines 196) to provide a clearer rationale for the choice of the BAPC model and to ensure all statistical descriptions are unambiguous and reproducible.
3. **Discussion and Implications:** The discussion section provides a refined elaboration of the original text, detailing the specific implications of our research findings in the fields of public health and clinical practice, thereby enhancing the impact of the manuscript.

**Reviewer #2:**

We sincerely thank the reviewer for the thorough, critical, and constructive assessment of our manuscript. The comments have been invaluable in helping us identify the weaknesses in our initial submission and have guided us in significantly strengthening the study's conceptual foundation, methodological rigor, and overall scholarly contribution. We have undertaken a major revision to address all the concerns raised. Our point-by-point responses and the specific actions taken are detailed below.

**Comment 1:** On the conceptual contribution and the descriptive nature of the study. Reviewer's comment: *"There is a fundamental mismatch between the stated research objectives and the study design... the work remains largely descriptive and does not adequately support the broader inferential claims..."*

**Response:** We acknowledge and thank the reviewer for this critical insight. We agree that our initial manuscript did not sufficiently articulate the unique value of performing a focused assessment on the broader category of "cardiomyopathy (including AC and OC)" within the GBD framework. To address this:

•**Action taken (Introduction revised):** We have entirely rewritten the Introduction section (specifically, the final paragraph outlining the study aims on Page 5, Lines 95 to Page 6, Lines 120) to more clearly position our work. We now state that while GBD data is publicly available, our study provides the **first comprehensive analysis to specifically consolidate, trend, and forecast the burden of** cardiomyopathy (as a collective group of diseases) across time, geography, and socio-demographic indices. We emphasize that this synthesis adds value by moving beyond isolated disease metrics to provide a unified view of the total public health burden attributable to all forms of cardiomyopathy, which is crucial for health policy planning that often addresses disease groups rather than individual ICD codes.

**Comment 2:** On the study of population and generalizability.

**Reviewer's comment:** *"The study population is insufficiently defined from an epidemiological perspective... diagnostic heterogeneity, underreporting, or regional variation..."*

**Response:** This is a valid limitation of any study using GBD data. We now explicitly acknowledge and discuss this.

•Action taken (New Limitations subsection): As strongly suggested by the reviewer's comments, we have added a Refinement of the limitations section in the Discussion part to more accurately reflect the limitations presented in this paper (Page 42, Lines 603 to Page 43, Lines 627). In this section, we openly discuss:

1.	The inherent limitations of the GBD data, including potential misclassification and under-ascertainment in regions with weak vital registration systems.

2.	The fact that our analysis is based on modeled estimates rather than raw data, and thus is subject to the assumptions and limitations of the GBD modeling framework.

**Comment 3:** On the analytical contribution, robustness, and sensitivity analysis. Reviewer's comment: *"The analytical contribution of the study is limited... no sensitivity analyses or alternative modelling approaches are presented. Consequently, the robustness of the reported trends and future projections cannot be adequately assessed."*

**Response:** This is a crucial point, and we have taken substantial steps to enhance the analytical rigor and transparency of our work.

**•Action taken (Sensitivity analysis performed):** To directly address the concern about robustness, we have conducted a comprehensive sensitivity analysis for our forecasts. Specifically, we complemented the primary Bayesian Age-Period-Cohort (BAPC) model with an alternative, well-established time-series forecasting method: the Autoregressive Integrated Moving Average (ARIMA) model.

**Results added:** We have added a new subsection within the Results section titled "Sensitivity Analysis" (Page 36, Lines 476 to Lines 487). This section details the ARIMA model specification, including describing how we use AMIRA to perform forecasting of TC from 2022 to 2040.

**Results presented:** The results of this sensitivity analysis, comparing the projections from the BAPC and ARIMA models for key metrics (e.g., global TC’s ASPR), are now presented in a new Supplementary Figure (S11 Fig.). We note that the projections from both models showed consistent trends, which significantly strengthens the credibility of our findings.

**•Action taken (Methodological justification enhanced):** We have expanded the Methods section and added two references (numbered 28 and 29) [1,2] to provide a clearer rationale for selecting the BAPC model over other approaches (Page 9, Lines 175 to Page 10, Lines 196). We explain that the BAPC model is particularly suited for long-term epidemiological forecasts as it explicitly accounts for the separate effects of age, time period, and birth cohort, which are critical drivers of chronic disease trends.

**Comment 4:** On overinterpretation of results. Reviewer's comment: *"The conclusions appear to overinterpret descriptive and model-based results... such interpretations are not sufficiently supported."*

**Response:** We agree with the reviewer that our initial discussion sometimes overstepped the boundaries of what descriptive data can support. We have carefully revised the tone and content.

**•Action taken (Language calibrated):** We have systematically gone through the Discussion and Conclusion sections and replaced any language that implied causation or direct policy prescription with more cautious, descriptive, and suggestive language. For example, we changed phrases like "our findings show that policy must..." to "the observed rising trends suggest that future health planning may need to be considered...".

**•Action taken (Focus on explanation):** We have reframed the Discussion to focus on explaining the possible reasons behind the observed trends (e.g., citing literature on improved diagnostics, aging populations, changes in risk factors) rather than making definitive claims about their implications for direct intervention.

**Comment 5:** On reproducibility and methodological detail. Reviewer's comment: *"Key methodological details... are insufficiently described. This limits the ability of independent researchers to replicate or critically evaluate the analyses..."*

**Response:** We have taken concrete steps to ensure the full reproducibility of our study.

**•Action taken (Methods expanded):** We have added significantly more detail to the Methods section. This includes specifying the software and packages used and explicitly defining how BAPC modle were calculated.

**•Action taken (Code and data availability):** In accordance with PLOS ONE's data policy, and to ensure full transparency, we can transparently upload R code publicly. The GBD data itself is publicly available from the IHME website (as originally stated).

We believe that the extensive revisions undertaken—including the addition of sensitivity analyses, enhanced methodological description, a refined discussion, and a clear statement of limitations—have fundamentally addressed the concerns raised. The manuscript is now substantially stronger, more rigorous, and more transparent. We are deeply grateful for your time and insightful comments, which have been instrumental in improving our work.

Sincerely,

M.D. Yanzhang Tian

Institution: Department of Biliary and Pancreatic Surgery, Shanxi Bethune Hospital, Shanxi Academy of Medical Sciences, Tongji Shanxi Hospital, Third Hospital of Shanxi Medical University

Address: No. 99, Longcheng Street, Taiyuan, Shanxi, China

E-mail: 15802872565@163.com

**References**

1 Pearson-Stuttard J, Guzman-Castillo M, Penalvo JL, et al. Modeling Future Cardiovascular Disease Mortality in the United States: National Trends and Racial and Ethnic Disparities. *Circulation*. 2016;133(10):967-978. doi:10.1161/CIRCULATIONAHA.115.019904
2 Kiyoshige E, Ogata S, O'Flaherty M, et al. Projections of future coronary heart disease and stroke mortality in Japan until 2040: a Bayesian age-period-cohort analysis. *Lancet Reg Health West Pac.* 2022;31:100637. doi:10.1016/j.lanwpc.2022.100637
